# Sex without crossing over in the yeast *Saccharomycodes ludwigii*

**DOI:** 10.1186/s13059-021-02521-w

**Published:** 2021-11-03

**Authors:** Ioannis A. Papaioannou, Fabien Dutreux, France A. Peltier, Hiromi Maekawa, Nicolas Delhomme, Amit Bardhan, Anne Friedrich, Joseph Schacherer, Michael Knop

**Affiliations:** 1 Center for Molecular Biology of Heidelberg University (ZMBH), Heidelberg, Germany; 2 Université de Strasbourg, CNRS, GMGM UMR 7156, Strasbourg, France; 3 Current affiliation: Faculty of Agriculture, Kyushu University, Fukuoka, Japan; 4 Umeå Plant Science Centre, Department of Forest Genetics and Plant Physiology, Swedish University of Agricultural Sciences, Umeå, Sweden; 5 Institut Universitaire de France (IUF), Paris, France; 6 German Cancer Research Center (DKFZ), DKFZ-ZMBH Alliance, Heidelberg, Germany

**Keywords:** Achiasmate meiosis, Automixis, Crossing over, Intratetrad mating, Meiotic recombination, Linkage disequilibrium, Mutation accumulation, Mutation rate, *Saccharomycodes ludwigii*

## Abstract

**Background:**

Intermixing of genomes through meiotic reassortment and recombination of homologous chromosomes is a unifying theme of sexual reproduction in eukaryotic organisms and is considered crucial for their adaptive evolution. Previous studies of the budding yeast species *Saccharomycodes ludwigii* suggested that meiotic crossing over might be absent from its sexual life cycle, which is predominated by fertilization within the meiotic tetrad.

**Results:**

We demonstrate that recombination is extremely suppressed during meiosis in *Sd. ludwigii*. DNA double-strand break formation by the conserved transesterase Spo11, processing and repair involving interhomolog interactions are required for normal meiosis but do not lead to crossing over. Although the species has retained an intact meiotic gene repertoire, genetic and population analyses suggest the exceptionally rare occurrence of meiotic crossovers in its genome. A strong AT bias of spontaneous mutations and the absence of recombination are likely responsible for its unusually low genomic GC level.

**Conclusions:**

*Sd. ludwigii* has followed a unique evolutionary trajectory that possibly derives fitness benefits from the combination of frequent mating between products of the same meiotic event with the extreme suppression of meiotic recombination. This life style ensures preservation of heterozygosity throughout its genome and may enable the species to adapt to its environment and survive with only minimal levels of rare meiotic recombination. We propose *Sd. ludwigii* as an excellent natural forum for the study of genome evolution and recombination rates.

**Supplementary Information:**

The online version contains supplementary material available at 10.1186/s13059-021-02521-w.

## Background

Sex is the prevailing reproductive mode throughout the eukaryotic tree of life [1]. At its core lies a periodic ploidy cycling, accomplished through meiosis, during which haploid gametes are produced, and mating, which ensures the restoration of the original ploidy level. Meiosis, thought to have arisen early in the eukaryotic evolution, is a ubiquitous attribute of sexual life cycles [2]. During the first meiotic division (meiosis I), parental chromosomes are recombined and separated, while in the second division sister chromatids segregate (meiosis II). Reassortment and recombination of homologous chromosomes in meiosis I lead to novel genetic constellations in the offspring. These function as substrates for natural selection, which can promote advantageous and purge deleterious genetic combinations [3, 4]. However, the considerable biological costs of sex render its widespread occurrence paradoxical, and the questions of its evolutionary origin and functions are outstanding enigmas in biology [5–7].

**Fig. 1:**
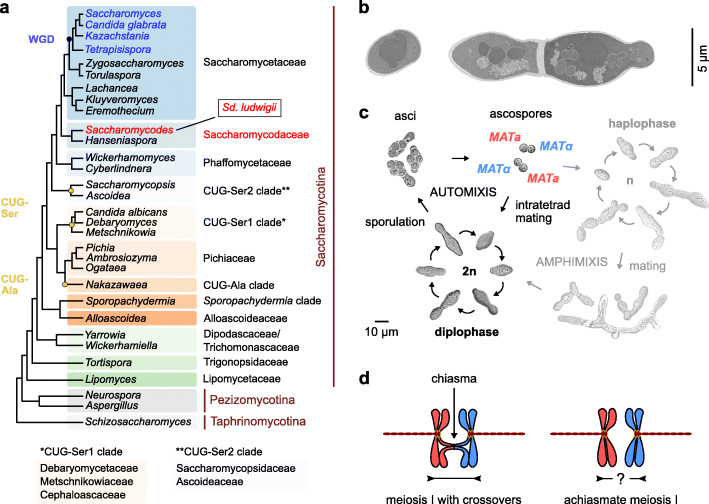
The budding yeast *Saccharomycodes ludwigii*. **a** Classification of *Sd. ludwigii* in the yeast phylogeny. WGD: whole-genome duplication; CUG-Ser1/2 and CUG-Ala: clades deviating from the universal genetic code. **b** Transmission electron microscopy images of representative *Sd. ludwigii* vegetative cells (reference strain NBRC 1722). **c** Life cycle of *Sd. ludwigii*. Brightfield images of strains NBRC 1722 and its diploid progenitor NBRC 1721 are shown. **d** Chiasmata in meiotic prophase I contribute to spindle stabilization and faithful segregation of homologous chromosomes. Meiosis in *Sd. ludwigii*, however, has been suggested to be achiasmate [11, 12]

*Saccharomyces cerevisiae* (baker’s yeast) has been used as a premier model organism to gain major insights into the mechanisms of meiosis [8, 9]. Following pairing of homologous chromosomes, meiotic recombination is initiated in prophase I with the formation of DNA double-strand breaks (DSBs) by the topoisomerase-like transesterase Spo11 [10]. Several pathways mediate the repair of these DSBs and pathway choice is regulated by a multitude of meiosis-specific factors that act in concert with the DNA repair machinery. One possibility involves the use of the homologous chromosome as repair template, which can lead to the generation of chimeric DNA molecules. This process may involve either reciprocal exchange of the chromosomal regions that flank the DSB site (crossover, CO) or gene conversion without reciprocal exchange (non-crossover, NCO) [8, 9]. Crossing over establishes chiasmata (Fig. 1d), which ensure the physical interconnection of bivalents that is important for the faithful segregation of homologous chromosomes in meiosis I [13]. In many, but not all, organisms, this is facilitated by the synaptonemal complex (SC), which mediates stable synapsis [9].

The extent of genetic exchange in meiosis and thus generation of genomic diversity depend on the frequency and distribution of COs along chromosomes. Homeostatic regulation controls these parameters in many organisms [14, 15], with different outcomes between species. These range from one obligatory CO per chromosome in *Caenorhabditis elegans* [16] to 10 or more in *Schizosaccharomyces pombe* [17], and up to 15 in the longest chromosomes of *S. cerevisiae* [18]. Furthermore, generation of diversity is influenced by the mating behavior (breeding), which can involve gametes of variable genetic relatedness. Outbreeding involves mating between unrelated gametes, ensuring high genetic diversity in the offspring. On the other hand, inbreeding (self-fertilization) refers to mating between gametes that originate from the same individual or clonal line. Inbreeding generally reduces heterozygosity, which can compromise the adaptive potential of populations [19].

Mating of gametes from the same meiotic event represents a particular type of inbreeding referred to as intratetrad mating or automixis [20], the most frequent breeding strategy in *S. cerevisiae* [21–23]. If automixis brings together chromosomes that had been separated during meiosis I (non-sister components), it is described as “central fusion” or “first division restitution”. This has important genetic consequences, since it maintains parental heterozygosity around the centromeres, reducing the risk of deleterious alleles being exposed due to homozygotization [24–26]. The degree to which parental heterozygosity is restituted upon intratetrad mating correlates inversely with the frequency of COs during meiosis. In the absence of crossing over, parental genomes would be fully reconstituted and their heterozygosity maintained. Evolutionary models suggested that high frequency of deleterious mutations could promote automixis in the absence of meiotic recombination [27].

Chromosome segregation in meiosis I without crossing over (achiasmate meiosis) has been observed in one of the two sexes [6, 28] or individual chromosomes [29, 30] of a few organisms. Unusually low overall recombination rates have also been reported in some species [31, 32], but these data should be interpreted with caution since experimental limitations such as sampling bias and insufficient marker coverage may have hindered the identification of COs in many of these cases. In addition, most of these studies could not exclude the possibility of crossing over near chromosome ends [31].

The budding yeast species *Saccharomycodes ludwigii* (Fig. 1a, b) preferentially undergoes intratetrad mating (Fig. 1c), ensured by strong interspore bridges that efficiently keep spores together in pairs of opposite mating types within meiotic tetrads [33, 34]. This organism was used in the early days of yeast genetics for the pioneering description of heterothallism by Øjvind Winge at the Carlsberg laboratory [34, 35]. During his studies, Winge also observed in this species an unusual segregation pattern of two cell morphology markers [34]. The subsequent interpretation by Lindegren was that the two genes were not linked and that “each is so close to the spindle attachment that segregation invariably occurs at the Meiosis I without crossing over” [36]. Later work by the Oshima laboratory surprisingly revealed that the same behavior, which would be consistent with centromere linkage, was displayed by any combination of more than 20 genetic markers tested. These findings led to the conclusion that this “may be due to the absence of crossing over in *Sd. ludwigii*” [11, 12].

Here we explored the extent and types of meiotic interactions between homologous chromosomes in *Sd. ludwigii*. For this, we performed whole-genome sequencing and high-contiguity genome assembly of wild-type strains, which enabled a high-resolution DNA variant segregation analysis of meiosis. We combined bioinformatic analyses of the meiotic gene repertoire with a functional study of key meiotic components in order to derive a better understanding of the meiotic mechanisms in this species. We also searched for signs of recombination on an evolutionary scale between divergent strains of *Sd. ludwigii* in comparison to other species with different levels of meiotic recombination. To gain insights into the relative contributions of mutational pressure and recombination to genome evolution, we determined the genome-wide mutation rate and bias using a mutation accumulation experiment. Our results provide insights into the unique sexual lifestyle of the yeast species *Sd. ludwigii*, and they propose this organism as a particularly suitable model system for the study of the evolution of recombination rates and their impact on genome evolution.

## Results

### The *Sd. ludwigii* genome

**Fig. 2:**
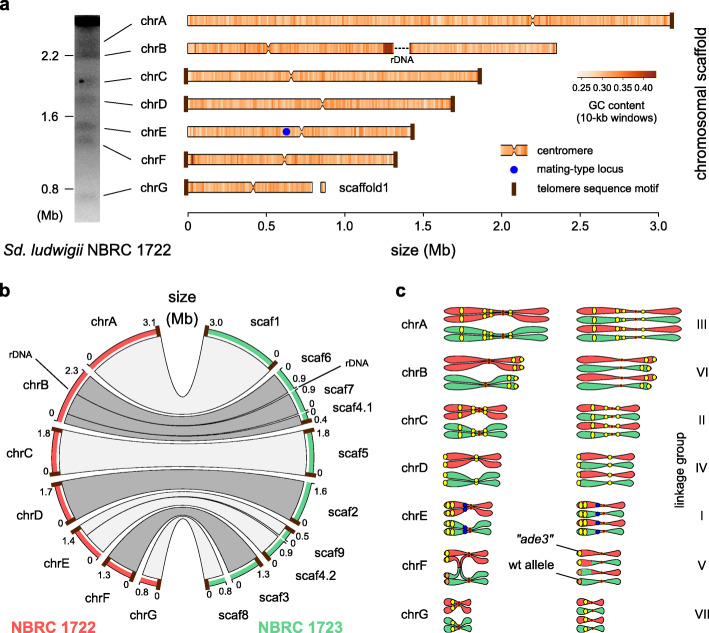
Genome structure of *Sd. ludwigii* and mapping of previously used genetic markers. **a** The nuclear genome of the haploid reference strain NBRC 1722 is organized in 7 chromosomes, resolved as chromosomal bands on a PFGE gel (left) and depicted as bars (right). The only gap in the assembly (in chrB) corresponds to the internal part of the rDNA region, which is flanked by assembled rDNA repeats. **b** Genomic comparison of the *Sd. ludwigii* parental strains used in the previous genetic analyses. **c** The 7 previously defined *Sd. ludwigii* linkage groups [12] were matched to the 7 chromosomes of the reference genome assembly, and 16 of the previously used markers were unambiguously mapped on the assembly (yellow circles). The *MAT* locus is shown as a blue circle (in chrE). One of the rare COs that were previously detected using tetrad analysis was tracked down to a reciprocal exchange event between the marker “*ade3*” (corresponding to the *ADE4* gene) and the centromere of chrF, depicted here as an example

To investigate meiotic recombination in *Sd. ludwigii*, we first used long-read PacBio sequencing to generate a high-contiguity de novo genome assembly of our reference haploid strain NBRC 1722 (Additional file 1: Table S1 [11, 37–39]). The 12.5-Mb assembly consists of 7 chromosomal scaffolds, in concordance with PFGE karyotyping [12, 40] (Fig. 2a) and previous genetic mapping [11, 12]. Gene synteny and DNA motif analyses (Additional file 2: Fig. S1a) enabled the identification of putative point centromeres on all chromosomes. The extremities of all scaffolds share subtelomere-related repetitive sequences and genes, while telomeric DNA repeats were also detected in 9 cases (Additional file 2: Fig. S1b). A single mating-type locus (*MATalpha*) was identified in the centromeric vicinity of chrE (Fig. 2a), in congruence with the known heterothallic nature of the species [34, 35].

The *Sd. ludwigii* genome shares many features with Saccharomycetaceae members that diverged before the whole-genome duplication event (Fig. 1a), including genome size, number of chromosomes, point centromeres, overall gene synteny, number of genes (5031 ORFs), and frequency of introns (3.3%). Remarkably, *Sd. ludwigii* chromosomes exhibit GC levels that are unusually low for a yeast species (30.9% on average; Fig. 2a), as shown by a comparison to 100 yeast and other fungal genomes (Additional file 2: Fig. S2a; Additional file 3: Table S2). The comparative analysis further revealed exceptionally high coverage in *Sd. ludwigii* by AT-rich low-complexity regions and simple sequence repeats (microsatellites), as well as enrichment in transposable elements (Additional file 2: Fig. S2b-e).

Meiotic crossing over could be hindered in chromosomal regions of *Sd. ludwigii* that do not align during meiotic prophase I due to major sequence variation or structural differences of the homologous chromosomes. To investigate whether high genomic dissimilarity could be responsible for the scarcity of COs observed in previous analyses [11, 12] that used our reference strain as one of the parents, we also sequenced and assembled de novo the genome of the second parent used in those experiments (NBRC 1723; Additional file 1: Table S1). The two parental genomes are highly collinear (Fig. 2b) and similar at the DNA sequence level (99.6% identity on average), apart from the terminal region of chrA and the longest part of chrF, which exhibit lower degrees of sequence identity (95.3% on average). These findings exclude the possibility that the absence of COs could be due to major differences between homologous chromosomes.

Another possibility for the explanation of the extreme rarity of previously detected COs could be a non-random chromosomal distribution of the UV-generated genetic markers used in those analyses [11, 12]. By combining the results of our gene prediction and annotation of the reference assembly with the phenotypic gene annotations of *S. cerevisiae* (www.yeastgenome.org), we identified the chromosomal positions of 16 out of the 24 used markers (Fig. 2c). This analysis revealed that all chromosomes were covered by markers, most of which were quite distant from the corresponding centromeres. Therefore, the previously used experimental setup [11, 12] appears sufficient for capturing the majority of COs in the largest part of the genome.

### The *Sd. ludwigii* meiotic gene machinery

**Fig. 3:**
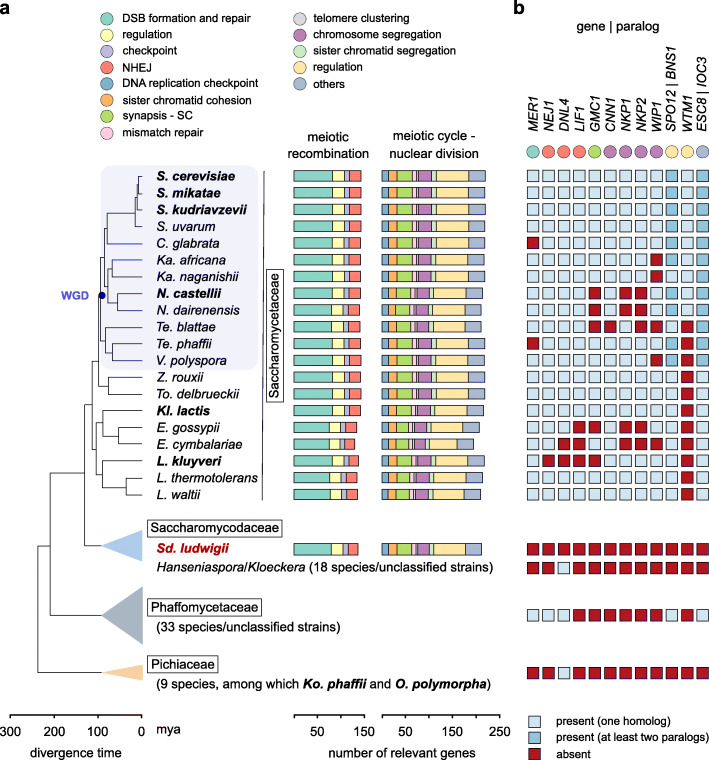
*Sd. ludwigii* possesses a nearly full meiotic gene complement. **a** Meiotic genes of *Sd. ludwigii* and other yeast representatives. Genes were classified into functional groups (top) based on the associated GO terms of their *S. cerevisiae* homologs (www.yeastgenome.org), and the total gene count for each of them is plotted for each species (right). Apart from the presumably asexual species *C. glabrata* [41], *Hanseniaspora jakobsenii*, for which sexual sporulation has not been reported to date [42], and the mitosporic yeasts *Candida mycetangii*, *C. orba*, *C. stellimalicola*, *C. ponderosae*, *C. montana*, and *C. vartiovaarae*, all other 71 species included in this analysis are sexual. The names of species with documented meiotic recombination appear in bold [43–47]. **b** Meiosis/meiotic recombination-related genes that are absent from the reference *Sd. ludwigii* strain are summarized here. Presence or absence of homologs is also indicated for all other species, for comparison. The function of each gene is indicated by a colored circle beneath its name (color code as in **a**)

To gain insight into the genetic causes of the unusual meiotic behavior of *Sd. ludwigii*, we manually refined the gene prediction and we curated the annotation of *Sd. ludwigii* genes based on the available *S. cerevisiae* dataset (www.yeastgenome.org). Gene prediction yielded a total of 5347 genes, of which 5031 are protein-coding ORFs. Among these genes, we identified homologs of 272 out of the 284 genes of *S. cerevisiae* with annotated functions in meiosis (Gene Ontology terms; www.yeastgenome.org), using protein sequence similarity and gene synteny as criteria (Fig. 3a; Additional file 4: Table S3). All genes that are required for wild-type levels of meiotic recombination in *S. cerevisiae* have homologs in *Sd. ludwigii*, with the only exception of *MER1* [48] (Fig. 3a). This gene codes for a meiosis-specific splicing activator for introns in the genes *AMA1*, *HFM1*, *REC107*, and *SPO22* (*ZIP4*), which function in chromosome pairing, meiotic recombination, and cell cycle regulation. Deletion of *MER1* in *S. cerevisiae* abrogates the expression of these 4 target genes and causes major defects in meiotic recombination and progression [48, 49]. However, the Mer1 regulon of *Sd. ludwigii* appears largely rescued, as no introns were detected in 3 of its target genes (*AMA1*, *HFM1,* and *REC107*), while *SPO22* retains a predicted intron. By investigating the phylogenetic distribution of *MER1,* we confirmed its ancestral origin as syntenic homologs were identified in the distant families Phaffomycetaceae, Ascoideaceae, and Saccharomycopsidaceae (Figs. 1a, 3a). Therefore, its absence from *Sd. ludwigii* and the closely related *Hanseniaspora* species must be due to a secondary loss early in the evolution of their family (i.e., Saccharomycodaceae; Fig. 3a).

Apart from *MER1*, no homologs were detected in *Sd. ludwigii* for 11 additional genes with meiotic functions, namely *NEJ1*, *DNL4*, *LIF1*, *GMC1*, *CNN1*, *NKP1*, *NKP2*, *WIP1*, *SPO12/BNS1* (paralogs in *S. cerevisiae*), *WTM1*, and *ESC8/IOC3* (paralogs in *S. cerevisiae*) (Fig. 3b). In contrast to *MER1*, inactivation of any of these genes in *S. cerevisiae* does not abrogate meiotic crossing over. With the exception of *DNL4*, these genes are also absent from members of the Pichiaceae that are known to form COs in meiosis, such as *Komagataella phaffii* and *Ogataea polymorpha* [43, 44]. A phylogenetic analysis suggested that 7 of these genes (i.e., *LIF1*, *NKP1*, *NKP2*, *CNN1*, *WIP1*, *GMC1,* and *WTM1*) are present only in Saccharomycetaceae members, and thus have probably emerged after the separation of the *Sd. ludwigii* lineage. Among the remaining 4 genes, *DNL4* and *NEJ1* (involved in non-homologous end joining, NHEJ [50]) are also missing from *Lachancea kluyveri*, which is capable of meiotic crossing over [45], while *SPO12* [51] and *ESC8* [52] have only minor and indirect roles in *S. cerevisiae* meiosis. Overall, our results demonstrate very limited meiotic gene loss and the retention in *Sd. ludwigii* of the essential gene machinery for meiotic recombination.

Eight of the putative *Sd. ludwigii* meiotic proteins have little or no similarity to their *S. cerevisiae* homologs, and the identification of their genes was mostly based on synteny. These are *MEI4*, *NDJ1*, *PSY3*, *SPO16*, *REC104*, *POL4*, *IML3,* and *HED1* (Additional file 4: Table S3). Using similarity searches with either the *S. cerevisiae* or *Sd. ludwigii* protein sequences as queries, most of these genes could not be detected in the closely related *Hanseniaspora* species, which is consistent with extensive loss of genes involved in DNA repair, cell cycle regulation, and meiosis from this genus [53].

### Meiotic recombination components are required for *Sd. ludwigii* meiosis

**Fig. 4:**
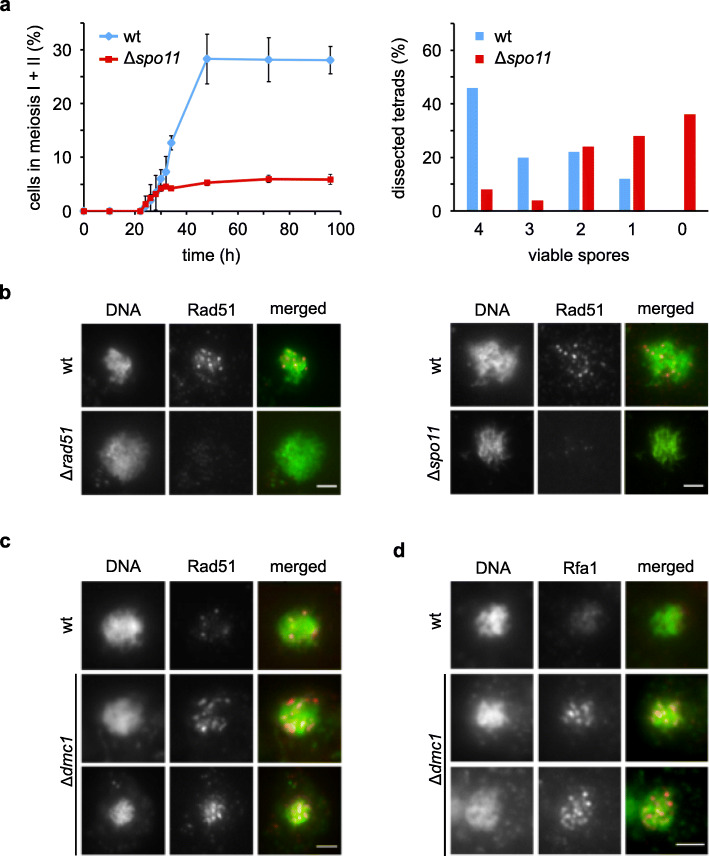
Core meiotic recombination components are required for normal meiosis in *Sd. ludwigii.* **a** Meiotic time-course analysis of a homozygous Δ*spo11 Sd. ludwigii* mutant in comparison to its wild type (left). After induction of meiosis, samples were withdrawn at the indicated time points and their cellular DNA content was stained with Hoechst 33258 to determine the fractions of binucleate (meiosis I) and tetranucleate (meiosis II) cells. Error bars: SD (*n* = 3 replicates for each strain). Spore viability was examined using tetrad dissection and viable colony counting (right). A total of 50 tetrads of each strain were analyzed. **b–d** Meiotic nuclear spreads of sporulating cells with the indicated genotypes (homozygous diploids). Cellular DNA was stained with Hoechst 33258. Immunostaining was performed using anti-Rad51 or anti-Rfa1 antibodies. Bars: 1 μm

The presence of a nearly intact meiotic gene machinery suggests that these genes are functional in *Sd. ludwigii*. This is further supported by our finding that deletion of *SPO11*, which in other organisms initiates meiotic recombination by generating DNA DSBs, led to significantly reduced sporulation and spore viability (Fig. 4a), similarly to *spo11* hypomorphs of *S. cerevisiae* [54, 55]. These results indicate an important function of this protein in meiosis. Since *S. cerevisiae* Δ*spo11* mutants also exhibit defects in chromosome synapsis [10], we also deleted *SAE2*, a regulator promoting the dsDNA endonuclease activity of Mre11 in the Mre11-Rad50-Xrs2 complex that operates downstream of Spo11 in DSB processing [56]. This led to a similar sporulation defect to that of the Δ*spo11* strain (Additional file 2: Fig. S3), suggesting a role for Spo11 in catalyzing DSB formation in *Sd. ludwigii* meiosis. In order to confirm directly the occurrence of Spo11-dependent DSBs, we investigated the meiotic localization of Rad51, a recombinase that is involved in DSB repair [8]. Using an anti-Rad51 antibody, we detected discrete foci in spreads of meiotic *Sd. ludwigii* chromosomes. No such foci were observed in Δ*rad51* or Δ*spo11* mutants, indicating the specificity of detection and the dependence of DSBs on Spo11 (Fig. 4b). We detected 5–12 foci per nucleus, which would be consistent with the formation of 1–2 Spo11-dependent DSBs per meiotic chromosome. Our results overall suggest that Spo11 is required for normal meiosis in *Sd. ludwigii*, through the generation of meiotic DNA DSBs.

We also performed Rad51 immunostaining in meiotic spreads of *Sd. ludwigii* cells deleted for *DMC1*, a ubiquitous meiosis-specific recombinase involved in DSB repair as well as in promoting interhomolog interactions. In this case, we observed elongated, filamentous foci (Fig. 4c), which could be consistent with the facts that in *S. cerevisiae* the filaments of both proteins often co-occupy the same ends of meiotic DSBs [57], and absence of Dmc1 leads to persistent Rad51 foci, which accumulate and become less punctate with time [58]. Another conserved single-stranded (ss) DNA-binding protein that is involved in DNA replication, repair and recombination, is Rfa1. We detected expanded Rfa1 foci in Δ*dmc1* meiotic spreads, whereas no foci were detected in wild-type spreads (Fig. 4d). This is congruent with the normally transient nature of ssDNA-binding activity of Rfa1, which is replaced by Dmc1, and the accumulation of ssDNA at DSBs in Δ*dmc1* cells, similarly to *S. cerevisiae* [59]. Overall, formation of meiotic DSBs and their recombinational repair, mediated by key meiotic components, are required for normal meiosis in *Sd. ludwigii*, consistently with what is known from *S. cerevisiae*.

### High-resolution analysis of meiotic segregation in *Sd. ludwigii*

**Fig. 5:**
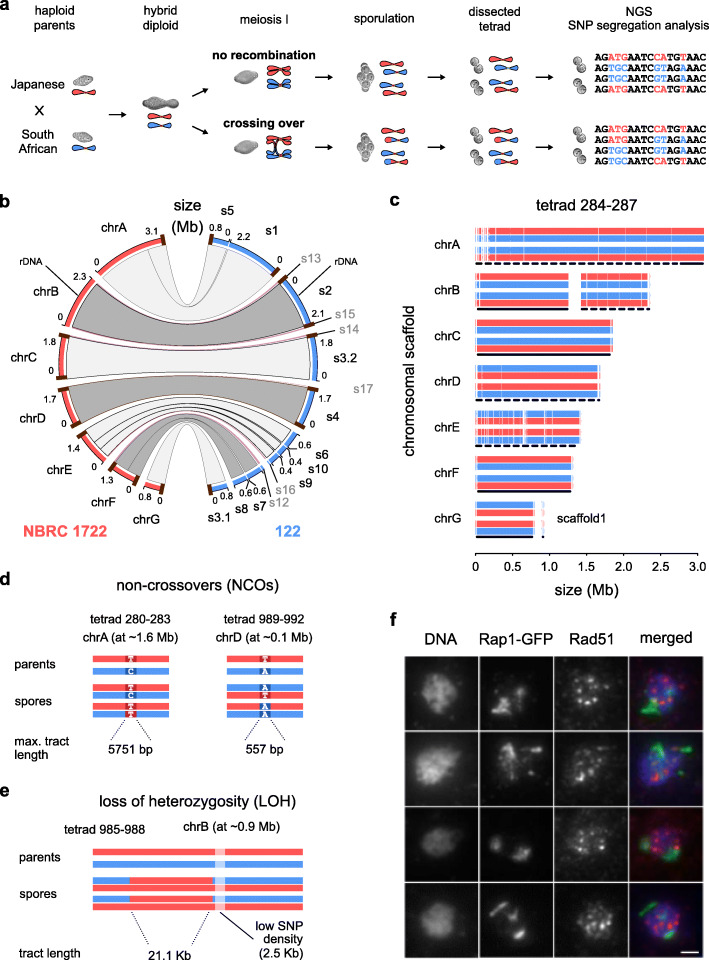
A high-resolution segregation analysis revealed the absence of crossing over in *Sd. ludwigii* meiosis. **a** Experimental setup (further details in Additional file 2: Fig. S4a). **b** Genomic comparison of the haploid parental strains. **c** Results of the genome-wide SNP segregation analysis for a representative tetrad. Each dimorphic position of the genome was assigned to one of the parental haplotypes (red for the reference strain, blue for spore 122). Genomic regions with high and low levels of sequence divergence are indicated with solid and dashed lines (underneath), respectively. **d** Two verified single-marker NCOs were detected by the SNP segregation analysis. **e** A single LOH event was detected in tetrad 985–988, in close proximity to another shorter region with unusually low SNP density. **f** Immunostaining of meiotic nuclear spreads of a *Sd. ludwigii* strain expressing Rap1-GFP using anti-Rad51 and anti-GFP antibodies. Bar: 1 μm

Our findings suggest that meiotic recombination might still be occurring in *Sd. ludwigii*, albeit with patterns that perhaps prevented detection by segregation analyses using only few markers. To address this, we performed a high-resolution genome-wide SNP segregation analysis of *Sd. ludwigii* meiosis (Fig. 5a; Additional file 2: Fig. S4a). We used as parents the reference strain and a spore that we isolated from a tetrad of a geographically distant strain (spore 122; Additional file 1: Table S1). Crosses between these two haploid strains frequently formed tetrads with 4 viable spores. Long-read sequencing and de novo assembly of the second parent’s genome did not reveal significant karyotypic differences that could prevent pairing of homologous chromosomes in this cross (Fig. 5b). Variant calling between the two strains resulted in a total of 199,356 high-quality SNPs, with an overall sequence divergence of 1.59%, while the mean and median distances between adjacent SNPs were 62 and 16 bp, respectively. Notably, heterogeneity was observed in SNP density across the genome, with the higher-heterozygosity genomic compartments (i.e., chrC, chrF, chrG, and parts of chrA and chrB; 44.6% of the genome) exhibiting on average 3.3% divergence (mean/median distance between SNPs: 38/16 bp), whereas the remaining, less divergent parts of the genome (i.e., chrD, chrE, and the remaining parts of chrA and chrB; 55.4% of the genome) displayed on average 0.2% sequence divergence, and 470/201 bp between SNPs (mean/median distance) (details per chromosome are provided in Additional file 2: Fig. S4b).

Sequencing of all spores from 5 full tetrads from this cross followed by analysis of the SNP segregation patterns revealed complete absence of meiotic COs (Fig. 5c). The same was observed when we extended our analysis to 2 tetrads from another diploid strain (originating from the wild-type isolate CBS 5929; Additional file 1: Table S1; Additional file 2: Fig. S4a; Fig. S4b). These results confirm the previous genetic evidence that suggested extreme rarity of meiotic crossing over in *Sd. ludwigii* [11, 12]. Among the 7 tetrads, we detected 2 independent single-marker NCOs (gene conversion tracts with 3:1 segregation patterns; Fig. 5d), which were validated by PCR amplification and sequencing. Their maximum tract lengths were 5.8 and 0.6 kb, respectively (calculated based on the distance between their closest flanking SNPs with 2:2 segregation patterns). This suggests that at least some of the meiotic DSBs are processed by interactions that use the homologous chromosome as repair template without, however, leading to CO formation. Finally, our analysis revealed a 21.1 kb-long loss-of-heterozygosity (LOH) tract (Fig. 5e), which could have resulted from a mitotic gene conversion event during diploid growth preceding sporulation. This LOH event occurred in close proximity to a 2.5 kb-long region of distinctly lower SNP density than that of the flanking chromosomal regions (Fig. 5e), indicating that another ancient event had occurred in the same region.

High sequence divergence between homologous chromosomes is known to lower the probability of recombination, perhaps especially of class I interfering COs, due to the mismatch repair system that rejects recombination intermediates and redirects them towards NCOs or sister-chromatid recombination [60, 61]. This could probably impede recombination in the high-diversity genomic regions, but the low-heterozygosity parts, which constitute more than half of the hybrid genome, should not be affected, since their divergence levels are substantially lower than those used in previous yeast studies [43, 45, 62, 63]. Despite the particularly high resolution of our analysis in the higher-diversity regions, we detected only 2 NCOs in total, which only leaves open the possibility of sister-chromatid repair.

Telomeric regions of chromosomes are highly repetitive (Additional file 2: Fig. S1b), and this could compromise the sensitivity of our method for the detection of COs in these regions [31, 43, 45, 62]. To investigate the possibility of crossing over in telomeric or telomere-proximal regions, we used meiotic chromosome spreads to compare the localization of GFP-tagged Rap1, a cytological marker of telomeres [64], and that of Rad51 foci as an indicator of meiotic DSBs [8]. If subtelomeric DSBs mediated chromosome interactions for meiosis I, we would expect to have at least one Rad51 dot per chromosome at or near a telomere. Since we only detect approx. 10 Rad51 foci per nucleus, we would expect to see more than half of the Rad51 dots colocalizing with Rap1. This was never the case (Fig. 5f) in 17 investigated nuclei. Rare colocalization of a single Rad51 dot with Rap1 could be expected to happen by chance, since Rap1 does not only localize to telomeres, but also to other chromosomal regions [65]. Therefore, initiation of recombination is not biased towards telomeres or adjacent regions in *Sd. ludwigii*, and the extreme suppression of crossing over appears to affect the entire genome.

### Search for signs of historical crossing over in *Sd. ludwigii*

The unusual suppression of homolog interactions in *Sd. ludwigii* meiosis motivated us to search for signs of recombination by comparing divergent strains of international origin. For this, we sequenced 10 available haploid and diploid strains (Additional file 5: Table S4), two of which were found to be aneuploids (2n + 1 trisomies) by a read coverage analysis (Additional file 2: Fig. S5a). Variant calling and comparison to our reference strain revealed variable sequence divergence (~ 18,000–308,000 SNPs) and heterozygosity levels in diploids (~ 1300–60,000 heterozygous SNPs) (Additional file 5: Table S4). Apart from the very divergent haploid strain PC99_R_1, sequence variation showed non-uniform patterns of distribution across the genome, being mostly restricted to particular chromosomes (spore 122) or, in most strains, to chromosomal segments (Additional file 2: Fig. S5b).

**Fig. 6:**
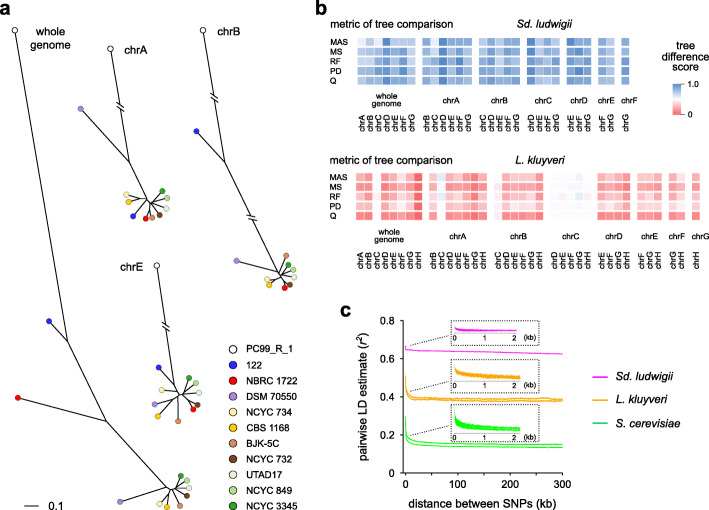
Crossing over has been absent or unusually rare in *Sd. ludwigii* over evolutionary time. **a** Dendrograms of *Sd. ludwigii* strains, constructed by neighbor-joining analysis of all SNPs genome-wide (“whole genome”) or SNPs of individual chromosomes. The dendrograms of the remaining chromosomes are shown in Additional file 2: Fig. S5c. **b** Scores of tree difference (5 topological, unrooted metrics) between all combinations of whole-genome SNPs and SNPs of individual chromosomes, for *Sd. ludwigii* and *L. kluyveri*. The analyzed *L. kluyveri* strains and their corresponding dendrograms are provided in Additional file 2: Fig. S5d. The results shown here are based on normalized distances to the average values of 1000 randomly generated tree pairs (uniform average method). MAS: unrooted maximum agreement subtree distance; MS: matching split distance; RF: Robinson-Foulds distance; PD: path difference distance; Q: Quartet distance. **c** Decay of LD as a function of physical distance between SNP marker pairs, for *Sd. ludwigii* in comparison to representative *L. kluyveri* and *S. cerevisiae* groups of strains. The moving averages of the LD estimate *r*^2^ values are plotted for all SNP marker pairs (genome-wide) of different physical distances. All data (no line smoothing) for the first 2 kb of distance are plotted in the insets

Phylogenetic analysis of all strains based on their genome-wide SNP content revealed the presence of a major cluster of 7 strains, and 4 more divergent ones (Fig. 6a). When we compared the dendrograms of individual chromosomes, we observed chromosome-dependent topologies and distances for particular strains (Fig. 6a, b; Additional file 2: Fig. S5c). Our analyses revealed that chromosomes of strains with unusually unstable topologies differ significantly in SNP density from their genomic average (Additional file 2: Fig. S5e). One possible explanation for these contrasting chromosome-specific signals could be that absence or rarity of meiotic recombination over an extended period of time has deprived chromosomes of the corresponding homogenizing effect, allowing them to accumulate SNPs independently from their homologs to a certain extent. We gained further support for this hypothesis by performing the same analysis in the yeast *Lachancea kluyveri*, a Saccharomycetaceae member with a similar genome size and chromosome number to *Sd. ludwigii*, but with demonstrated capability of meiotic recombination [45]. The analysis revealed very stable topologies in *L. kluyveri*, with all chromosomes yielding the same topology as the whole genome (Additional file 2: Fig. S5d, 5f; Fig. 6b).

Next, we performed a genome-wide linkage disequilibrium (LD) analysis in *Sd. ludwigii*, in comparison to *L. kluyveri* and *S. cerevisiae*, which are characterized by low and high meiotic recombination activity, respectively [45, 62]. Generation of LD decay curves, for which the LD estimate *r*^2^ for each pair of SNPs is plotted versus their physical distance, revealed striking differences between the species (Fig. 6c). The overall levels of LD were unusually high in *Sd. ludwigii*, intermediate in *L. kluyveri*, and lowest in *S. cerevisiae*. Furthermore, LD decayed extremely fast in *Sd. ludwigii*, where it reached its plateau within the first ~ 50 bp, in contrast to the more gradual decay of the 2 other species, which is typical of recombining organisms [66, 67]. Four of the *Sd. ludwigii* chromosomes exhibited even higher LD levels and faster decay than the genomic average (Additional file 2: Fig. S6a). To address the limitation of the small sample size in these analyses (due to the limited availability of wild-type *Sd. ludwigii* strains), we always compared groups of the same size (11 strains for all species), and we also compared multiple groups of randomly selected strains of *L. kluyveri* and *S. cerevisiae*. Curves of LD decay were quite stable and the observed differences between species were independent of the particular group considered (Additional file 2: Fig. S6b). These results are consistent with the hypothesis of absent or very rare meiotic recombination in *Sd. ludwigii* over evolutionary time, which would decrease the overall LD levels and decelerate its decay.

### Mutation rate and genomic evolution in *Sd. ludwigii*

A hypothetically elevated mutation rate could be a compensatory solution for the generation of genetic diversity in *Sd. ludwigii* in the absence of meiotic recombination, or even serve as a driver of achiasmate meiosis [27]. Furthermore, we reasoned that a significant AT bias of spontaneous mutations could explain the particularly low GC content of the *Sd. ludwigii* genome (Additional file 2: Fig. S2a). We investigated these hypotheses directly by a mutation accumulation analysis in 3 founder *Sd. ludwigii* strains: our haploid reference strain, a corresponding homozygous diploid strain, and the hybrid diploid strain used in our SNP segregation experiment (Table 1; Additional file 1: Table S1). We adopted a single-cell population bottlenecking regime of growth on agar plates to evolve 60 independent mutation accumulation lines for ~ 2000 generations each. Such an experimental setup ensures fixation of the majority of spontaneous mutations and minimizes elimination of non-lethal deleterious mutations due to selection [68]. Sequencing and genome-wide analysis revealed a total of 186 line-exclusive single-nucleotide mutations (SNMs) (Additional file 6: Table S5). This corresponds to an overall base-substitutional mutation rate (*μ*_bs_) of 5.70 × 10^−11^–1.20 × 10^−10^ mutations per base per generation, for the 3 strain genealogies (Table 1). These rates, which are in line with the rate of another recently analyzed *Sd. ludwigii* diploid strain [69] (7.3 × 10^−11^), fall within the range of unicellular eukaryotes, and they are even slightly lower than those of other yeast species [69, 70]. Therefore, mutation rate in *Sd. ludwigii* is not unusually elevated. Similarly, the overall transition-to-transversion (Ts:Tv) ratio (1.21 in *Sd. ludwigii*) is comparable to the values reported for *S. cerevisiae* and expected for organisms with no cytosine methylation [70].

**Table 1:** Mutation accumulation analysis. The base-substitutional mutation rate (*μ*_bs_), its Poisson 95% confidence interval (CI), the AT bias (weighted by genomic nucleotide composition), and the transition:transversion ratio (Ts:Tv) of spontaneous single-nucleotide mutations are provided for each strain genealogy

Strain	# generations (average)	# mutations (# lines)	***μ***_**bs**_ (/site/generation)	Poisson 95% CI	AT bias	Mutation-driven equilibrium GC content	Observed GC content	Ts:Tv ratio
Haploid (NBRC 1722)	2037	85 (30)	1.20 × 10^−10^	9.62 × 10^−11^–1.49 × 10^−10^	2.92	0.255	0.296	1.18
Isogenic diploid (YLFP17-4)	1920	48 (19)	5.70 × 10^−11^	4.20 × 10^−11^–7.55 × 10^−11^	5.85	0.146	0.296	1.53
Hybrid diploid (YLFP188-1)	2016	53 (11)	9.55 × 10^−11^	7.16 × 10^−11^–1.25 × 10^−10^	2.63	0.276	0.296	0.96

The *Sd. ludwigii* genome has a remarkably low GC content. We determined that, similarly to the majority of eukaryotes, *Sd. ludwigii* has a strong AT bias of spontaneous mutations, with SNMs that convert GC to AT occurring on average 3.6 times more frequently than SNMs in the opposite direction (Table 1). Based on this, the theoretical equilibrium GC content of the *Sd. ludwigii* genome should be 23.3%, if it were determined by spontaneous mutations alone. In these calculations, we excluded repeat regions (~ 7% of the genome), since they are difficult to analyze and are associated with a high indel formation frequency [71]. The observed genomic GC content of the analyzed genomic fraction was 29.6%, which cannot be explained by the effect of spontaneous mutations only. Therefore, balancing forces in the opposite direction, such as selection on GC or GC-biased gene conversion [70], are probably also present in *Sd. ludwigii*.

### Mitotic LOH in *Sd. ludwigii*

The striking rarity of homolog interactions in *Sd. ludwigii* meiosis prompted us to investigate the levels of mitotic homologous recombination, to understand whether the observed strong suppression is specific to meiosis. Further analysis of the 11 mutation accumulation lines of the hybrid diploid strain (Table 1) revealed a total of 22 LOH events (Additional file 7: Table S6), half of which occurred in chrA. Chromosome A is the largest chromosome (representing ~ 25% of the *Sd. ludwigii* genome). Whether this relative enrichment of events in this chromosome explains its lower SNP density compared to other chromosomes (Additional file 2: Fig. S4b) is unclear. Similarly, non-uniform distributions of LOH events have been observed in *S. cerevisiae* [72, 73]. Among the detected events, we identified one long terminal event (in chrC; ~ 183 kb), which could be attributed to either a mitotic crossover or a break-induced replication (BIR) event [74]. The remaining events were shorter interstitial LOH regions with a maximum length of 12.5 kb (Additional file 7: Table S6). These may be associated with mitotic crossing over or gene conversion [72, 73, 75]. The total genomic rate of LOH events was 9.9 × 10^−4^ events per generation, which is moderately lower than the 3–10 times higher frequencies reported for *S. cerevisiae* [72, 73]. Therefore, the core machinery for repair of spontaneous DSBs during the vegetative life phase of *Sd. ludwigii* is present and functional. The extreme suppression of recombination is, therefore, limited to meiosis.

## Discussion

Repair of meiotic DSBs during prophase I can result in chiasmata that promote accurate segregation of homologous chromosomes and enable genetic exchanges between them [8, 9, 13]. Meiotic cells preferentially rely on homologous recombination, which can repair a subset of these DSBs to form COs, whereas the remainder lead to NCOs or use the sister chromatid as repair template [76, 77]. The pathway choice decision is controlled by multiple cytological factors [78] and can be subject to evolutionary selection. Our study revealed that Spo11-mediated introduction of DSBs is required for normal meiosis in *Sd. ludwigii*. Given that immunostaining of Rad51 for visualizing DSBs may underestimate their number [58], we hypothesize that the observed 5–12 Rad51 foci correspond to at least one to two DSBs per chromosome in *Sd. ludwigii* meiosis. The demonstrated involvement of Dmc1 in DSB processing further suggests that at least a fraction of these DSBs are processed through homolog-templated repair pathways [8, 9, 13], but with a strong bias towards NCOs as suggested by the absence of COs and the detection of two NCOs in our meiotic SNP segregation analysis. Since NCOs typically involve short gene conversion tracts (usually 1–2 kb in *S. cerevisiae* [62, 79]), more such events might have occurred in our analyzed tetrads below the resolution limit of the analysis. The total absence of crossing over throughout the genome corroborates the hypothesis of Yamazaki and coworkers, who proposed that crossing over as an obligatory feature of meiosis I might be absent from *Sd. ludwigii* meiosis [11, 12]. Considerable involvement of NHEJ in the repair of meiotic DSBs is unlikely, given that NHEJ is generally suppressed during meiosis [80, 81], and *Sd. ludwigii* misses four components of the major NHEJ pathway.

**Fig. 7:**
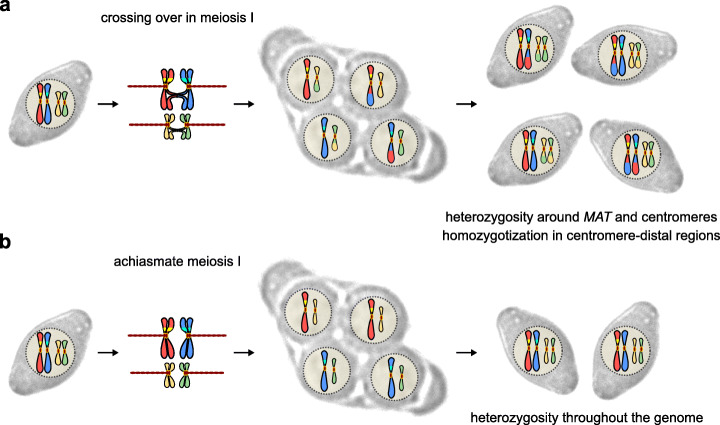
High rates of intratetrad mating coupled with suppression of meiotic recombination maximize preservation of heterozygosity. **a** Non-sister intratetrad mating with crossing over in meiosis I. The mating-type locus is linked to the centromere of its chromosome (which is often observed in species that engage in intratetrad mating, e.g., *S. cerevisiae* and *Sd. ludwigii*). **b** Non-sister intratetrad mating with achiasmate meiosis I, which normally happens in *Sd. ludwigii*

The level of heterozygosity present in individuals and, therefore, the extent of genetic diversity in populations are largely dependent on mating strategies. Frequent intratetrad mating, in particular, preserves high levels of heterozygosity in parts of the genome (Fig. 7a), ensures efficient purging of deleterious mutations, and provides fitness advantages [24, 26, 27, 82]. Suppression of recombination in organisms that often engage in intratetrad mating could be beneficial for their evolution by extending the preservation of heterozygosity to larger parts of their genomes [25] (Fig. 7b). Our findings support the hypothesis that achiasmate meiosis might have evolved in the *Sd. ludwigii* lineage through mutual selection between suppression of meiotic recombination and frequent intratetrad mating, which is its predominant mating behavior [34, 83]. Low recombination rates could have arisen through the accidental loss of an important meiotic gene, e.g., *MER1* [49], which could have spread to fixation possibly due to genetic hitchhiking by suppressing recombination along the entire chromosomes. The need to overcome the negative consequences of chromosome non-disjunction due to the loss of crossing over could have driven the selection of mechanisms or behaviors that alleviate the problem of inviable spores due to chromosomal mis-segregation. This organism appears to be avoiding this risk by enforcing intratetrad mating in its life cycle through the development of robust interspore bridges that keep non-sister spores tightly linked together in its asci [34, 83, 84]. Mating with non-sister meiotic siblings would restore a full diploid chromosome complement (Fig. 7b) even in the case of aneuploid individual spores, which are still likely capable of mating [27, 85]. Absence of recombination would prevent the exchange of mating types between non-sister chromatids in meiosis I and, therefore, the requirement for opposite mating types would lead to full reconstitution of the parental diploid genomic content upon mating. In this scenario, the duet of suppressed recombination and frequent intratetrad mating could have persisted because of the efficient preservation of heterozygosity and the fitness advantages that this could offer, according to previous research [25–27].

Further support for our hypothesis that loss of *MER1* could have mediated the extreme suppression of meiotic crossing over may be provided by the finding that one of its splicing targets, i.e., *SPO22* (*ZIP4*), appears to retain an intron in *Sd. ludwigii*. This gene codes for a synapsis initiation complex component that is required for proper SC formation, chromosome synapsis, and class I (interfering) COs in *S. cerevisiae* [86]. Although Zip4 homologs of other organisms (*A. thaliana*, rice, and mouse) have only minor roles in the completion of synapsis, the important Zip4 function in interfering crossing over appears conserved [87–89]. Therefore, potential dysregulation of Zip4 due to the loss of Mer1 from *Sd. ludwigii* could lower its crossing over levels, or even cause their absence in case the class II CO pathway is not functional in this species. In accordance with this hypothesis, our preliminary investigations of *Sd. ludwigii* meiotic proteins revealed the following: (i) markedly less elongated SC structures than in *S. cerevisiae*, possibly indicating defects in Zip1 polymerization, which in *S. cerevisiae* depends on Spo22/Zip4 [86], and (ii) while deletion of *msh4* (class I CO formation [90]) led to remarkably defective meiosis, absence of Mms4 (class II crossing over [91]) had no effect on spore viability, which would be consistent with the idea that the class II pathway may be unimportant in *Sd. ludwigii* meiosis.

Another possible strategy that is often observed in *Sd. ludwigii* strains [92] is the completion of meiosis with only one division, which leads to two-spore asci with diploid spores. Secondary loss of *SPO12*, which is absent from *Sd. ludwigii*, might have helped to achieve this and prevent a meiotic arrest in cell cycle progression, since natural *S. cerevisiae* strains lacking *SPO12* and *SPO13* exhibit the same behavior [93].

The establishment of a lifestyle that successfully copes with the lack of meiotic recombination does not exclude the possibility of rare outbreeding [84] that would occasionally permit intermixing of chromosomes, as was observed in our population analysis (Fig. 6a; Additional file 2: Fig. S6c). In addition, the species seems not to have completely abolished its ability to rarely recombine its genome [11, 12], which explains the preservation of the corresponding gene arsenal, and the partial rescuing of its Mer1 regulon. Those processes would require the maintenance of chromosomal structural stability, without which the accumulation of gross rearrangements in homologs could hinder synapsis in meiosis I and preclude recombination. The loss of the classical NHEJ pathway from the *Sd. ludwigii* lineage (Fig. 3) could contribute to the preservation of genomic collinearity between its intra-specific lineages. Evidence from the older marker segregation studies [11, 12] suggested, indeed, that meiotic crossing over could still occur in *Sd. ludwigii*, but at extremely low frequencies (i.e., in 0.9% of their analyzed tetrads). Even if one assumes that the low resolution of their experiments led to a significant underestimation of that frequency, our higher-resolution data and the comparison with other yeasts clearly demonstrate that COs are extremely suppressed in *Sd. ludwigii* meiosis: analysis of its 7 tetrads yielded no COs, in comparison to ~ 630 expected COs in *S. cerevisiae* [62], ~ 315 in *S. pombe* [94], ~ 175 in *K. phaffii* [43], and ~ 125 in *L. kluyveri* [45].

Recombination, genomic GC content, and mutation rate are thought to be linked, although the causality of their relationships is still not fully understood [70, 95]. We performed a mutation accumulation analysis to investigate the reason for the particularly low genomic GC level in *Sd. ludwigii*. Its mutation rate falls within the normal range of related eukaryotes [70]. On the other hand, a strong AT bias of spontaneous mutations—similarly to many other organisms [70, 96]—in conjunction with the presumably low relevance of GC-biased gene conversion [68, 70] due to the rarity of recombination, provides an explanation for the unusually low GC content of its genome. Balancing forces in the opposite direction, such as selection on GC [97] and temporary or conditional changes of the mutation rate and/or bias [98], must also act, given that the expected equilibrium genomic GC level would be 6.3% lower than the actual one if it were determined solely by the mutational load.

Loss of heterozygosity has been suggested to offer rapid evolutionary solutions for phenotypic diversification and adaptation, by exposing recessive beneficial alleles or alleviating negative epistasis of alleles in the heterozygous state [73, 99, 100]. Such adaptive flexibility could be very important for *Sd. ludwigii*, which appears to suppress recombination of its genome. Our results indicate that LOH events, often resulting from mitotic COs or NCOs, occur in *Sd. ludwigii* at frequencies that are significantly higher than the base-substitution mutation rate. Suppression of recombination in this species is, therefore, limited to meiosis, and LOH could be an important mechanism for its adaptive potential.

The unique meiotic behavior of *Sd. ludwigii* renders the functional dissection of its meiotic mechanisms and their regulation interesting goals of future research. Investigation of ZMM proteins that provide links between the SC, which facilitates segregation of meiotic chromosomes in many organisms [9], and repair of meiotic DSBs [101, 102], as well as characterization of components that regulate homolog bias and pathway choice [9, 78, 103], could provide useful insights. Furthermore, an interesting subject could be the fast evolving meiotic proteins that were detected in *Sd. ludwigii*, such as Ndj1 and Spo16 that are involved in the regulation of CO formation and distribution in *S. cerevisiae* [104, 105]. However, further functional investigations would require the development of *Sd. ludwigii* strains with higher sporulation rates and better synchronization of meiosis I than the currently available ones.

## Conclusions

Our study demonstrates that the high rate of intratetrad mating in the yeast species *Saccharomycodes ludwigii* is coupled with extreme suppression of meiotic crossing over throughout its genome and that this behavior has persisted over evolutionary time. We propose that this combination ensures adaptive genome evolution in a trajectory that requires only minimal levels of meiotic recombination. Therefore, *Sd. ludwigii* provides a new paradigm and an excellent natural forum, in which to study experimentally the evolution of recombination rates and their influence on genome evolution.

## Methods

### Yeast strains—strain construction

All strains used in this study are listed in Additional file 1: Table S1. They were incubated at 30 °C (with shaking of liquid cultures at 230 rpm) for vegetative growth or at 23 °C for sporulation. All plasmids are provided in Additional file 8: Table S7 [106–109]. The *E. coli* strain DH5α was used for their maintenance and propagation. All DNA oligonucleotides used for plasmid/strain construction are listed in Additional file 9: Table S8. Gene deletion and tagging were performed using the PCR targeting method [108]. A lithium acetate (LiAc) and heat shock-based protocol was optimized and used to transform *Sd. ludwigii*. Detailed methods for mating, sporulation, tetrad dissection, transformation, and strain construction, as well as for pulsed-field gel electrophoresis (PFGE), transmission electron microscopy (TEM), and ploidy determination using flow cytometry, can be found in the Additional file 10: Supplementary detailed methods [11, 12, 110–143].

### Immunostaining of meiotic spreads

Spreads of *Sd. ludwigii* meiotic nuclei were prepared from sporulating cells in meiotic time courses using the procedure previously described for *S. cerevisiae* [144] with some modifications. The detailed protocol is provided in the Additional file 10: Supplementary detailed methods. The primary antibodies used were as follows: rabbit anti-*Sc*Rfa1 (gift from E. Mancera), sheep anti-GFP (generated in our lab) at 1:500 dilution each, and rabbit anti-*Sc*Rad51 (gift from A. Shinohara) at dilution 1:1000. The latter antibody [145] was used for detection of discrete Rad51 foci, while the anti-GFP antibody was used for localization studies of GFP-tagged Rap1 in spreads of meiotic nuclei. The secondary antibodies used were as follows: Alexa Fluor 488-conjugated donkey anti-rabbit IgG (Dianova), Cy2-conjugated donkey anti-sheep IgG (Dianova) and Cy3-conjugated donkey anti-rabbit IgG (Dianova), at 1:500 dilution each.

### Mutation accumulation experiment

Three *Sd. ludwigii* strains were used for the mutation accumulation study: the haploid reference strain NBRC 1722, the nearly isogenic diploid strain YLFP17-4 (homozygous diploid with the reference strain’s background), and the hybrid diploid strain YLFP188-1 (heterozygous diploid) (Additional file 1: Table S1). Clonal stocks of these parental strains were streaked on YPD plates, and randomly selected single colonies of each one served as founders of independent mutation accumulation lines (MALs). Spontaneous mutations were accumulated in 30, 19, and 11 MALs of the haploid, the homozygous and the heterozygous diploid strain, respectively, while they were growing under favorable conditions (YPD medium, at 30 °C). Each MAL was passed through a single-cell population bottleneck every 48 h by picking a random single colony and streaking it to single colonies on fresh YPD plates again. The plates were pre-marked with a target, and the single colony closest to the target was picked for each cycle, to ensure random colony selection. The experiment was performed for a total of 96–97 cycles, which corresponds to 2037 generations for the haploid, 1920 generations for the homozygous diploid, and 2016 generations for the heterozygous diploid strain (each cycle corresponds to 20 generations for the homozygous diploid strain or 21 for the two other strains, as determined by resuspending 10 independent colonies of each one in water and counting cells using a Neubauer-improved hemocytometer, at the beginning of the experiment).

### Total DNA extraction from *Sd. ludwigii*

Liquid *Sd. ludwigii* cultures in YPD medium (early stationary phase) were processed for DNA extraction using the Genomic-tip 20/G (mini prep, starting with approx. 10^8^ cells) or 100/G (midi prep, 10^9^ cells) kit (QIAGEN), according to the manufacturer’s recommendations. An additional washing step with buffer QC was performed before the final elution step. To recover DNA following isopropanol precipitation, spooling with a pipette tip was performed. A washing step was performed using 500 μl of ice-cold 70% ethanol. Following centrifugation (13,000 rpm for 10 min at 4 °C), ethanol was completely removed, the sample was air-dried at room temperature for 10 min, and DNA was then resuspended in 100 μl (mini prep) or 200 μl (midi prep) TE buffer (pH 8.0). To further improve the quality of the isolated DNA, 100 μl of each DNA sample were further processed using the DNeasy PowerClean Pro Cleanup kit (QIAGEN), according to the manufacturer’s instructions. Total DNA was finally eluted in 50 μl of elution buffer (10 mM Tris-HCl, pH 8.0) and stored at − 20 or − 80 °C. Alternatively, total DNA was isolated from *Sd. ludwigii* using the phenol-chloroform extraction-based protocol that is provided in the Additional file 10: Supplementary detailed methods.

### Next-generation sequencing (NGS)

#### Illumina sequencing

For sequencing on an in-house Illumina NextSeq 550 instrument, libraries were prepared using the NEBNext Ultra II FS DNA Library Prep Kit for Illumina (New England Biolabs), according to the manufacturer’s recommendations. Enzymatic fragmentation of DNA was controlled to generate fragment size distributions in the range of 300–500 bp. For PCR enrichment of adaptor-ligated DNA, six or seven amplification cycles were used for samples with higher and lower starting amounts of DNA, respectively. Multiple DNA samples were barcoded with unique dual indices using the NEBNext Multiplex Oligos for Illumina (96 Unique Dual Index Primer Pairs) (New England Biolabs) and pooled at equimolar concentrations. The NEBNext Library Quant Kit for Illumina (New England Biolabs) was used for the qPCR-based quantification of the pooled library, which was then loaded on a high-output flow cell (NextSeq 500/550 High Output Kit v2.1, 300 cycles; Illumina) for sequencing in paired-end mode (2 × 150 bp).

Alternatively, Illumina sequencing was performed on MiSeq and HiSeq 2000 instruments, at the EMBL Genomics Core Facility (Heidelberg, Germany), in paired-end mode (2 × 150 and 2 × 100 bp for MiSeq and HiSeq systems, respectively). Sequencing libraries were prepared by the Deep Sequencing Core Facility (University of Heidelberg, Germany). Briefly, mechanical shearing using a Covaris ultrasonicator was used for DNA fragmentation to a size range of 300–500 bp. The NEBNext Ultra II DNA Library Prep Kit for Illumina (New England Biolabs) was used for library preparation, and eight PCR cycles were performed for amplification of adaptor-ligated DNA.

#### Pacific Biosciences SMRT (single-molecule real-time) sequencing (PacBio)

For the generation of the de novo genome assembly of our reference *Sd. ludwigii* strain, we used PacBio sequencing, which was performed at GATC Biotech AG (now Eurofins Genomics). Briefly, library preparation involved DNA fragmentation, size selection (using a BluePippin instrument; Biozym Scientific), DNA end repair and adaptor ligation, and annealing of the primer and the polymerase. The PacBio library was then sequenced on 2 SMRT cells in a PacBio RS II instrument (run mode: 240 min. movie) using the P6-C4 chemistry. This yielded a total of 2.52 Gb of pass-filter sequence data, which corresponds to an average genomic sequencing depth of approx. 200×.

#### Oxford Nanopore Technologies sequencing (ONT)

Generation of genome assemblies of the other *Sd. ludwigii* strains was based on ONT sequencing on a MinION sequencer. The Ligation Sequencing Kit (for 1D experiments; SQK-LSK109) was used for library construction, starting from 1 μg of total DNA of each sample, and different samples were barcoded using the Native Barcoding Expansion 1-12 (PCR-free) kit (both kits were purchased from Oxford Nanopore Technologies) and pooled for multiplexed sequencing (according to the manufacturer’s instructions). No fragmentation of the input DNA was performed. The sequencing libraries were loaded on R9.4.1 flow cells and sequenced on a MinION device. Each strain was sequenced until the desired amount of sequencing data was gathered, corresponding to average genomic sequencing depths of approx. 30–40×.

### Genome assembly

The de novo genome assembly of the reference *Sd. ludwigii* strain NBRC 1722 was based on high-coverage PacBio sequencing data. A total of 193,117 reads (average genomic sequencing depth of approx. 200×) with mean length 13,041 bp and mean *q*-score 0.827 passed the filter (SMRT Portal, Pacific Biosciences). The Celera Assembler [146] and the Quiver [147] tool in the HGAP.3 pipeline (Hierarchical Genome Assembly Process) within SMRT Analysis v2.3.0 (Pacific Biosciences) were used for genome assembly and consensus polishing, respectively. A part of the mitochondrial genome was identified using BLAST+ v2.8.0 [120] and removed from the nuclear assembly. Subsequently, we used an Illumina MiSeq 2 × 150-bp paired-end read dataset generated from the same DNA sample for further polishing of the assembly using Pilon [148] (version 1.22), to generate the final assembly version (with an N50 value of 1,848,403 bp).

The de novo genome assemblies of strains 122 and NBRC 1723 were produced using ONT sequencing. We generated 93,607 filter-passing reads (average genomic sequencing depth of approx. 33×) with an N50 read length of 6.89 kb and a mean *q*-score of 13.242 for strain 122, and 102,478 reads (average genomic depth of approx. 42×) with an N50 read length of 7.76 kb and a mean *q*-score of 13.586 for strain NBRC 1723. The base-caller Guppy (v2.3.5; Oxford Nanopore Technologies) with the high-accuracy model was used for basecalling, and Porechop (v0.2.4; https://github.com/rrwick/Porechop) was used for demultiplexing of barcoded reads and trimming of ONT adaptors. Genome assemblies were then constructed using SMARTdenovo (https://github.com/ruanjue/smartdenovo) with parameters “-c 1 -k 14 -J 500 -e zmo”. The assembly of strain 122 was polished using an Illumina MiSeq 2 × 150-bp paired-end sequencing dataset of the same strain and Pilon [148] (v1.23). Both assemblies were scaffolded further using ONT long-read information with SSPACE-LongRead [149] (v1-1) and, in the case of strain 122, gaps were closed using GapFiller [150] (v1-10) and the Illumina reads. The NBRC 1723 assembly was corrected by mapping the raw ONT reads using minimap2 [151] (2.17) and then using Racon [152] (v1.3.3). The final assemblies were generated after a second round of scaffolding using SSPACE-LongRead (v1-1), followed by polishing using Pilon (v1.23) for strain 122 or Nanopolish [153] (v0.11.0) for strain NBRC 1723.

### Gene annotation and analysis of meiotic gene content

For the generation of a comprehensive *Sd. ludwigii* gene annotation dataset, we used the reference genome sequence and combined the results from different tools and pipelines, followed by extensive manual curation. The methods and tools used are described in detail in the Additional file 10: Supplementary detailed methods. All *S. cerevisiae* genes that are known to be involved in meiosis and recombination were retrieved from the Saccharomyces Genome Database (SGD, https://www.yeastgenome.org) by searching for genes with relevant GO terms. These searches retrieved a total of 284 genes, which were then used as queries for the identification of homologs in the *Sd. ludwigii* gene annotation dataset using BLAST+ analyses. For all genes that remained undetected after similarity searches, we performed detailed gene synteny comparisons with annotated Saccharomycetaceae species that are included in the Yeast Gene Order Browser (YGOB) database (v7; http://ygob.ucd.ie). Further details are provided in the Additional file 10: Supplementary detailed methods.

### Meiotic segregation analysis

Reads were mapped to the *Sd. ludwigii* reference genome (strain NBRC 1722), which was previously masked with RepeatMasker (v4.0.7, default parameters; http://www.repeatmasker.org), using bwa mem [154] (v0.7.17). Resulting bam files were sorted and indexed using SAMtools [155] (v1.9). Duplicate reads were marked and sample names were assigned using Picard (v2.18.14; https://broadinstitute.github.io/picard/). The GATK pipeline [156] (v3.7.0) was used to realign remaining reads. Variants were then called using GATK UnifiedGenotyper. Calling was performed simultaneously for all spores from the same tetrad or all lines from the same background.

For the single-nucleotide polymorphism (SNP) segregation analysis of the hybrid cross, SNPs called from the respective reads mapping were first filtered (bcftools view; v1.9, https://github.com/samtools/bcftools) in order to define a set of high-confidence discriminant markers. Only positions with a single alternate allele, supported by at least 10 reads across both parents and with > 90% of the reads covering either the reference or alternate allele, were selected. For each tetrad of the cross, SNPs located at the aforementioned marker positions were extracted, and the parental origin was assigned based on SNP correspondence between parents and spores at those positions. The result was formatted as a Seg file and used as input of the CrossOver pipeline (ReCombine suite) [157], using default parameters and modified chromosome sizes/coordinates to match the reference genome of *Sd. ludwigii*. The reported events were individually validated by visual inspection using the Integrative Genomics Viewer (IGV). The same method was applied for the SNP segregation analysis of the South African cross, except that the minimum amount of reads supporting a marker position was lowered to 3 for each parent, as the coverage was reduced for one of the parents.

### Mutation accumulation analysis

Filters were applied to the SNPs called from the mutation accumulation lines to identify SNPs that were present only in a single line, as we expected these events to be line-exclusive. First, only positions covered by more than 10 reads in each sample with a single alternate allele were kept. Then, filters based on the numbers of lines per background and the type of conversion event expected to occur in a given background (homozygous to homozygous, homozygous to heterozygous, heterozygous to homozygous) were applied. Heterozygous SNPs were only retained if their allele balance was between 0.4 and 0.6. In the case of heterozygous-to-homozygous substitution, likely LOH events were filtered out to prevent false-positive SNP calls. The base-substitutional mutation rate (*μ*_bs_) of each strain genealogy was calculated as the ratio of the total number of line-exclusive novel mutations divided by the size of the analyzed fraction of the genome (after masking), the total number of generations, and the number of respective lines. The 95% Poisson confidence intervals of mutation rates were computed as in Long et al. [158], and the mutation bias (corrected for the genomic GC content) as well as the theoretical equilibrium GC level (under mutation pressure alone) was calculated as in Krasovec et al. [159].

### Population analyses

A total of 558,629 biallelic segregating sites were used to construct the neighbor-joining trees using the R packages ape and SNPrelate. The .gvcf matrices (of the whole genome or individual chromosomes) were converted into .gds files. Individual dissimilarities were estimated using the snpgdsDiss function, and the bionj algorithm was run on the distance matrices. Tree comparison was performed using the Visual TreeCmp package [160] (web application; v2.0.76), using 7 unrooted metrics (topological or weighted). For normalization of distances, the results of the topological metrics were compared to the average values of 1000 randomly generated tree pairs (uniform average method). The software PopLDdecay [161] (v3.41) was used for the calculation of the LD decay data, which were then smoothed (moving average method) and plotted using R.

### Other bioinformatic analyses

Our methods and tools used for genomic comparisons, determination of structural variation, and identification of repetitive regions are provided in the Additional file 10: Supplementary detailed methods.

## Supplementary Information

**Additional file 1: Table S1.** List of strains.

**Additional file 2: Figs. S1-S6**.

**Additional file 3: Table S2.** Genome assemblies of yeasts and filamentous fungi.

**Additional file 4: Table S3.** Genes involved in meiosis/meiotic recombination and their presence/absence in *Sd. ludwigii* and other yeasts.

**Additional file 5: Table S4.** Population analyses: read mapping statistics.

**Additional file 6: Table S5.** Novel mutations in the mutation accumulation experiment.

**Additional file 7: Table S6.** LOH events in the mutation accumulation experiment.

**Additional file 8: Table S7.** List of plasmids.

**Additional file 9: Table S8.** List of PCR primers.

**Additional file 10.** Supplementary detailed methods.

**Additional file 11.** review history

## Data Availability

All sequencing datasets and genome assemblies generated and analyzed in this work are available at the NCBI databases, organized in BioProjects with accession numbers PRJNA28063 [162] (genomics and meiotic segregation analyses), PRJNA578491 [163] (mutation accumulation analysis), and PRJNA639224 [164] (population analyses). Correspondence and requests for materials should be addressed to M.K. (m.knop@zmbh.uni-heidelberg.de).
