# Supplementary material for: Sex without crossing over in the yeast Saccharomycodes ludwigii
Source: Genome Biol. 2021 Nov 3;22:303. doi: 10.1186/s13059-021-02521-w (PMC8567612; doi:10.1186/s13059-021-02521-w)
Supplement: Supplementary file 1 — Additional file 1: Table S1. List of strains. [file 13059_2021_2521_MOESM1_ESM.pdf]

Table S1 List of yeast strains generated and used in this study

Strain		Parental background	Genotype	Ploidy	Mating partners (for diploids)	Source/ Reference
ID[1]	Name					
**Parental strains (used for genome sequencing, crosses and/or strain construction)**						
Saccharomycodes ludwigii						
Sc-9750	NBRC 1721[2]		wild-type isolate (origin: Japan)	diploid		[37]
Sc-9752/9753	NBRC 1722[3]		*MATα*, meiotic descendant of wild-type isolate NBRC 1721	haploid		[35]
Sc-9754	NBRC 1723[4]		*MATa*, meiotic descendant of wild-type isolate NBRC 1721	haploid		[35]
Sdl-75	CBS 5929[5]		wild-type isolate (origin: South Africa)	diploid		
**Strains used for functional analyses of meiotic genes**						
Sdl-206	YLFP10-1	Sc-9753	*MATα Δura3::hphNT1*	haploid		this study
Sdl-307	YLFP15-1	Sdl-206	*Δmatα::URA3-kanMX4 Δura3::hphNT1*	haploid		this study
Sdl-334	YLFP16-5	Sdl-307	*MATa Δura3::hphNT1*	haploid		this study
Sdl-339	YLFP18-1	Sdl-334	*MATa* (isogenic to NBRC 1722)	haploid		this study
Sdl-336	YLFP17-1		*MATa/MATα URA3/Δura3::hphNT1*	diploid	Sc-9753 **x** Sdl-334	this study
Sdl-502	YLFP17-4		*MATa/MATα*	diploid	Sc-9753 **x** Sdl-339	this study
Sdl-452	YLFP37-1	Sdl-339	*MATa Δspo11::kanMX4*	haploid		this study
Sdl-361	YLFP23-1	Sc-9753	*MATα Δspo11::hphNT1*	haploid		this study
Sdl-539	YLFP58-1		*MATa/MATα Δspo11::kanMX4/Δspo11::hphNT1*	diploid	Sdl-452 **x** Sdl-361	this study
Sdl-878	YLFP157-1	Sc-9753	*MATα Δrad51::kanMX4*	haploid		this study
Sdl-882	YLFP158-1	Sdl-339	*MATa Δrad51::kanMX4*	haploid		this study
Sdl-886	YLFP159-1		*MATa/MATα Δrad51::kanMX4/Δrad51::kanMX4*	diploid	Sdl-878 **x** Sdl-882	this study
Sdl-926	YLFP174-1	Sc-9753	*MATα Δdmc1::kanMX4*	haploid		this study
Sdl-930	YLFP175-1	Sdl-339	*MATa Δdmc1::kanMX4*	haploid		this study
Sdl-958	YLFP182-1		*MATa/MATα Δdmc1::kanMX4/Δdmc1::kanMX4*	diploid	Sdl-926 **x** Sdl-930	this study
Sdl-728	YLFP99-1	Sc-9753	*MATα RAP1-GFP-kanMX6*	haploid		this study
Sdl-736	YLFP101-1	Sdl-339	*MATa RAP1-GFP-kanMX6*	haploid		this study
Sdl-764	YLFP108-1		*MATa/MATα RAP1-GFP-kanMX6/RAP1-GFP-kanMX6*	diploid	Sdl-728 **x** Sdl-736	this study

**Strains used for meiotic segregation analyses**							
Sdl-100	YLNH55			*MATα*, meiotic descendant of wild-type isolate Sdl-75	haploid		this study
Sdl-102	YLNH57			*MATa*, meiotic descendant of wild-type isolate Sdl-75	haploid		this study
Sdl-122	YLNH75			*MATa*, meiotic descendant of cross Sdl-100 **x** Sdl-102	haploid		this study
Sdl-982	YLFP188-1			*MATa/MATα*	diploid	Sdl-9752 **x** Sdl-122	this study
Tetrad Sdl-113 - Sdl-116							
Sdl-113	YLNH66	}	first	meiotic descendant of cross Sdl-100 **x** Sdl-102	haploid		this study
Sdl-114	YLNH67		dyad	meiotic descendant of cross Sdl-100 **x** Sdl-102	haploid		this study
Sdl-115	YLNH68	}	second	meiotic descendant of cross Sdl-100 **x** Sdl-102	haploid		this study
Sdl-116	YLNH69		dyad	meiotic descendant of cross Sdl-100 **x** Sdl-102	haploid		this study
Tetrad Sdl-117 - Sdl-120							
Sdl-117	YLNH70	}	first	meiotic descendant of cross Sdl-100 **x** Sdl-102	haploid		this study
Sdl-118	YLNH71		dyad	meiotic descendant of cross Sdl-100 **x** Sdl-102	haploid		this study
Sdl-119	YLNH72	}	second	meiotic descendant of cross Sdl-100 **x** Sdl-102	haploid		this study
Sdl-120	YLNH73		dyad	meiotic descendant of cross Sdl-100 **x** Sdl-102	haploid		this study
Tetrad Sdl-280 - Sdl-283							
Sdl-280	YLNH198	}	first	meiotic descendant of cross Sc-9752 **x** Sdl-122	haploid		this study
Sdl-281	YLNH199		dyad	meiotic descendant of cross Sc-9752 **x** Sdl-122	haploid		this study
Sdl-282	YLNH200	}	second	meiotic descendant of cross Sc-9752 **x** Sdl-122	haploid		this study
Sdl-283	YLNH201		dyad	meiotic descendant of cross Sc-9752 **x** Sdl-122	haploid		this study
Tetrad Sdl-284 - Sdl-287							
Sdl-284	YLNH202	}	first	meiotic descendant of cross Sc-9752 **x** Sdl-122	haploid		this study
Sdl-285	YLNH203		dyad	meiotic descendant of cross Sc-9752 **x** Sdl-122	haploid		this study
Sdl-286	YLNH204	}	second	meiotic descendant of cross Sc-9752 **x** Sdl-122	haploid		this study
Sdl-287	YLNH205		dyad	meiotic descendant of cross Sc-9752 **x** Sdl-122	haploid		this study
Tetrad Sdl-985 - Sdl-988							
Sdl-985	YLFP189-1	}	first	meiotic descendant of cross Sc-9752 **x** Sdl-122	haploid		this study
Sdl-986	YLFP189-2		dyad	meiotic descendant of cross Sc-9752 **x** Sdl-122	haploid		this study

Sdl-987	YLFP189-3	second	meiotic descendant of cross Sc-9752 **x** Sdl-122	haploid	this study
Sdl-988	YLFP189-4	dyad	meiotic descendant of cross Sc-9752 **x** Sdl-122	haploid	this study
Tetrad Sdl-989 - Sdl-992					
Sdl-989	YLFP190-1	first	meiotic descendant of cross Sc-9752 **x** Sdl-122	haploid	this study
Sdl-990	YLFP190-2	dyad	meiotic descendant of cross Sc-9752 **x** Sdl-122	haploid	this study
Sdl-991	YLFP190-3	second	meiotic descendant of cross Sc-9752 **x** Sdl-122	haploid	this study
Sdl-992	YLFP190-4	dyad	meiotic descendant of cross Sc-9752 **x** Sdl-122	haploid	this study
Tetrad Sdl-1233 - Sdl-1239					
Sdl-1233	YLIP2-1	first	meiotic descendant of cross Sc-9752 **x** Sdl-122	haploid	this study
Sdl-1235	YLIP3-1	dyad	meiotic descendant of cross Sc-9752 **x** Sdl-122	haploid	this study
Sdl-1237	YLIP4-1	second	meiotic descendant of cross Sc-9752 **x** Sdl-122	haploid	this study
Sdl-1239	YLIP5-1	dyad	meiotic descendant of cross Sc-9752 **x** Sdl-122	haploid	this study
**Additional strains used for population comparisons/analyses**					
Sdl-58	CBS 1168		wild-type isolate	haploid	
Sdl-1211	BJK_5C		wild-type isolate (origin: Slovenia)	diploid	[38]
Sc-9673	NCYC 732		wild-type isolate (origin: Italy)	diploid	
Sc-9676	NCYC 734		wild-type isolate (origin: UK)	diploid	
Sc-9679	NCYC 849		wild-type isolate (origin: UK)	diploid	
Sc-9682	NCYC 3345		wild-type isolate	aneuploid (2n+1)	
Sc-9790	DSM 70550		wild-type isolate (origin: France)	aneuploid (2n+1)	
Sdl-1267	PC99_R_1		meiotic descendant of wild-type isolate PC99/R (origin: Italy)	haploid	[39]

[1] Database ID numbers of yeast strains correspond to their permanent identifiers in the Knop lab yeast strain collections.

[2] Wild-type diploid isolate from grape must in Japan; isolated in 1964; alternative strain identifiers: IFO 1721, DSM 3447, O-81.

[3] Single-spore meiotic descendant of wild-type diploid isolate NBRC 1721; alternative strain identifiers: ATCC 26617, DSM 3448, IFO 1722, IIA$_{11}$S1.

[4] Single-spore meiotic descendant of wild-type diploid isolate NBRC 1721; alternative strain identifiers: ATCC 26618, DSM 3449, IFO 1723, IIA$_{11}$S2.

[5] Wild-type diploid isolate from garden soil in Pretoria (South Africa); isolated in 1953.
