# Supplementary material for: Sex without crossing over in the yeast Saccharomycodes ludwigii
Source: Genome Biol. 2021 Nov 3;22:303. doi: 10.1186/s13059-021-02521-w (PMC8567612; doi:10.1186/s13059-021-02521-w)
Supplement: Supplementary file 2 — Additional file 2: Figs. S1-S6. [file 13059_2021_2521_MOESM2_ESM.pdf]

Supplementary Figures S1-S6

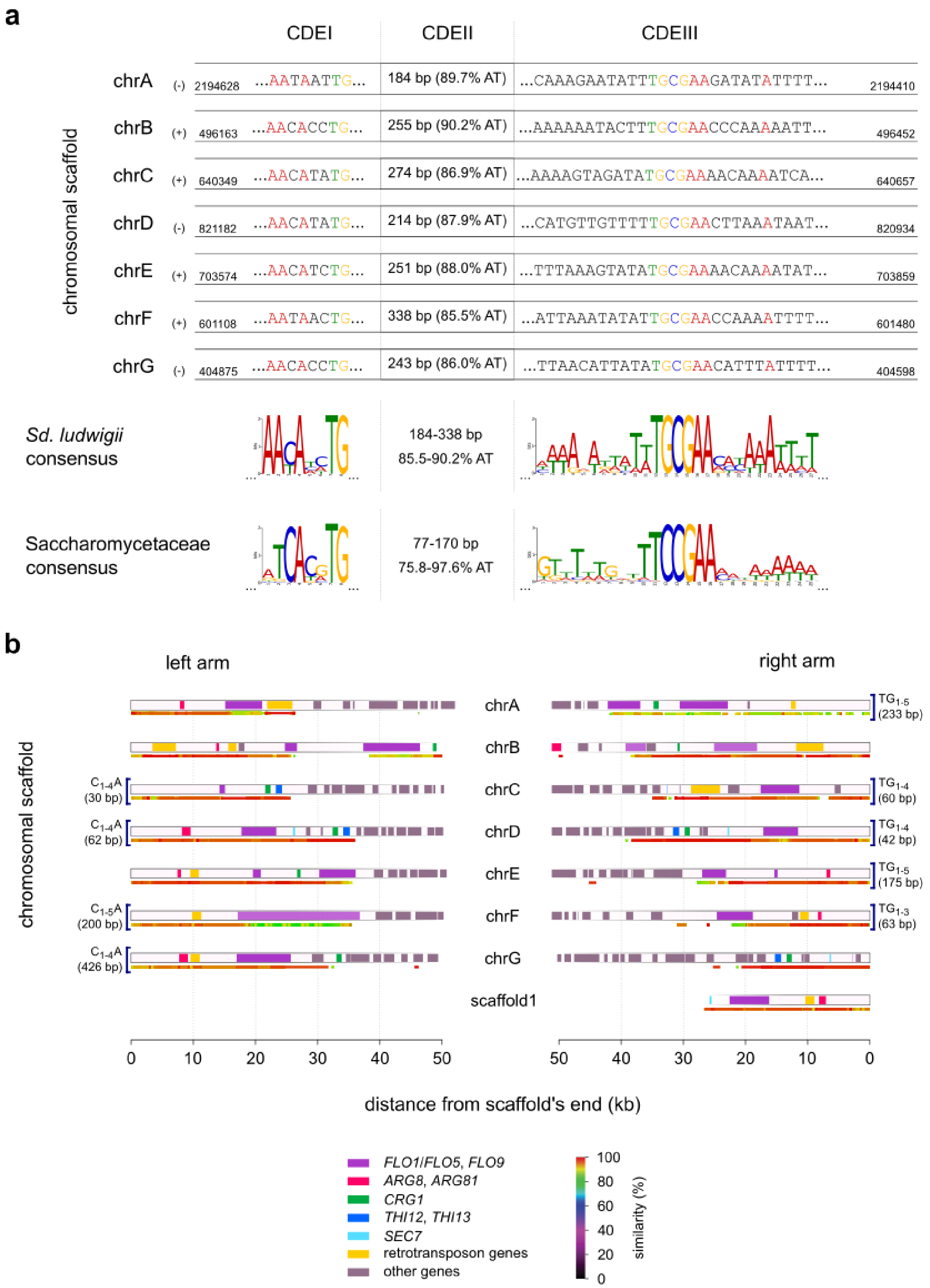

Fig. S1 (See legend on next page.)

(See figure on previous page.)
Fig. S1 Features of *Sd. ludwigii* point centromeres and repetitive (sub)telomeres. **a** Sequences of the centromere DNA elements (CDEs) **I** and **III**, and length and AT content of CDE **II** of each predicted centromere. The direction of each centromere with reference to the genomic scaffold (+/-) is provided next to its bar (left). The consensus DNA motif sequences of CDEs **I** and **III** and the ranges of length and AT content of CDEs **II** are shown (bottom), in comparison to the respective Saccharomycetaceae data. **b** The terminal regions of all chromosomal scaffolds are shown. A horizontal line beneath each bar indicates coverage by subtelomere-specific DNA repeats that are shared between different subtelomeres. These coverage bars are color-coded on the basis of the highest sequence similarity to other subtelomeres, according to the color key (bottom right). Detected telomere-specific DNA motif sequences at the ends of chromosomal scaffolds are indicated by square brackets. Gene content/locations for each subtelomere are shown as boxes within the bars, colored according to the respective code (bottom left). Gene homologs typical of *S. cerevisiae* subtelomeres, such as *FLO* and *ARG* gene family members, are present in all *Sd. ludwigii* subtelomeres, while retrotransposons are also present in most of them

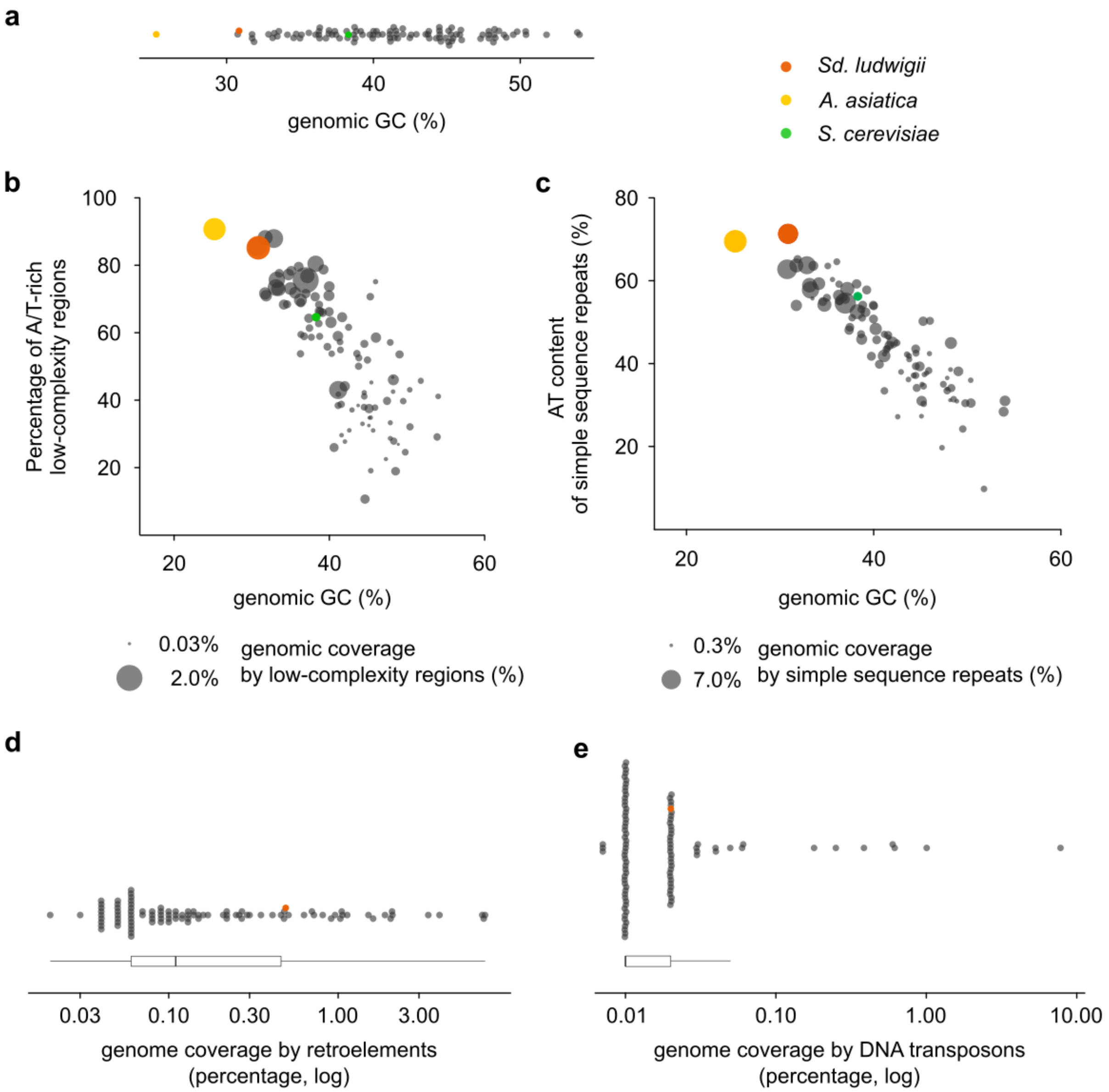

Fig. S2 (See legend on next page.)

(See figure on previous page.)
Fig. S2 A comparative genomic analysis with 100 yeast and other fungal genomes revealed that the *Sd. ludwigii* genome is exceptionally AT-rich and enriched in low-complexity, repetitive, and transposable elements. **a** Overall genomic GC content. **b** Genomic coverage by low-complexity regions (dot width) and the proportion of those regions that are AT-rich (>70%; y axis) versus the overall genomic GC content (x axis). **c** Genomic coverage by simple sequence repeats (SSRs or microsatellites; dot width) and the total AT content of SSRs (y axis) versus the mean genomic GC content (x axis). **d, e** Genomic coverage by retroelements (**d**) and DNA transposons (**e**). All genomes included in the comparisons here are listed in Table S2

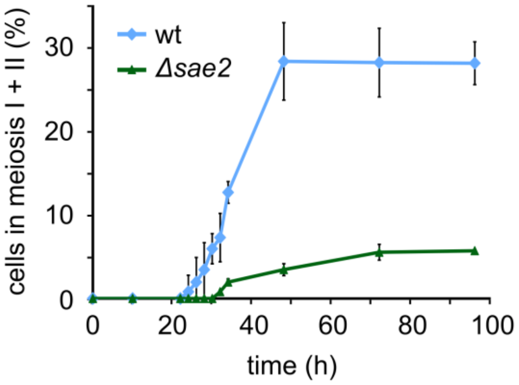

Fig. S3 *Sd. ludwigii* Sae2 is necessary for normal sporulation, similarly to Spo11. Meiotic time-course analysis of a homozygous Δ*sae2* deletion strain in comparison to the wild type. Following induction of cells to enter meiosis, samples were withdrawn at the indicated time points and their cellular DNA content was stained with Hoechst 33258 to determine binucleate (meiosis I) and tetranucleate (meiosis II) cells. Error bars: SD (*n* = 3 replicates)

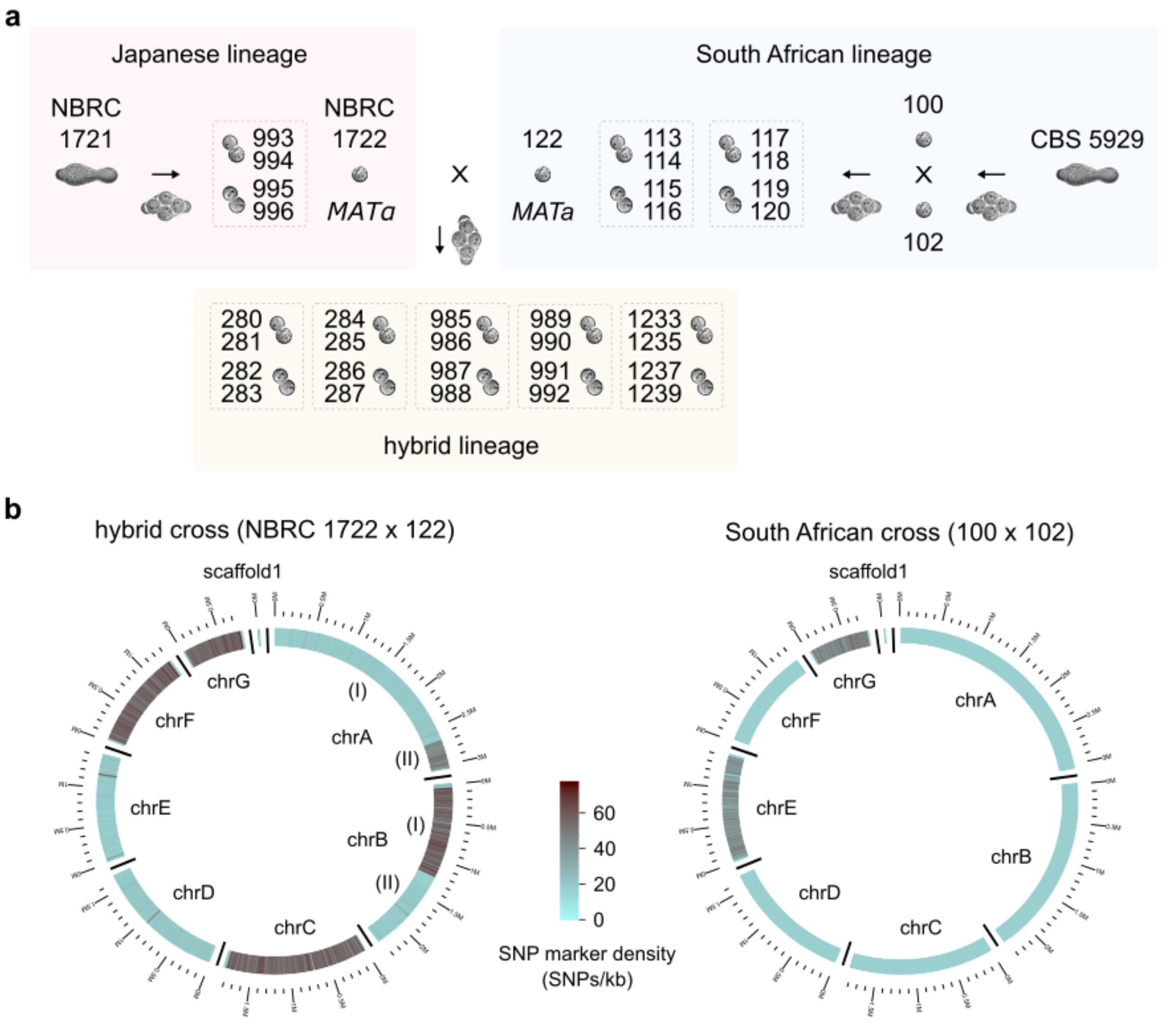

chromosome	sequence divergence (%)	distance between adjacent SNPs (mean, median)
chrA (I)	0.20	555, 286
chrA (II)	2.42	149, 28
chrB (I)	3.36	30, 14
chrB (II)	0.19	525, 192
chrC	3.31	30, 15
chrD	0.29	348, 172
chrE	0.26	413, 84
chrF	3.41	29, 15
chrG	3.38	29, 14
scaffold1	0.26	290, 23

chromosome	sequence divergence (%)	distance between adjacent SNPs (mean, median)
chrE	1.94	51, 15
chrG	2.38	42, 13
others	essentially homozygous	

Fig. S4 Meiotic SNP segregation analysis in *Sd. ludwigii*. **a** A detailed scheme of the experimental setup, including the identifiers of all strains used: the wild-type isolates are referred to with their original strain collection IDs, whereas the Knop lab *Sd. ludwigii* strain collection IDs are provided for all other strains that were generated in this study (Table S1). Spore viability of strain CBS 5929 was very low (probably due to high load of deleterious mutations); it was thus impossible to obtain full viable tetrads from this strain for the SNP segregation analysis. We used backcrossing to isolate two spores (spores 100 and 102) that gave rise to diploids with high spore viability. **b** Heterozygosity levels (expressed as the number of filtered high-quality SNPs per kb of genomic sequence) between the parental strains of each cross are plotted along the chromosomes of the reference genome, and statistics of SNPs for each chromosome are provided in the tables below

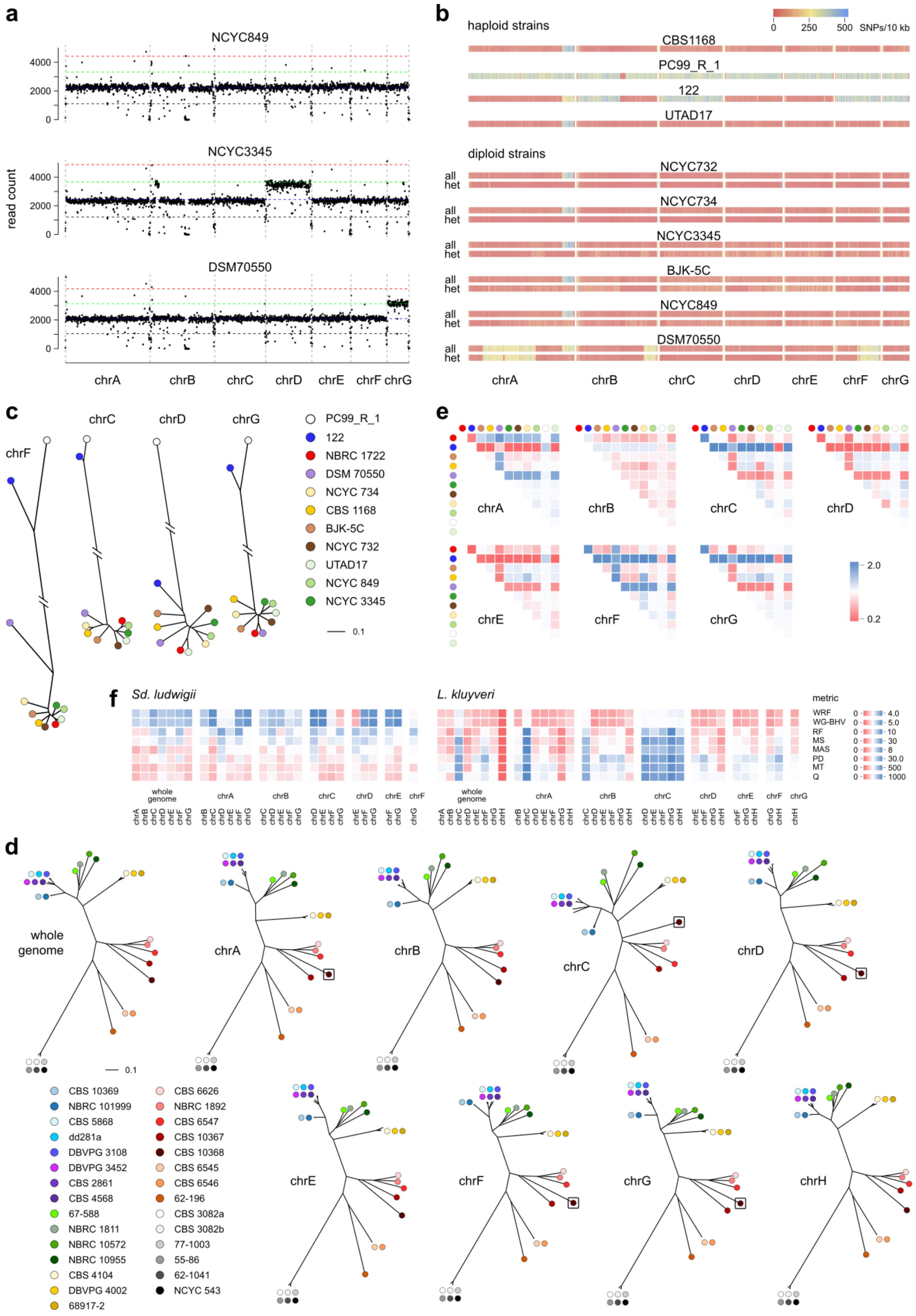

Fig. S5 (See legend on next page.)

(See figure on previous page.)
Fig. S5 Genomic comparisons of *Sd. ludwigii* strains. **a** Examples of euploid (NCYC 849) and aneuploid (chrD and chrG trisomies in NCYC 3345 and DSM 70550, respectively) isolates of *Sd. ludwigii*, determined by read coverage analysis. The gap near the middle of chrB corresponds to the rDNA region. **b** Genome-wide distribution of SNPs of all *Sd. ludwigii* strains in comparison to the reference strain. For each diploid strain, the total number of SNPs ("all") is shown in the upper bar and the heterozygous positions ("het") in the bottom one. **c** Dendrograms of *Sd. ludwigii* strains, constructed by hierarchical cluster analysis of SNPs of individual chromosomes. The dendrograms of the remaining chromosomes, as well as that of the whole genome, are shown in Fig. 6a. **d** Similarly constructed dendrograms of *L. kluyveri* strains. **e** Differences in SNP density of individual chromosomes of *Sd. ludwigii* in comparison to the whole genome, for all strains (color code as in c). **f** Tree difference scores (seven unrooted metrics, no normalization) between all combinations of whole-genome SNPs and SNPs of individual chromosomes, for *Sd. ludwigii* and *L. kluyveri*. The analyzed strains are shown in panels (**c**) and (**d**), respectively. WRF: weighted Robinson-Foulds distance. WG-BHV: weighted geodesic (BHV) unrooted distance. RF: Robinson-Foulds distance. MS: matching split distance. MAS: unrooted maximum agreement subtree distance. PD: path difference distance. MT: matching triplet distance. Q: quartet distance

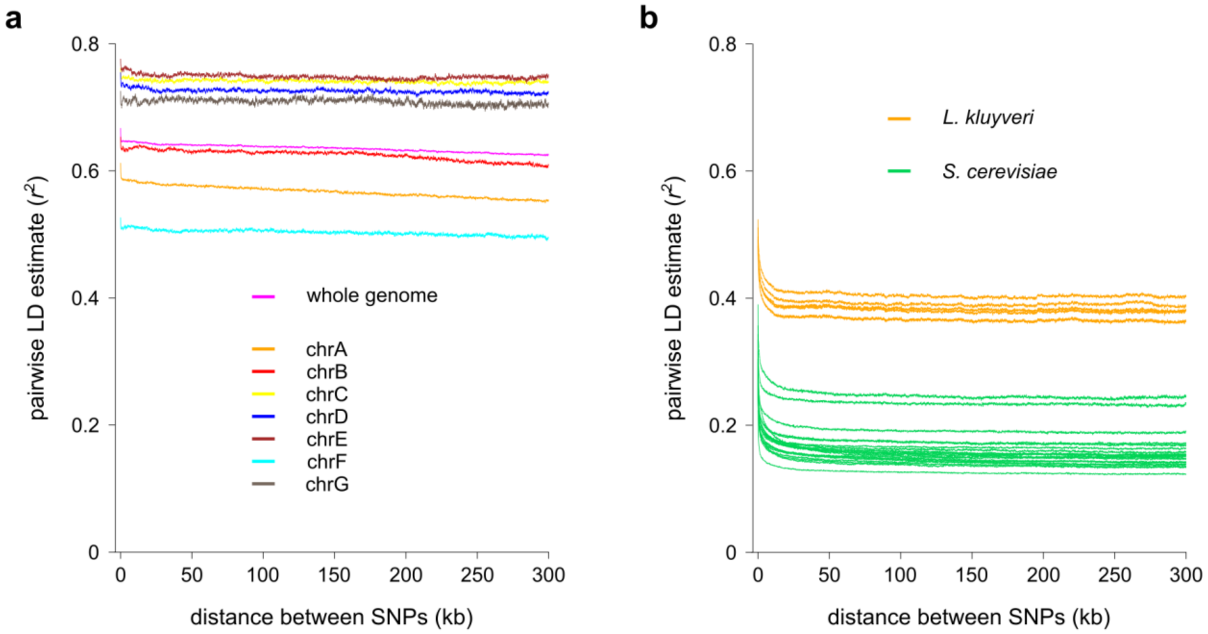

Fig. S6 Curves of linkage disequilibrium (LD) decay as a function of physical distance between SNP marker pairs. **a** LD decay curves of the whole genome in comparison to individual chromosomes of *Sd. ludwigii*. **b** LD decay curves of 5 *L. kluyveri* and 20 *S. cerevisiae* groups of strains (of the same sample size, n=11)
