# Supplementary material for: Sex without crossing over in the yeast Saccharomycodes ludwigii
Source: Genome Biol. 2021 Nov 3;22:303. doi: 10.1186/s13059-021-02521-w (PMC8567612; doi:10.1186/s13059-021-02521-w)
Supplement: Supplementary file 3 — Additional file 3: Table S2. Genome assemblies of yeasts and filamentous fungi. [file 13059_2021_2521_MOESM3_ESM.pdf]

Table S2 List of genome assemblies of yeasts and filamentous fungi analyzed in this study

Kingdom	Subkingdom	Phylum	Class	Order	Family			#	Species	Strain	Assembly	Accession	Scaffolds	Contigs	N50	L50	Assembly level	Database
Fungi	Dikarya	Ascomycota	Saccharomycetes	Saccharomycetales	Saccharomycetaceae and related families	Saccharomycetaceae	post-WGD	1	*Candida glabrata*	CBS 138	GCA_000002545.2	ASM254v2	14	17	1100349	5	chromosome	NCBI Genome
								2	*Kazachstania africana*	CBS 2517	GCA_000304475.1	Ka_CBS2517	12	34	925516	5	chromosome	NCBI Genome
								3	*Kazachstania naganishii*	CBS 8797	GCA_000348985.1	ASM34898v1	13	95	227487	12	chromosome	NCBI Genome
								4	*Naumovozyma castellii*	CBS 4309	GCA_000237345.1	ASM23734v1	10	17	1245273	3	chromosome	NCBI Genome
								5	*Naumovozyma dairenensis*	CBS 421	GCA_000227115.2	ASM22711v2	11	279	109578	37	chromosome	NCBI Genome
								6	*Saccharomyces arboricola*	H-6 (CBS 10644)	GCA_000292725.1	SacArb1.0	35	219	117287	35	chromosome	NCBI Genome
								7	*Saccharomyces cerevisiae*	S288C	S288C_reference_R64_2_1 (SGD)	R64_2_1 (SGD)	17	17	924431	6	complete genome	SGD
								8	*Saccharomyces eubayanus*	FM1318	GCA_001298625.1	SEUB3.0	24	133	197643	17	chromosome	NCBI Genome
								9	*Saccharomyces paradoxus*	CBS 432	GCA_002079055.1	ASM207905v1	17	18	835812	6	chromosome	NCBI Genome
								10	*Tetrapisispora blattae*	CBS 6284	GCA_000315915.1	ASM31591v1	10	82	286437	18	chromosome	NCBI Genome
								11	*Tetrapisispora phaffii*	CBS 4417	GCA_000236905.1	ASM23690v1	17	105	249073	18	chromosome	NCBI Genome
							ZT clade	12	*Torulaspora delbrueckii*	CBS 1146	GCA_000243375.1	ASM24337v1	8	34	539679	6	chromosome	NCBI Genome
								13	*Zygosaccharomyces bailii*	CLIB 213	GCA_000442885.1	ZYBA0	27	287	84072	38	scaffold	NCBI Genome
								14	*Zygosaccharomyces rouxii*	CBS 732	GCA_000026365.1	ASM2636v1	7	9	1496342	3	chromosome	NCBI Genome
							KLE clade	15	*Eremothecium coryli*	CBS 5749	GCA_000710315.1	Eremothecium coryli	19	63	324660	9	scaffold	NCBI Genome
								16	*Eremothecium cymbalariae*	DBVPG 7215	GCA_000235365.1	ASM23536v1	8	9	1193613	4	chromosome	NCBI Genome
								17	*Eremothecium gossypii*	ATCC 10895	GCA_000091025.4	ASM9102v4	8	8	1519140	3	complete genome	NCBI Genome
								18	*Kluyveromyces dobzhanskii*	CBS 2104	GCA_000820885.1	KLDO_01	86	86	493016	7	contig	NCBI Genome
								19	*Kluyveromyces lactis*	NRRL Y-1140	GCA_000002515.1	ASM251v1	7	7	1753957	3	complete genome	NCBI Genome
								20	*Kluyveromyces marxianus*	NBRC 1777	GCA_001417835.1	KM1777_03	9	9	1422013	4	complete genome	NCBI Genome
								21	*Lachancea dasiensis*	CBS 10888	GCA_900074725.1	LADA0	8	31	988707	5	chromosome	NCBI Genome
								22	*Lachancea fantastica* (sp. PJ-2012a)	CBS 6924	GCA_900074735.1	LAFA0	7	25	730885	6	chromosome	NCBI Genome
								23	*Lachancea fermentati*	CBS 6772	GCA_900074765.1	LAFE0	8	36	821294	6	chromosome	NCBI Genome
								24	*Lachancea kluyveri*	NRRL Y-12651	GCA_000149225.1	ASM14922v1	32	35	1252455	5	chromosome	NCBI Genome
								25	*Lachancea lanzarotensis*	CBS 12615	GCA_000938715.1	LALA0	24	34	700478	6	scaffold	NCBI Genome
								26	*Lachancea meyersii*	CBS 8951	GCA_900074715.1	LAME0	8	27	716495	6	chromosome	NCBI Genome
								27	*Lachancea mirantina*	CBS 11717	GCA_900074745.1	LAMI0	8	20	1114975	4	chromosome	NCBI Genome
								28	*Lachancea nothofagi*	CBS 11611	GCA_900074755.1	LANO0	8	37	994480	4	chromosome	NCBI Genome
								29	*Lachancea thermotolerans*	CBS 6340	GCA_000142805.1	ASM14280v1	8	10	1513537	4	chromosome	NCBI Genome
						Saccharomycodaceae		30	*Hanseniaspora opuntiae*	AWRI 3578	GCA_001749795.1	ASM174979v1	17	66	240102	11	scaffold	NCBI Genome
								31	*Hanseniaspora osmophila*	AWRI 3579	GCA_001747045.1	ASM174704v1	17	899	18891	178	scaffold	NCBI Genome
								32	*Hanseniaspora uvarum*	AWRI 3580	GCA_001747055.1	ASM174705v1	18	44	453480	6	scaffold	NCBI Genome
						Phaffomycetaceae		33	*Cyberlindnera fabianii*	JCM 3601	GCA_001599195.1	JCM_3601_assembly_v001	12	53	450523	7	scaffold	NCBI Genome
								34	*Cyberlindnera jadinii*	NRRL Y-1542	GCA_001661405.1	Cybja1	76	392	111555	34	scaffold	NCBI Genome
								35	*Wickerhamomyces anomalus*	NRRL Y-366-8	GCA_001661255.1	Wican1	46	222	185283	23	scaffold	NCBI Genome
						Saccharomycopsidaceae		36	*Saccharomycopsis fibuligera*	KPH12	GCA_001936155.1	KPH12	7	7	3000806	3	complete genome	NCBI Genome
								37	*Saccharomycopsis malanga*	JCM 7620	GCA_001599215.1	JCM_7620_assembly_v001	44	229	190130	23	scaffold	NCBI Genome
						Ascoideaceae		38	*Ascoidea asiatica*	JCM 7603	GCA_001600695.1	JCM_7603_assembly_v001	71	264	236462	24	scaffold	NCBI Genome
								39	*Ascoidea rubescens*	DSM 1968	GCA_001661345.1	Ascru1	63	326	171347	33	scaffold	NCBI Genome
					CTG clade	Debaryomycetaceae		40	*Babjeviella inositovora*	NRRL Y-12698	GCA_001661335.1	Babin1	49	211	198400	25	scaffold	NCBI Genome
								41	*Candida albicans*	SC5314	GCA_000182965.3	ASM18296v3	8	88	334280	12	chromosome	NCBI Genome
								42	*Candida carpophila*	JCM 9396	GCA_001599235.1	JCM_9396_assembly_v001	10	26	765813	5	scaffold	NCBI Genome
								43	*Candida dubliniensis*	CD36	GCA_000026945.1	ASM2694v1	8	8	2267510	3	complete genome	NCBI Genome
								44	*Candida orthopsilosis*	Co 90-125	GCA_000315875.1	ASM31587v1	8	242	120081	36	chromosome	NCBI Genome
								45	*Candida parapsilosis*	CDC317	GCA_000182765.2	ASM18276v2	9	9	2091826	3	scaffold	NCBI Genome
								46	*Candida tanzawaensis*	NRRL Y-17324	GCA_001661415.1	Canta1	16	249	183924	21	scaffold	NCBI Genome
								47	*Candida tenuis*	ATCC 10573	GCA_000223465.1	Candida tenuis v1.0	25	74	382504	12	scaffold	NCBI Genome
								48	*Candida tropicalis*	MYA-3404	GCA_000006335.3	ASM633v2	24	128	221103	21	scaffold	NCBI Genome
								49	*Debaryomyces hansenii*	CBS 767	GCA_000006445.2	ASM644v2	8	15	993818	5	chromosome	NCBI Genome
								50	*Lodderomyces elongisporus*	NRRL YB-4239	GCA_000149685.1	ASM14968v1	28	145	261145	20	scaffold	NCBI Genome
								51	*Meyerozyma caribbica*	MG20W	GCA_000755205.1	ASM75520v1	9	9	1673638	3	contig	NCBI Genome
								52	*Meyerozyma guilliermondii*	ATCC 6260	GCA_000149425.1	ASM14942v1	9	71	313225	12	scaffold	NCBI Genome
								53	*Millerozyma acaciae*	JCM 10732	GCA_001600675.1	JCM_10732_assembly_v001	10	29	778428	6	scaffold	NCBI Genome
								54	*Priceomyces haplophilus*	JCM 1635	GCA_001599895.1	JCM_1635_assembly_v001	9	29	598910	8	scaffold	NCBI Genome
								55	*Scheffersomyces lignosus*	JCM 9837	GCA_001599395.1	JCM_9837_assembly_v001	19	73	619226	8	scaffold	NCBI Genome
								56	*Scheffersomyces stipitis*	CBS 6054	GCA_000209165.1	ASM20916v1	8	9	1803401	4	chromosome	NCBI Genome
								57	*Spathaspora arborariae*	UFMG-19.1A	GCA_000497715.1	SpaArb1.0	41	381	63812	59	scaffold	NCBI Genome
								58	*Spathaspora passalidarum*	NRRL Y-27907	GCA_000223485.1	Spathaspora passalidarum v2.0	8	26	1645821	4	scaffold	NCBI Genome
						Metschnikowiaceae		59	*Candida intermedia*	CBS 141442	GCA_900106115.1	CBS 141442 assembly	8	10	1965937	4	chromosome	NCBI Genome
								60	*Clavispora lusitaniae*	ATCC 42720	GCA_000003835.1	ASM383v1	9	88	266609	15	scaffold	NCBI Genome
								61	*Metschnikowia bicuspidata*	NRRL YB-4993	GCA_001664035.1	Metbi1	48	582	62344	72	scaffold	NCBI Genome
								62	*Metschnikowia fructicola*	277	GCA_000317355.2	ASM31735v2	93	93	957836	9	contig	NCBI Genome
						related species *incertae sedis*		63	*Hyphopichia burtonii*	NRRL Y-1933	GCA_001661395.1	Hypbu1	27	243	114103	34	scaffold	NCBI Genome
								64	*Hyphopichia homilentoma*	JCM 1507	GCA_001599095.1	JCM_1507_assembly_v001	8	32	708264	6	scaffold	NCBI Genome
								65	*Wickerhamia fluorescens*	JCM 1821	GCA_001599155.1	JCM_1821_assembly_v001	20	109	312802	15	scaffold	NCBI Genome
					Methylotrophs	Pichiaceae		66	*Brettanomyces anomalus*	CBS 7654	GCA_001754015.1	ASM175401v1	28	261	123727	27	scaffold	NCBI Genome
								67	*Brettanomyces bruxellensis*	CBS 2499	GCA_000340765.1	Dekkera bruxellensis CBS 2499 v2.0	63	887	30855	105	scaffold	NCBI Genome
								68	*Brettanomyces naardenensis*	CBS 7540	GCA_001753995.1	ASM175399v1	76	104	355666	10	scaffold	NCBI Genome
								69	*Candida arabinofermentans*	NRRL YB-2248	GCA_001661425.1	Canar1	62	701	101778	39	scaffold	NCBI Genome
								70	*Candida boidinii*	JCM 9604	GCA_001599335.1	JCM_9604_assembly_v001	32	144	512251	12	scaffold	NCBI Genome
								71	*Candida succiphila*	JCM 9445	GCA_001599255.1	JCM_9445_assembly_v001	22	64	546973	8	scaffold	NCBI Genome
								72	*Komagataella phaffii*	CBS 7435	GCA_000223565.1	PicPas_Mar2011	5	17	1320048	3	chromosome	NCBI Genome
								73	*Ogataea methanolica*	JCM 10240	GCA_001600755.1	JCM_10240_assembly_v001	32	168	223418	23	scaffold	NCBI Genome
								74	*Ogataea parapolymorpha*	DL-1	GCA_000187245.3	Hansenula_2	7	10	1273462	4	chromosome	NCBI Genome
								75	*Ogataea polymorpha*	NCYC 495 leu1.1	GCA_001664045.1	Hanpo2	7	7	1302532	4	contig	NCBI Genome
								76	*Pichia membranifaciens*	NRRL Y-2026	GCA_001661235.1	Picme2	10	144	472039	9	scaffold	NCBI Genome
								77	*Nakazawaea peltata*	JCM 9829	GCA_001599355.1	JCM_9829_assembly_v001	11	34	648860	5	scaffold	NCBI Genome
						incertae sedis		78	*Ambrosiozyma kashinagacola*	JCM 15019	GCA_001599075.1	JCM_15019_assembly_v001	23	59	418399	8	scaffold	NCBI Genome
								79	*Candida sorboxylosa*	JCM 1536	GCA_001599115.1	JCM_1536_assembly_v001	37	277	118414	24	scaffold	NCBI Genome
								80	*Kuraishia capsulata*	CBS 1993	GCA_000576695.1	AUH_PRJEB4427_v1	7	73	370915	10	scaffold	NCBI Genome
					Basal lineages	Dipodascaceae		81	*Sporopachydermia quercuum*	JCM 9486	GCA_001599295.1	JCM_9486_assembly_v001	15	37	852576	6	scaffold	NCBI Genome
								82	*Yarrowia deformans*	JCM 1694	GCA_001600075.1	JCM_1694_assembly_v001	44	174	264734	24	scaffold	NCBI Genome
								83	*Yarrowia keelungensis*	JCM 14894	GCA_001600195.1	JCM_14894_assembly_v001	41	180	288559	24	scaffold	NCBI Genome
								84	*Yarrowia lipolytica*	CLIB 89 (W29)	GCA_001761485.1	ASM176148v1	7	7	3629463	3	complete genome	NCBI Genome
						Trichomonascaceae		85	*Sugiyamaella lignohabitans*	CBS 10342	GCA_001640025.2	ASM164002v2	4	4	4956242	2	complete genome	NCBI Genome
								86	*Wickerhamiella domercqiae*	JCM 9478	GCA_001599275.1	JCM_9478_assembly_v001	4	50	440821	8	scaffold	NCBI Genome
						Trigonopsidaceae		87	*Tortispora caseinolytica*	NRRL Y-17796	GCA_001661475.1	Canca1	6	64	572105	6	scaffold	NCBI Genome
						incertae sedis		88	*Candida apicola*	NRRL Y-50540	GCA_001005415.1	ASM100541v1	40	135	182354	17	scaffold	NCBI Genome
								89	*Nadsonia fulvescens*	DSM 6958	GCA_001661315.1	Nadfu1	20	200	200547	21	scaffold	NCBI Genome
								90	*Starmerella bombicola*	JCM 9596	GCA_001599315.1	JCM_9596_assembly_v001	16	63	662740	6	scaffold	NCBI Genome
			Leotiomycetes	Helotiales	Sclerotiniaceae			91	*Botrytis cinerea*	B05.10	GCA_000832945.1	ASM83294v1	18	18	2617329	7	complete genome	NCBI Genome
			Sordariomycetes	Glomerellales	Plectosphaerellaceae			92	*Verticillium dahliae*	JR2	GCA_000400815.2	VDAG_JR2v.4.0	8	8	4168633	4	complete genome	NCBI Genome
				Sordariales	Sordariaceae			93	*Neurospora crassa*	OR74A	GCA_000182925.2	NC12	21	412	656009	21	chromosome	NCBI Genome
			Dothideomycetes	Pleosporales	Pleosporaceae			94	*Bipolaris maydis*	C5	GCA_000338975.1	CocheC5_3	68	88	1168586	12	scaffold	NCBI Genome
			Eurotiomycetes	Eurotiales	Aspergillaceae			95	*Aspergillus nidulans*	FGSC A4	GCA_000011425.1	ASM1142v1	8	82	679860	13	chromosome	NCBI Genome
				Onygenales	*incertae sedis*			96	*Coccidioides immitis*	RS	GCA_000149335.2	ASM14933v2	7	11	4311478	3	scaffold	NCBI Genome
			Schizosaccharomycetes	Schizosaccharomycetales	Schizosaccharomycetaceae			97	*Schizosaccharomyces pombe*	972h-	GCA_000002945.2	ASM294v2	4	8	2923134	2	chromosome	NCBI Genome
		Basidiomycota	Tremellomycetes	Tremellales	Cryptococcaceae			98	*Cryptococcus neoformans*	JEC21	GCA_000091045.1	ASM9104v1	14	37	1076541	7	chromosome	NCBI Genome
			Ustilaginomycetes	Ustilaginales	Ustilaginaceae			99	*Ustilago maydis*	521	GCA_000328475.2	Umaydis521_2.0	27	254	129796	47	chromosome	NCBI Genome
		Mucoromycota		Mucorales	Rhizopodaceae			100	*Rhizopus delemar*	RA 99-880	GCA_000149305.1	RO3	83	391	303,659	46	scaffold	NCBI Genome
