# Supplementary material for: Sex without crossing over in the yeast Saccharomycodes ludwigii
Source: Genome Biol. 2021 Nov 3;22:303. doi: 10.1186/s13059-021-02521-w (PMC8567612; doi:10.1186/s13059-021-02521-w)
Supplement: Supplementary file 4 — Additional file 4: Table S3. Genes involved in meiosis/meiotic recombination and their presence/absence in Sd. ludwigii and other yeasts. [file 13059_2021_2521_MOESM4_ESM.pdf]

Table S3 Genes involved in meiosis/meiotic recombination and their presence/absence in *Sd. ludwigii* and other yeasts

Saccharomyces cerevisiae genes involved in meiosis / meiotic recombination											post-WGD species												
Systematic name	**Standard name** (aliases) | paralogs	***Saccharomycodes ludwigii***	*Lachancea waltii*	*Lachancea thermotolerans*	*Lachancea kluyveri*	*Eremothecium cymbalariae*	*Eremothecium gossypii*	*Kluyveromyces lactis*	*Torulaspora delbrueckii*	*Zygosaccharomyces rouxii*	*Vanderwaltozyma polyspora*	*Tetrapisispora phaffii*	*Tetrapisispora blattae*	*Naumovozyma dairenensis*	*Naumovozyma castellii*	*Kazachstania naganishii*	*Kazachstania africana*	*Candida glabrata*	*Saccharomyces bayanus*	*Saccharomyces kudriavzevii*	*Saccharomyces mikatae*	***Saccharomyces cerevisiae***	***Saccharomycodes ludwigii*** predicted homolog
**Meiotic recombination and related processes - double-strand break (DSB) formation and repair**																							
**Mechanisms**																							
YDL074C	***BRE1***																						*Slud2402*
YNR051C	***BRE5***																						*Slud1720*
YMR001C	***CDC5*** (*MSD2*, *PKX2*)																						*Slud3353*
YLR394W	***CST9*** (*ZIP3*)																						*Slud2415*
YER179W	***DMC1*** (*ISC2*)																						*Slud3934*
YHR164C	***DNA2*** (*WEB2*)																						*Slud3099*
YDR446W	***ECM11***																						*Slud3211*
YOR033C | YDR263C	***EXO1*** (*DHS1*) | ***DIN7*** (*DIN3*)																						*Slud3014*
YDR110W	***FOB1*** (*HRM1*)																						*Slud3944*
YLR445W	***GMC2***																						*Slud2339*
YGL251C	***HFM1*** (*MER3*)																						*Slud0435*
YGL033W	***HOP2***																						*Slud3835*
YBR136W	***MEC1*** (*RAD31*, *ESR1*, *SAD3*)																						*Slud0702*
YER044C-A	***MEI4***																						*Slud0093*
YPL121C	***MEI5*** (*LPH6*)																						*Slud4159*
YNL210W	***MER1***																						-
YMR167W	***MLH1*** (*PMS2*)																						*Slud0795*
YLR035C	***MLH2***																						*Slud0579*
YPL164C	***MLH3***																						*Slud4111*
YBR098W	***MMS4*** (*SLX2*)																						*Slud0400*
YGL183C	***MND1***																						*Slud3742*
YIR025W	***MND2***																						*Slud3464*
YIR002C	***MPH1***																						*Slud4562*
YMR224C	***MRE11*** (*RAD58*, *XRS4*, *NGS1*)																						*Slud1978*
YML128C	***MSC1***																						*Slud3684*
YLR219W	***MSC3***																						*Slud4336*
YOR354C	***MSC6***																						*Slud1336*
YHR039C	***MSC7***																						*Slud2841*
YFL003C	***MSH4***																						*Slud1732*
YDL154W	***MSH5***																						*Slud1404*
YDR386W	***MUS81*** (*SLX3*)																						*Slud1232*
YOL104C	***NDJ1*** (*TAM1*)																						*Slud0702*
YDL105W	***NSE4*** (*QRI2*)																						*Slud0794*
YOL115W | YNL299W	***PAP2*** (*TRF4*) | ***TRF5***																						*Slud4446*
YBR186W	***PCH2***																						*Slud4234*
YDL102W	***POL3*** (*CDC2*, *HPR6*, *TEX1*)																						*Slud0767*
YLR376C	***PSY3***																						*Slud5095*
YPL022W	***RAD1*** (*RAD12*, *LPB9*)																						*Slud4308*
YGL058W	***RAD6*** (*UBC2*, *PSO8*)																						*Slud3390*
YML095C	***RAD10***																						*Slud3689*
YOR368W	***RAD17***																						*Slud3219*
YER173W	***RAD24*** (*RS1*)																						*Slud3925*
YNL250W	***RAD50***																						*Slud2677*
YER095W	***RAD51*** (*MUT5*)																						*Slud2123*
YML032C	***RAD52***																						*Slud3198*
YGL163C	***RAD54*** (*XRS1*)																						*Slud3267*
YDR076W	***RAD55***																						*Slud3886*
YDR004W	***RAD57***																						*Slud0658*
YDL059C	***RAD59***																						*Slud4914*
YBR073W	***RDH54*** (*TID1*)																						*Slud1662*
YLR329W	***REC102***																						*Slud0231*
YHR157W	***REC104***																						*Slud0173*
YJR021C	***REC107*** (*MER2*)																						*Slud2929*
YMR133W	***REC114***																						*Slud0747*
YPR007C	***REC8*** (*SPO69*)																						*Slud3262*
YAR007C	***RFA1*** (*RPA70*, *BUF2*, *FUN3*, *RPA1*)																						*Slud3825*
YNL312W	***RFA2*** (*RPA32*, *RPA2*, *BUF1*)																						*Slud4424*
YJL173C	***RFA3*** (*RPA14*, *RPA3*)																						*Slud2650*
YHL024W	***RIM4***																						*Slud3708*
YPL024W	***RMI1*** (*NCE4*)																						*Slud4306*
YGL250W	***RMR1***																						*Slud0436*
YGL175C	***SAE2*** (*COM1*)																						*Slud3748*
YHR079C-A	***SAE3***																						*Slud0413*
YAL027W	***SAW1***																						*Slud1916*
YHL006C	***SHU1***																						*Slud2532*
YDR078C	***SHU2***																						*Slud3889*
YGL213C	***SKI8*** (*REC103*)																						*Slud0482*
YBR228W	***SLX1***																						*Slud3405*
YLR135W	***SLX4***																						*Slud4009*
YJL074C	***SMC3***																						*Slud1559*
YOL034W	***SMC5***																						*Slud1891*
YLR383W	***SMC6*** (*RHC18*)																						*Slud2429*
YGL127C	***SOH1*** (*MED31*)																						*Slud3396*

YHL022C	***SPO11***	*Slud0631*
YJL092W	***SRS2*** *(HPR5, RADH, RADH1)*	*Slud1628*
YNL088W	***TOP2*** *(TOR3, TRF3)*	*Slud1001*
YLR234W	***TOP3*** *(EDR1)*	*Slud4360*
YDR369C	***XRS2***	*Slud1207*
YER041W	***YEN1***	*Slud0470*
YDR285W	***ZIP1***	*Slud2848*
YGL249W	***ZIP2***	*Slud0437*
**Regulation**		
YBL097W	***BRN1***	*Slud2171*
YPL178W	***CBC2*** *(CBP20, MUD13, SAE1)*	*Slud3448*
YDL017W	***CDC7*** *(LSD6, SAS1)*	*Slud0551*
YPL200W | YGL075C	***CSM4*** | ***MPS2*** *(MMC1)*	*Slud3503*
YDR110W	***FOB1*** *(HRM1)*	*Slud3944*
YDR318W	***MCM21*** *(CTF5)*	*Slud2860*
YBR098W	***MMS4*** *(SLX2)*	*Slud0400*
YHR086W	***NAM8*** *(MRE2, MUD15)*	*Slud3149*
YOL104C	***NDJ1*** *(TAM1)*	*Slud0702*
YNL330C	***RPD3*** *(REC3, SDI2, SDS6, MOF6)*	*Slud4400*
YHR119W	***SET1*** *(YTX1, KMT2)*	*Slud0958*
YJL168C	***SET2*** *(EZL1, KMT3)*	*Slud2644*
YMR190C	***SGS1***	*Slud3569*
YDL042C | YOL068C	***SIR2*** *(MAR1)* | ***HST1***	*Slud4467*
YLR135W	***SLX4***	*Slud4009*
YFR031C	***SMC2***	*Slud0612*
YHR153C	***SPO16***	*Slud0172*
YPL138C	***SPP1*** *(CPS40, SAF41)*	*Slud4147*
YJL092W	***SRS2*** *(HPR5, RADH, RADH1)*	*Slud1628*
YBR175W	***SWD3*** *(CPS30, SAF35)*	*Slud4209*
YLR182W	***SWI6*** *(PSL8, SDS11)*	*Slud2726*
YBL088C	***TEL1***	*Slud2160*
YDR325W	***YCG1*** *(TIE1, YCS5)*	*Slud3023*
YLR272C	***YCS4*** *(LOC7)*	*Slud3316*
YDR285W	***ZIP1***	*Slud2848*
**Checkpoint**		
YPL194W	***DDC1***	*Slud3495*
YDR440W	***DOT1*** *(KMT4, PCH1)*	*Slud1313*
YML074C | YLR449W	***FPR3*** *(NPI46)* | ***FPR4***	*Slud2332*
YIL072W	***HOP1***	*Slud2047*
YLR288C	***MEC3*** *(PSO9, PIP3)*	*Slud3303*
YOR351C	***MEK1*** *(MRE4)*	*Slud1340*
YBR186W	***PCH2***	*Slud4234*
YDR075W	***PPH3***	*Slud3885*
YNL201C	***PSY2***	*Slud1053*
YLR263W	***RED1***	*Slud3329*
YDL042C | YOL068C	***SIR2*** *(MAR1)* | ***HST1***	*Slud4467*
**Non-homologous end joining (NHEJ)**		
YOR005C	***DNL4*** *(LIG4)*	-
YKL213C	***DOA1*** *(UFD3, ZZZ4)*	*Slud4056*
YNL133C	***FYV6***	*Slud0943*
YLR247C	***IRC20***	*Slud1454*
YGL090W	***LIF1***	-
YNL307C | YOL128C	***MCK1*** *(CMS1, YPK1)* | ***YGK3***	*Slud4432*
YMR224C	***MRE11*** *(RAD58, XRS4, NGS1)*	*Slud1978*
YLR265C	***NEJ1*** *(LIF2)*	-
YNL102W	***POL1*** *(CDC17, CRT5, HPR3)*	*Slud0982*
YNL262W	***POL2*** *(DUN2)*	*Slud2693*
YCR014C	***POL4*** *(POLX)*	*Slud1499*
YMR137C	***PSO2*** *(SNM1)*	*Slud0751*
YKL113C	***RAD27*** *(ERC11, RTH1, FEN1)*	*Slud2518*
YNL250W	***RAD50***	*Slud2677*
YGR056W | YLR357W	***RSC1*** | ***RSC2***	*Slud3252*
YHR056C | YDR303C	***RSC30*** | ***RSC3***	*Slud3345* | *Slud2818*
YFR037C	***RSC8*** *(SWH3)*	*Slud1990*
YOL004W	***SIN3*** *(CPE1, GAM2, RPD1, SDI1, SDS16, UME4)*	*Slud3355*
YJL092W	***SRS2*** *(HPR5, RADH, RADH1)*	*Slud1628*
YMR039C	***SUB1*** *(TSP1)*	*Slud2505*
YNL246W	***VPS75***	*Slud2672*
YDR369C	***XRS2***	*Slud1207*
YMR284W	***YKU70*** *(HDF1, NES24, KU70)*	*Slud3643*
YMR106C	***YKU80*** *(HDF2)*	*Slud2596*
**Meiotic cell cycle - nuclear division**		
**DNA replication and integrity checkpoint**		
YGR225W	***AMA1*** *(SPO70)*	*Slud2969*
YDL017W	***CDC7*** *(LSD6, SAS1)*	*Slud0551*
YJR057W	***CDC8***	*Slud1952*
YOR074C	***CDC21*** *(CRT9, TMP1)*	*Slud1134*
YPR120C | YGR109C	***CLB5*** | ***CLB6***	*Slud0494*
YDR052C	***DBF4*** *(LSD7, DNA52)*	*Slud1671*
YDR359C	***EAF1*** *(VID21)*	*Slud0372*
YPL055C	***LGE1***	*Slud4278*
YBR057C	***MUM2*** *(SPOT8)*	*Slud1644*
YNL102W	***POL1*** *(CDC17, CRT5, HPR3)*	*Slud0982*
YDL102W	***POL3*** *(CDC2, HPR6, TEX1)*	*Slud0767*
YKL045W	***PRI2***	*Slud2467*
YHL024W	***RIM4***	*Slud3708*

YAL009W	***SPO7***	*Slud1890*
**Sister chromatid cohesion**		
YDR254W	***CHL4*** *(CTF17, MCM17)*	*Slud4292*
YPR135W	***CTF4*** *(CHL15, POB1)*	*Slud4595*
YFR027W	***ECO1*** *(CTF7)*	*Slud1470*
YBR107C	***IML3*** *(MCM19)*	*Slud0081*
YIL026C	***IRR1*** *(SCC3)*	*Slud0598*
YER106W	***MAM1***	*Slud2143*
YDL003W	***MCD1*** *(PDS3, RHC21, SCC1)*	*Slud0672*
YJL019W	***MPS3*** *(NEP98, YJL018W)*	*Slud2950*
YOL104C	***NDJ1*** *(TAM1)*	*Slud0702*
YDR014W	***RAD61*** *(WPL1)*	*Slud1592*
YPR007C	***REC8*** *(SPO69)*	*Slud3262*
YOR014W	***RTS1*** *(SCS1)*	*Slud1889*
YDR180W	***SCC2***	*Slud4388*
YER147C	***SCC4***	*Slud3882*
YOR073W	***SGO1***	*Slud1135*
YFL008W	***SMC1*** *(CHL10)*	*Slud1736*
YJL074C	***SMC3***	*Slud1559*
YHR014W	***SPO13***	*Slud0183*
**Synapsis - synaptonemal complex**		
YGR225W	***AMA1*** *(SPO70)*	*Slud2969*
YBR160W	***CDC28*** *(CDK1, HSL5, SRM5)*	*Slud4280*
YMR001C	***CDC5*** *(MSD2, PKX2)*	*Slud3353*
YPL200W | YGL075C	***CSM4*** | ***MPS2*** *(MMC1)*	*Slud3503*
YLR394W	***CST9*** *(ZIP3)*	*Slud2415*
YKL054C	***DEF1*** *(VID31)*	*Slud2487*
YER179W	***DMC1*** *(ISC2)*	*Slud3934*
YDR446W	***ECM11***	*Slud3211*
YDR506C	***GMC1***	-
YLR445W	***GMC2***	*Slud2339*
YDR014W-A	***HED1***	*Slud0064*
YGL251C	***HFM1*** *(MER3)*	*Slud0435*
YIL072W	***HOP1***	*Slud2047*
YGL033W	***HOP2***	*Slud3835*
YMR167W	***MLH1*** *(PMS2)*	*Slud0795*
YPL164C	***MLH3***	*Slud4111*
YJL019W	***MPS3*** *(NEP98, YJL018W)*	*Slud2950*
YOL104C	***NDJ1*** *(TAM1)*	*Slud0702*
YMR076C	***PDS5***	*Slud4611*
YPR007C	***REC8*** *(SPO69)*	*Slud3262*
YLR263W	***RED1***	*Slud3329*
YFR031C	***SMC2***	*Slud0612*
YJL074C	***SMC3***	*Slud1559*
YLR086W	***SMC4***	*Slud3941*
YHR153C	***SPO16***	*Slud0172*
YIL073C	***SPO22*** *(ZIP4)*	*Slud2064*
YNL088W	***TOP2*** *(TOR3, TRF3)*	*Slud1001*
YDL064W	***UBC9***	*Slud2421*
YDR325W	***YCG1*** *(TIE1, YCS5)*	*Slud3023*
YLR272C	***YCS4*** *(LOC7)*	*Slud3316*
YDR285W	***ZIP1***	*Slud2848*
YGL249W	***ZIP2***	*Slud0437*
**Mismatch repair**		
YMR167W	***MLH1*** *(PMS2)*	*Slud0795*
YPL164C	***MLH3***	*Slud4111*
YOL090W	***MSH2*** *(PMS5)*	*Slud4489*
YCR092C	***MSH3***	*Slud1711*
YDR097C	***MSH6*** *(PMS3, PMS6)*	*Slud3921*
YNL082W	***PMS1***	*Slud0912*
YBR088C	***POL30*** *(PCNA)*	*Slud4693*
YPL022W	***RAD1*** *(RAD12, LPB9)*	*Slud4308*
YML095C	***RAD10***	*Slud3689*
**Telomere clustering**		
YPL200W | YGL075C	***CSM4*** | ***MPS2*** *(MMC1)*	*Slud3503*
YJL019W	***MPS3*** *(NEP98, YJL018W)*	*Slud2950*
YOL104C	***NDJ1*** *(TAM1)*	*Slud0702*
YJL080C	***SCP160***	*Slud1562*
**Chromosome segregation**		
YGL071W | YPL202C	***AFT1*** *(RCS1)* | ***AFT2***	*Slud3505*
YGR225W	***AMA1*** *(SPO70)*	*Slud2969*
YPL204W	***HRR25*** *(KTI14)*	*Slud3509*
YGL190C	***CDC55*** *(TMR4)*	*Slud3734*
YFR046C	***CNN1***	-
YCR086W	***CSM1***	*Slud5099*
YIL132C	***CSM2***	*Slud0898*
YMR048W	***CSM3***	*Slud2513*
YPL200W | YGL075C	***CSM4*** | ***MPS2*** *(MMC1)*	*Slud3503*
YPL018W	***CTF19*** *(MCM18)*	*Slud1744*
YDR110W	***FOB1*** *(HRM1)*	*Slud3944*
YPL209C	***IPL1*** *(PAC15)*	*Slud3515*
YDR439W	***LRS4***	*Slud1310*
YGR188C | YJL013C	***BUB1*** | ***MAD3***	*Slud2952*
YGL086W	***MAD1***	*Slud3478*
YJL030W	***MAD2***	*Slud3074*
YER106W	***MAM1***	*Slud2143*

YDR383C	***NKP1***	-
YLR315W	***NKP2***	-
YOR373W	***NUD1***	*Slud3224*
YMR190C	***SGS1***	*Slud3569*
YFR031C	***SMC2***	*Slud0612*
YLR086W	***SMC4***	*Slud3941*
YML085C | YML124C	***TUB1*** | ***TUB3***	*Slud3671*
YFL037W	***TUB2*** (*ARM10*, *SHE8*)	*Slud1781*
YDR374W-A	***WIP1***	-
YDR325W	***YCG1*** (*TIE1*, *YCS5*)	*Slud3023*
YLR272C	***YCS4*** (*LOC7*)	*Slud3316*

Sister chromatid segregation

YPR135W	***CTF4*** (*CHL15*, *POB1*)	*Slud4595*
YIR010W	***DSN1***	*Slud0622*
YGR098C	***ESP1***	*Slud0504*
YBR107C	***IML3*** (*MCM19*)	*Slud0081*
YPL209C	***IPL1*** (*PAC15*)	*Slud3515*
YPL017C | YFL018C	***IRC15*** | ***LPD1*** (*HPD1*)	*Slud1739*
YLR320W	***MMS22*** (*SLM2*)	*Slud3792*
YBR073W	***RDH54*** (*TID1*)	*Slud1662*
YOR073W	***SGO1***	*Slud1135*
YGL127C	***SOH1*** (*MED31*)	*Slud3396*

Regulation

YKL112W	***ABF1*** (*SBF-B*, *SBF1*, *BAF1*, *OBF1*, *REB2*, *GFI*)	*Slud2550*
YIL033C	***BCY1*** (*SRA1*)	*Slud1992*
YMR001C	***CDC5*** (*MSD2*, *PKX2*)	*Slud3353*
YDL017W	***CDC7*** (*LSD6*, *SAS1*)	*Slud0551*
YGL116W	***CDC20*** (*PAC5*)	*Slud3415*
YLR310C | YLL016W | YLL017W	***CDC25*** (*CDC25'*, *CTN1*) | ***SDC25*** | **-**	*Slud3765*
YBR160W	***CDC28*** (*CDK1*, *HSL5*, *SRM5*)	*Slud4280*
YGR108W | YPR119W	***CLB1*** (*SCB1*) | ***CLB2***	*Slud0493*
YAL040C	***CLN3*** (*DAF1*, *FUN10*, *WHI1*)	*Slud1335* | *Slud2710*
YDR155C	***CPR1*** (*CYP1*, *CPH1*)	*Slud4015*
YPL200W | YGL075C	***CSM4*** | ***MPS2*** (*MMC1*)	*Slud3503*
YBR112C	***CYC8*** (*CRT8*, *SSN6*, [*OCT1*$^{+}$], [*OCT*])	*Slud1666* | *Slud1828*
YDR052C	***DBF4*** (*LSD7*, *DNA52*)	*Slud1671*
YKL054C	***DEF1*** (*VID31*)	*Slud2487*
YER020W	***GPA2*** (SSP101)	*Slud2231*
YFL031W	***HAC1*** (*ERN4*, *IRE15*)	*Slud1802*
YGL194C	***HOS2*** (*RTL1*)	*Slud3731*
YIL112W	***HOS4***	*Slud0873*
YJL146W	***IDS2***	*Slud0725*
YJR094C	***IME1***	*Slud2202*
YJL106W	***IME2*** (*SME1*)	*Slud1609*
YGL192W	***IME4*** (*SPO8*)	*Slud3732*
YOR304W	***ISW2***	*Slud3230*
YGL133W | YPL216W	***ITC1*** | **-**	*Slud3526*
YGL197W | YER132C	***MDS3*** | ***PMD1***	*Slud3727*
YMR037C | YKL062W	***MSN2*** | ***MSN4***	*Slud2501*
YMR224C	***MRE11*** (*RAD58*, *XRS4*, *NGS1*)	*Slud1978*
YOL104C	***NDJ1*** (*TAM1*)	*Slud0702*
YHR124W	***NDT80***	*Slud0944*
YNL231C	***PDR16*** (*SFH3*)	*Slud1082*
YDR113C	***PDS1***	*Slud3947*
YOR208W	***PTP2***	*Slud4164*
YER075C	***PTP3***	*Slud2043*
YNL098C | YOR101W	***RAS2*** (*CTN5*, *CYR3*, *TSL7*, *GLC5*) | ***RAS1***	*Slud0985*
YLR248W | YGL158W	***RCK2*** (*CLK1*, *CMK3*) | ***RCK1***	*Slud1455*
YLR071C	***RGR1*** (*MED14*)	*Slud3277*
YCR028C-A	***RIM1***	*Slud2661*
YGL045W	***RIM8*** (*ART9*, *PAL3*, *YGL046W*)	*Slud3846*
YMR063W	***RIM9***	*Slud1119*
YMR139W | YDL079C	***RIM11*** (*GSK3*, *MDS1*) | ***MRK1***	*Slud0754*
YMR154C	***RIM13*** (*CPL1*)	*Slud0780*
YFL033C	***RIM15*** (*TAK1*)	*Slud1774*
YOR275C	***RIM20***	*Slud4075*
YGR044C	***RME1*** (*CSP1*)	*Slud3260*
YBL093C	***ROX3*** (*MED19*, *SSN7*, *SSX2*, *NUT3*)	*Slud2167*
YNL330C	***RPD3*** (*REC3*, *SDI2*, *SDS6*, *MOF6*)	*Slud4400*
YFL034C-A | YLR061W	***RPL22B*** (*eL22*, *YL31*, *l1c*, *L22B*, *rp4*, *L22e*) | ***RPL22A*** (*L22A*)	*Slud1777*
YKR029C | YJL105W	***SET3*** | ***SET4***	*Slud1610*
YBR103W	***SIF2*** (*EMB1*)	*Slud4733*
YOL004W	***SIN3*** (*CPE1*, *GAM2*, *RPD1*, *SDI1*, *SDS16*, *UME4*)	*Slud3355*
YNL236W	***SIN4*** (*MED16*, *RYE1*, *BEL2*, *GAL22*, *SDI3*, *SSF5*, *SSN4*, *TSF3*, *SSX3*)	*Slud1085*
YDR510W	***SMT3***	*Slud2733*
YDR477W	***SNF1*** (*CAT1*, *CCR1*, *GLC2*, *HAF3*, *PAS14*)	*Slud3165*
YDL194W | YDL138W	***SNF3*** | ***RGT2***	*Slud1425*
YMR016C | YKL043W	***SOK2*** | ***PHD1***	*Slud2466*
YHR152W | YGR230W	***SPO12*** (*SDB21*) | ***BNS1***	-
YDR443C	***SSN2*** (*RYE3*, *MED13*, *NUT8*, *SCA1*, *SRB9*, *UME2*, *SSX5*)	*Slud1316*
YNL025C	***SSN8*** (*RYE2*, *CycC*, *GIG3*, *NUT9*, *SRB11*, *UME3*, *CNC1*)	*Slud0854*
YMR039C	***SUB1*** (*TSP1*)	*Slud2505*
YDR310C	***SUM1***	*Slud2808*
YPL016W	***SWI1*** ([*SWI*$^{+}$], [*SWI*(+)], *ADR6*, *GAM3*, *LPA1*)	*Slud4309*
YJL187C	***SWE1*** (*WEE1*)	*Slud2663*
YPL157W	***TGS1***	*Slud4119*
YJL164C | YKL166C	***TPK1*** (*SRA3*, *PKA1*) | ***TPK3***	*Slud0567*
YPL203W	***TPK2*** (*PKA3*, *PKA2*, *YKR1*)	*Slud3508*

YCR084C	***TUP1*** *(AAR1, AER2, AMM1, CRT4, CYC9, FLK1, ROX4, SFL2, UMR7)*		*Slud1704*
YPL139C | YOR229W	***UME1*** *(WTM3)* | ***WTM2***		*Slud4146*
YDR207C	***UME6*** *(CAR80, NIM2, RIM16)*		*Slud4358*
YOR230W	***WTM1***		-
**Others**			
YJL115W	***ASF1*** *(CIA1)*		*Slud4497*
YFR021W	***ATG18*** *(SVP1, NMR1, CVT18, AUT10)*		*Slud1442*
YJR053W	***BFA1*** *(IBD1)*		*Slud1943*
YMR055C	***BUB2*** *(PAC7)*		*Slud4833*
YGL174W	***BUD13*** *(CWC26)*		*Slud3747*
YLR103C	***CDC45*** *(SLD4)*		*Slud3969*
YJL005W	***CYR1*** *(FIL1, CDC35, HSR1, SRA4, TSM0185)*		*Slud2922*
YAL013W	***DEP1*** *(FUN54)*		*Slud1898*
YNL001W | YCL001W-B	***DOM34*** | -		*Slud1394*
YPR175W	***DPB2***		*Slud2265*
YDL101C	***DUN1***		*Slud5068*
YOL017W | YFR013W	***ESC8*** | ***IOC3***		-
YGR252W	***GCN5*** *(ADA4, SWI9, KAT2, AAS104)*		*Slud3052*
YER133W	***GLC7*** *(CID1, DIS2, PP1, DIS2S1)*		*Slud3725*
YCR065W	***HCM1***		*Slud3615*
YDR138W	***HPR1*** *(TRF1)*		*Slud3982*
YIR005W	***IST3*** *(SNU17)*		*Slud4552*
YOL086W-A	***MHF1***		*Slud0285*
YDL160C-A	***MHF2***		*Slud0411*
YCL061C	***MRC1***		*Slud2773*
YEL062W	***NPR2***		*Slud4900*
YHL023C	***NPR3*** *(RMD11)*		*Slud3707*
YBR233W	***PBP2*** *(HEK1)*		*Slud3412*
YNL267W	***PIK1*** *(PIK41, PIK120)*		*Slud2704*
YLR016C	***PML1***		*Slud0177*
YGL167C	***PMR1*** *(SSC1, BSD1, LDB1)*		*Slud1794*
YDR217C	***RAD9***		*Slud4349*
YNL072W	***RNH201*** *(RNH35, Rnh2A)*		*Slud0902*
YER070W | YIL066C	***RNR1*** *(CRT7, RIR1, SDS12)* | ***RNR3*** *(DIN1, RIR3)*		*Slud2036*
YGL066W	***SGF73*** *(SCA7)*		*Slud3511*
YLR442C | YML065W	***SIR3*** *(CMT1, MAR2, STE8)* | ***ORC1***		*Slud2354*
YDR240C	***SNU56*** *(MUD10)*		*Slud4316*
YKR031C	***SPO14*** *(PLD1)*		*Slud1606*
YMR179W	***SPT21***		*Slud3544*
YDR108W	***TRS85*** *(GSG1, MUM1)*		*Slud0393*

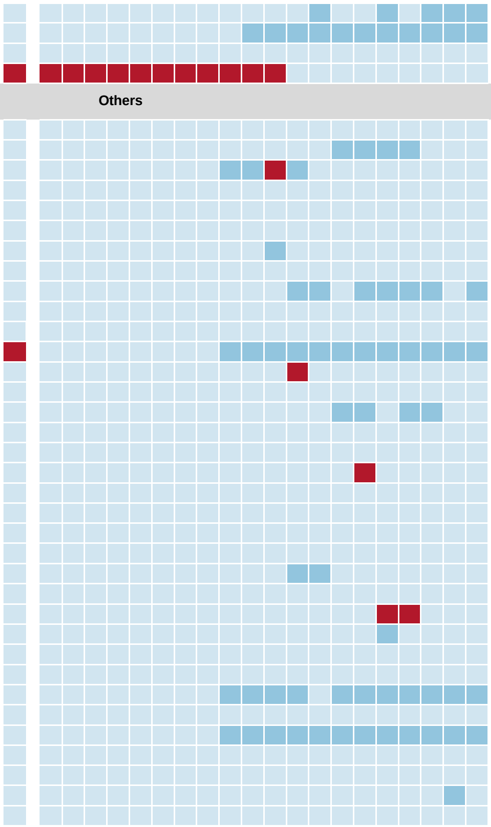

present (one homolog)
present (at least two paralogs)
presence of syntenic ORF with no or very weak similarity to the *S. cerevisiae* homolog
absent
