# Supplementary material for: Sex without crossing over in the yeast Saccharomycodes ludwigii
Source: Genome Biol. 2021 Nov 3;22:303. doi: 10.1186/s13059-021-02521-w (PMC8567612; doi:10.1186/s13059-021-02521-w)
Supplement: Supplementary file 5 — Additional file 5: Table S4. Population analyses: read mapping statistics. [file 13059_2021_2521_MOESM5_ESM.pdf]

Table S4 Population analysis - read mapping statistics

	Ploidy estimation (flow cytometry)	Mapping on reference genome			Mapping on masked reference genome			Mapping on masked reference genome (subtelomeres removed)		
**Strain**	**Ploidy**	**# SNPs**	**# homozygous SNPs**	**# heterozygous SNPs**	**# SNPs**	**# homozygous SNPs**	**# heterozygous SNPs**	**# SNPs**	**# homozygous SNPs**	**# heterozygous SNPs**
CBS 1168	haploid	41,533	35,052	6,481	32,832	26,991	5,841	27,466	25,455	2,011
PC99_R_1	haploid	443,046	435,913	7,133	318,604	312,832	5,772	311,830	308,351	3,479
122	haploid	204,068	197,741	6,327	149,054	142,238	6,816	144,655	140,038	4,617
UTAD17	haploid[1]	31,189	24,704	6,485	25,026	19,182	5,844	21,628	18,360	3,268
NCYC 732	diploid	31,867	27,474	4,393	24,924	21,155	3,769	21,360	20,065	1,295
NCYC 734	diploid	42,188	35,306	6,882	33,419	27,449	5,970	27,588	25,948	1,640
NCYC 3345	aneuploid (2n+1)[2]	37,319	20,655	16,664	29,335	15,791	13,544	26,252	15,151	11,101
BJK-5C	diploid	46,611	25,013	21,598	37,367	19,283	18,084	33,375	18,268	15,107
NCYC 849	diploid	40,172	17,423	22,749	31,809	13,309	18,500	28,065	12,879	15,186
DSM 70550	aneuploid (2n+1)[3]	115,551	28,924	86,627	86,940	22,559	64,381	80,351	20,553	59,798

[1] The isolate was not available in our study, but it is presumed to be haploid (GenBank assembly accession: GCA_900491785.1).

[2] Based on read coverage, the isolate was determined to have an additional (third) copy of chromosome D.

[3] Based on read coverage, the isolate was determined to have an additional (third) copy of chromosome G.
