# Supplementary material for: Sex without crossing over in the yeast Saccharomycodes ludwigii
Source: Genome Biol. 2021 Nov 3;22:303. doi: 10.1186/s13059-021-02521-w (PMC8567612; doi:10.1186/s13059-021-02521-w)
Supplement: Supplementary file 6 — Additional file 6: Table S5. Novel mutations in the mutation accumulation experiment. [file 13059_2021_2521_MOESM6_ESM.pdf]

Table S5 Novel mutations that arose during the mutation accumulation experiment

strain	ploidy	chr	pos	line	AC	AB	GQ	REF	ALT	GT	note
NBRC 1722	haploid	chrA	400307	Sdl-C3	1	.	99	T	G	1	
NBRC 1722	haploid	chrA	666817	Sdl-D6	1	.	99	T	C	1	
NBRC 1722	haploid	chrA	674943	Sdl-A3	1	.	99	C	T	1	
NBRC 1722	haploid	chrA	683073	Sdl-B2	1	.	99	G	C	1	
NBRC 1722	haploid	chrA	781918	Sdl-A4	1	.	99	G	A	1	
NBRC 1722	haploid	chrA	920700	Sdl-C5	1	.	99	T	G	1	
NBRC 1722	haploid	chrA	1082934	Sdl-C2	1	.	99	C	T	1	
NBRC 1722	haploid	chrA	1114570	Sdl-B5	1	.	99	C	T	1	
NBRC 1722	haploid	chrA	1388818	Sdl-E3	1	.	99	C	A	1	
NBRC 1722	haploid	chrA	1391835	Sdl-B5	1	.	99	T	C	1	
NBRC 1722	haploid	chrA	1503698	Sdl-A4	1	.	99	T	G	1	
NBRC 1722	haploid	chrA	1676440	Sdl-A4	1	.	99	C	A	1	
NBRC 1722	haploid	chrA	1738809	Sdl-A3	1	.	99	A	G	1	
NBRC 1722	haploid	chrA	1798149	Sdl-D4	1	.	99	G	A	1	
NBRC 1722	haploid	chrA	1912929	Sdl-C2	1	.	99	A	G	1	
NBRC 1722	haploid	chrA	2096503	Sdl-C1	1	.	99	C	A	1	
NBRC 1722	haploid	chrA	2246284	Sdl-A6	1	.	99	C	A	1	
NBRC 1722	haploid	chrA	2254515	Sdl-D6	1	.	99	A	C	1	
NBRC 1722	haploid	chrA	2283408	Sdl-E3	1	.	99	A	G	1	
NBRC 1722	haploid	chrA	2776172	Sdl-E1	1	.	99	C	A	1	
NBRC 1722	haploid	chrA	2787652	Sdl-D4	1	.	99	G	T	1	
NBRC 1722	haploid	chrA	3063321	Sdl-E3	1	.	99	T	G	1	
NBRC 1722	haploid	chrB	10675	Sdl-B6	1	.	99	A	C	1	
NBRC 1722	haploid	chrB	260595	Sdl-E5	1	.	99	C	A	1	
NBRC 1722	haploid	chrB	376935	Sdl-E1	1	.	99	A	T	1	
NBRC 1722	haploid	chrB	541689	Sdl-E5	1	.	99	G	T	1	
NBRC 1722	haploid	chrB	627968	Sdl-B2	30	.	15	C	T	0	
NBRC 1722	haploid	chrB	954927	Sdl-A3	1	.	99	C	G	1	
NBRC 1722	haploid	chrB	1067204	Sdl-D4	1	.	99	T	C	1	
NBRC 1722	haploid	chrB	1482284	Sdl-A6	1	.	99	C	T	1	
NBRC 1722	haploid	chrB	2169189	Sdl-D4	1	.	99	A	C	1	
NBRC 1722	haploid	chrC	401275	Sdl-D1	1	.	99	G	A	1	
NBRC 1722	haploid	chrC	588395	Sdl-B2	1	.	99	C	T	1	
NBRC 1722	haploid	chrC	735592	Sdl-C4	1	.	99	G	A	1	
NBRC 1722	haploid	chrC	802357	Sdl-A4	1	.	99	G	T	1	
NBRC 1722	haploid	chrC	971450	Sdl-A6	1	.	99	T	A	1	
NBRC 1722	haploid	chrC	1131055	Sdl-A6	1	.	99	A	T	1	
NBRC 1722	haploid	chrC	1199715	Sdl-A1	1	.	99	A	G	1	
NBRC 1722	haploid	chrC	1345913	Sdl-C2	1	.	99	A	G	1	
NBRC 1722	haploid	chrC	1522911	Sdl-B5	1	.	99	C	G	1	
NBRC 1722	haploid	chrC	1604135	Sdl-A3	1	.	99	C	T	1	
NBRC 1722	haploid	chrC	1694824	Sdl-E2	1	.	99	G	A	1	
NBRC 1722	haploid	chrC	1706719	Sdl-C1	30	.	47	G	A	0	
NBRC 1722	haploid	chrD	6751	Sdl-B1	1	.	99	A	T	1	
NBRC 1722	haploid	chrD	145289	Sdl-D5	1	.	99	G	T	1	
NBRC 1722	haploid	chrD	260253	Sdl-C4	1	.	99	G	T	1	
NBRC 1722	haploid	chrD	369054	Sdl-B3	1	.	99	T	C	1	
NBRC 1722	haploid	chrD	419232	Sdl-A4	1	.	99	T	A	1	
NBRC 1722	haploid	chrD	483354	Sdl-C6	1	.	99	T	A	1	
NBRC 1722	haploid	chrD	665188	Sdl-E1	1	.	99	A	T	1	
NBRC 1722	haploid	chrD	743085	Sdl-C6	1	.	99	A	G	1	
NBRC 1722	haploid	chrD	903666	Sdl-D3	1	.	99	A	G	1	
NBRC 1722	haploid	chrD	1332088	Sdl-A6	1	.	99	C	T	1	
NBRC 1722	haploid	chrD	1608204	Sdl-C1	1	.	99	A	G	1	
NBRC 1722	haploid	chrD	1674136	Sdl-A6	1	.	99	G	T	1	
NBRC 1722	haploid	chrD	1674160	Sdl-A6	1	.	99	A	G	1	
NBRC 1722	haploid	chrD	1674161	Sdl-A6	1	.	99	T	C	1	
NBRC 1722	haploid	chrD	1674166	Sdl-A6	1	.	99	T	C	1	
NBRC 1722	haploid	chrD	1674168	Sdl-A6	1	.	99	A	G	1	
NBRC 1722	haploid	chrE	17379	Sdl-A6	1	.	99	C	G	1	
NBRC 1722	haploid	chrE	123437	Sdl-D5	1	.	99	C	T	1	
NBRC 1722	haploid	chrE	174769	Sdl-E2	1	.	99	G	A	1	

NBRC 1722	haploid	chrE	613893	Sdl-C1	1	.	99	A	G	1
NBRC 1722	haploid	chrE	719472	Sdl-E2	1	.	99	A	T	1
NBRC 1722	haploid	chrE	914233	Sdl-C1	1	.	99	C	A	1
NBRC 1722	haploid	chrE	1013120	Sdl-A6	1	.	99	C	T	1
NBRC 1722	haploid	chrE	1076816	Sdl-C5	1	.	99	G	A	1
NBRC 1722	haploid	chrE	1318818	Sdl-A3	1	.	99	T	C	1
NBRC 1722	haploid	chrE	1415009	Sdl-C4	30	.	75	T	G	0
NBRC 1722	haploid	chrF	180378	Sdl-D4	1	.	99	C	A	1
NBRC 1722	haploid	chrF	266998	Sdl-B2	1	.	99	A	G	1
NBRC 1722	haploid	chrF	510147	Sdl-D1	1	.	99	A	T	1
NBRC 1722	haploid	chrF	685831	Sdl-B5	1	.	99	T	C	1
NBRC 1722	haploid	chrF	704245	Sdl-C2	1	.	99	T	C	1
NBRC 1722	haploid	chrF	816501	Sdl-E3	1	.	99	C	T	1
NBRC 1722	haploid	chrF	825035	Sdl-C2	30	.	73	G	A	0
NBRC 1722	haploid	chrF	1294027	Sdl-C5	1	.	99	G	A	1
NBRC 1722	haploid	chrF	1294062	Sdl-C5	1	.	99	A	G	1
NBRC 1722	haploid	chrF	1301219	Sdl-A3	30	.	2	A	T	0
NBRC 1722	haploid	chrF	1307867	Sdl-B3	30	.	99	G	A	0
NBRC 1722	haploid	chrF	1307871	Sdl-B3	30	.	99	C	A	0
NBRC 1722	haploid	chrG	3489	Sdl-B6	30	.	18	T	C	0
NBRC 1722	haploid	chrG	233665	Sdl-C1	1	.	99	G	A	1
NBRC 1722	haploid	chrG	492229	Sdl-B1	1	.	99	G	C	1
NBRC 1722	haploid	chrG	618893	Sdl-E4	1	.	99	A	T	1
YLFP17-4	diploid (isogenic)	chrA	134295	J5	1	0.56	99	C	T	0/1
YLFP17-4	diploid (isogenic)	chrA	485187	F1	1	0.47	99	G	T	0/1
YLFP17-4	diploid (isogenic)	chrA	873994	F5	1	0.41	99	G	A	0/1
YLFP17-4	diploid (isogenic)	chrA	890571	G3	1	0.5	99	T	G	0/1
YLFP17-4	diploid (isogenic)	chrA	1300923	F5	1	0.45	99	C	T	0/1
YLFP17-4	diploid (isogenic)	chrA	1463165	H3	1	0.48	99	A	G	0/1
YLFP17-4	diploid (isogenic)	chrA	1640205	F1	1	0.43	99	T	C	0/1
YLFP17-4	diploid (isogenic)	chrA	1753949	G6	1	0.47	99	C	A	0/1
YLFP17-4	diploid (isogenic)	chrA	2043594	F4	1	0.58	99	C	T	0/1
YLFP17-4	diploid (isogenic)	chrA	2708859	F4	1	0.48	99	C	A	0/1
YLFP17-4	diploid (isogenic)	chrA	2711278	F4	1	0.43	99	C	A	0/1
YLFP17-4	diploid (isogenic)	chrA	2888473	I4	1	0.54	99	T	A	0/1
YLFP17-4	diploid (isogenic)	chrB	113493	G6	1	0.52	99	T	C	0/1
YLFP17-4	diploid (isogenic)	chrB	261445	J6	1	0.46	99	C	A	0/1
YLFP17-4	diploid (isogenic)	chrB	785254	F6	1	0.42	99	T	C	0/1
YLFP17-4	diploid (isogenic)	chrB	1061405	F1	1	0.47	99	C	A	0/1
YLFP17-4	diploid (isogenic)	chrB	1542769	I5	1	0.57	99	G	T	0/1
YLFP17-4	diploid (isogenic)	chrB	1610644	J4	1	0.44	99	A	G	0/1
YLFP17-4	diploid (isogenic)	chrB	2198465	I4	1	0.55	99	A	G	0/1
YLFP17-4	diploid (isogenic)	chrC	87097	F3	1	0.53	99	A	G	0/1
YLFP17-4	diploid (isogenic)	chrC	460750	J3	1	0.59	99	G	T	0/1
YLFP17-4	diploid (isogenic)	chrC	501290	J5	1	0.54	99	T	C	0/1
YLFP17-4	diploid (isogenic)	chrC	671979	I4	1	0.47	99	A	G	0/1
YLFP17-4	diploid (isogenic)	chrC	883032	F4	1	0.55	99	G	T	0/1
YLFP17-4	diploid (isogenic)	chrC	952114	J2	1	0.52	99	C	T	0/1
YLFP17-4	diploid (isogenic)	chrC	993017	J2	1	0.43	99	A	G	0/1
YLFP17-4	diploid (isogenic)	chrC	1078748	I2	1	0.53	99	G	T	0/1
YLFP17-4	diploid (isogenic)	chrC	1274107	I6	1	0.59	99	G	T	0/1
YLFP17-4	diploid (isogenic)	chrC	1545770	I6	1	0.43	99	G	A	0/1
YLFP17-4	diploid (isogenic)	chrC	1631738	J1	1	0.58	99	C	G	0/1
YLFP17-4	diploid (isogenic)	chrC	1795088	I2	1	0.56	99	C	T	0/1
YLFP17-4	diploid (isogenic)	chrC	1812850	G3	1	0.49	99	T	C	0/1
YLFP17-4	diploid (isogenic)	chrD	275057	I2	1	0.43	99	G	A	0/1
YLFP17-4	diploid (isogenic)	chrD	583701	G5	1	0.54	99	C	A	0/1
YLFP17-4	diploid (isogenic)	chrD	879177	G5	1	0.58	99	C	T	0/1
YLFP17-4	diploid (isogenic)	chrD	1022697	G6	1	0.55	99	A	G	0/1
YLFP17-4	diploid (isogenic)	chrD	1069206	H3	1	0.57	99	C	A	0/1
YLFP17-4	diploid (isogenic)	chrD	1136164	J4	1	0.55	99	G	T	0/1
YLFP17-4	diploid (isogenic)	chrD	1299741	I5	1	0.57	99	G	A	0/1
YLFP17-4	diploid (isogenic)	chrE	1334698	I2	1	0.43	99	C	T	0/1
YLFP17-4	diploid (isogenic)	chrF	144834	F5	1	0.51	99	C	A	0/1
YLFP17-4	diploid (isogenic)	chrF	223367	J3	1	0.53	99	G	A	0/1
YLFP17-4	diploid (isogenic)	chrF	364390	F6	1	0.57	99	G	A	0/1

YLFP17-4	diploid (isogenic)	chrF	974087	J1	1	0.41	99	G	A	0/1	
YLFP17-4	diploid (isogenic)	chrG	230046	F5	1	0.5	99	C	T	0/1	
YLFP17-4	diploid (isogenic)	chrG	230055	F5	1	0.5	99	T	A	0/1	
YLFP17-4	diploid (isogenic)	chrG	425497	F1	1	0.55	99	G	A	0/1	
YLFP17-4	diploid (isogenic)	chrG	634414	J5	1	0.46	99	C	T	0/1	
YLFP188-1	diploid (hybrid)	chrA	575511	K3	1	0.55	99	C	T	0/1	homozygous-to-homozygous substitution
YLFP188-1	diploid (hybrid)	chrA	595019	L3	1	0.44	99	C	G	0/1	homozygous-to-homozygous substitution
YLFP188-1	diploid (hybrid)	chrA	798264	K1	1	0.51	99	T	A	0/1	homozygous-to-homozygous substitution
YLFP188-1	diploid (hybrid)	chrA	811318	L4	1	0.5	99	G	A	0/1	homozygous-to-homozygous substitution
YLFP188-1	diploid (hybrid)	chrA	939612	K2	1	0.52	99	C	T	0/1	homozygous-to-homozygous substitution
YLFP188-1	diploid (hybrid)	chrA	1035295	K6	1	0.47	99	A	G	0/1	homozygous-to-homozygous substitution
YLFP188-1	diploid (hybrid)	chrA	1245954	L4	1	0.5	99	T	G	0/1	homozygous-to-homozygous substitution
YLFP188-1	diploid (hybrid)	chrA	1353499	L1	1	0.53	99	G	T	0/1	homozygous-to-homozygous substitution
YLFP188-1	diploid (hybrid)	chrA	1850563	L6	1	0.56	99	A	G	0/1	homozygous-to-homozygous substitution
YLFP188-1	diploid (hybrid)	chrA	2059925	K6	1	0.51	99	C	A	0/1	homozygous-to-homozygous substitution
YLFP188-1	diploid (hybrid)	chrA	2622539	L3	1	0.46	99	C	A	0/1	homozygous-to-homozygous substitution
YLFP188-1	diploid (hybrid)	chrA	2695038	K1	1	0.42	99	C	A	0/1	homozygous-to-homozygous substitution
YLFP188-1	diploid (hybrid)	chrA	3058692	L3	1	0.48	99	G	A	0/1	homozygous-to-homozygous substitution
YLFP188-1	diploid (hybrid)	chrB	117858	L2	1	0.51	99	C	G	0/1	homozygous-to-homozygous substitution
YLFP188-1	diploid (hybrid)	chrB	159385	K3	1	0.48	99	T	C	0/1	homozygous-to-homozygous substitution
YLFP188-1	diploid (hybrid)	chrB	345488	L1	1	0.51	99	G	A	0/1	homozygous-to-homozygous substitution
YLFP188-1	diploid (hybrid)	chrB	345632	K4	1	0.49	99	T	C	0/1	homozygous-to-homozygous substitution
YLFP188-1	diploid (hybrid)	chrB	540836	L1	1	0.41	99	T	C	0/1	homozygous-to-homozygous substitution
YLFP188-1	diploid (hybrid)	chrB	748410	K4	1	0.48	99	A	T	0/1	homozygous-to-homozygous substitution
YLFP188-1	diploid (hybrid)	chrB	748411	K4	1	0.52	99	T	G	0/1	homozygous-to-homozygous substitution
YLFP188-1	diploid (hybrid)	chrB	762074	K5	1	0.5	99	G	C	0/1	homozygous-to-homozygous substitution
YLFP188-1	diploid (hybrid)	chrB	1272305	L2	23	0.49	8	C	G	0/1	homozygous-to-homozygous substitution
YLFP188-1	diploid (hybrid)	chrB	2101324	K4	1	0.41	99	T	A	0/1	homozygous-to-homozygous substitution
YLFP188-1	diploid (hybrid)	chrB	2128527	K2	1	0.52	99	C	A	0/1	homozygous-to-homozygous substitution
YLFP188-1	diploid (hybrid)	chrC	209877	K2	1	0.47	99	A	G	0/1	homozygous-to-homozygous substitution
YLFP188-1	diploid (hybrid)	chrC	285477	L4	1	0.47	99	C	G	0/1	homozygous-to-homozygous substitution
YLFP188-1	diploid (hybrid)	chrC	649869	K5	1	0.44	99	A	G	0/1	homozygous-to-homozygous substitution
YLFP188-1	diploid (hybrid)	chrC	655945	K5	1	0.44	99	A	G	0/1	homozygous-to-homozygous substitution
YLFP188-1	diploid (hybrid)	chrC	758503	K3	1	0.47	99	T	G	0/1	homozygous-to-homozygous substitution
YLFP188-1	diploid (hybrid)	chrC	758505	K3	1	0.51	99	T	G	0/1	homozygous-to-homozygous substitution
YLFP188-1	diploid (hybrid)	chrC	930063	K4	1	0.46	99	G	T	0/1	homozygous-to-homozygous substitution
YLFP188-1	diploid (hybrid)	chrC	1243241	L6	1	0.5	99	G	A	0/1	homozygous-to-homozygous substitution
YLFP188-1	diploid (hybrid)	chrC	1310575	K6	1	0.48	99	A	G	0/1	homozygous-to-homozygous substitution
YLFP188-1	diploid (hybrid)	chrC	1495479	L6	1	0.45	99	A	C	0/1	homozygous-to-homozygous substitution
YLFP188-1	diploid (hybrid)	chrC	1553527	K3	1	0.48	99	G	A	0/1	homozygous-to-homozygous substitution
YLFP188-1	diploid (hybrid)	chrD	538374	K5	1	0.51	99	T	A	0/1	homozygous-to-homozygous substitution
YLFP188-1	diploid (hybrid)	chrD	637341	L6	1	0.48	99	A	G	0/1	homozygous-to-homozygous substitution
YLFP188-1	diploid (hybrid)	chrD	793607	L1	1	0.47	99	A	G	0/1	homozygous-to-homozygous substitution
YLFP188-1	diploid (hybrid)	chrD	1662826	L3	1	0.59	9	C	T	0/1	homozygous-to-homozygous substitution
YLFP188-1	diploid (hybrid)	chrD	1669254	L3	1	0.57	99	C	T	0/1	homozygous-to-homozygous substitution
YLFP188-1	diploid (hybrid)	chrE	183754	L6	1	0.45	99	C	T	0/1	homozygous-to-homozygous substitution
YLFP188-1	diploid (hybrid)	chrE	362933	L6	1	0.54	99	T	A	0/1	homozygous-to-homozygous substitution
YLFP188-1	diploid (hybrid)	chrE	388157	L1	1	0.48	99	A	T	0/1	homozygous-to-homozygous substitution
YLFP188-1	diploid (hybrid)	chrE	397152	L1	1	0.43	99	G	T	0/1	homozygous-to-homozygous substitution
YLFP188-1	diploid (hybrid)	chrE	671942	L3	1	0.52	99	C	T	0/1	homozygous-to-homozygous substitution
YLFP188-1	diploid (hybrid)	chrE	854034	L2	1	0.45	99	T	C	0/1	homozygous-to-homozygous substitution
YLFP188-1	diploid (hybrid)	chrF	369673	K3	1	0.57	99	A	G	0/1	homozygous-to-homozygous substitution
YLFP188-1	diploid (hybrid)	chrF	369675	K3	1	0.59	99	G	A	0/1	homozygous-to-homozygous substitution
YLFP188-1	diploid (hybrid)	chrF	981864	K1	1	0.54	99	T	A	0/1	homozygous-to-homozygous substitution
YLFP188-1	diploid (hybrid)	chrG	296907	L4	1	0.54	99	C	A	0/1	homozygous-to-homozygous substitution
YLFP188-1	diploid (hybrid)	chrG	400567	L1	1	0.51	99	C	T	0/1	homozygous-to-homozygous substitution
YLFP188-1	diploid (hybrid)	chrG	466575	L3	1	0.58	99	A	C	0/1	homozygous-to-homozygous substitution
YLFP188-1	diploid (hybrid)	chrG	505967	K1	1	0.49	99	A	T	0/1	homozygous-to-homozygous substitution
