# Supplementary material for: Sex without crossing over in the yeast Saccharomycodes ludwigii
Source: Genome Biol. 2021 Nov 3;22:303. doi: 10.1186/s13059-021-02521-w (PMC8567612; doi:10.1186/s13059-021-02521-w)
Supplement: Supplementary file 7 — Additional file 7: Table S6. LOH events in the mutation accumulation experiment. [file 13059_2021_2521_MOESM7_ESM.pdf]

Table S6 List of LOH tracts detected in the mutation accumulation analysis

Mutation accumulation line (hybrid diploid ancestry)	chromosome	Loss-of-heterozygosity (LOH) tract			
		beginning	end	minimum size (bp)	number of SNP markers
K1	chrA	411449	413791	2343	6
L1	chrA	513059	522453	9395	20
L3	chrA	578207	578207	1	1
K1	chrA	948826	948826	1	1
K6	chrA	1222049	1223493	1445	5
K5	chrA	1505212	1506895	1684	9
K3	chrA	1661620	1661620	1	1
K5	chrA	1708548	1708548	1	1
L2	chrA	2188868	2189916	1049	11
L4	chrA	3017907	3017907	1	1
K1	chrA	3066698	3066698	1	1
L6	chrB	514822	514849	28	2
K1	chrC	1258	182642	181385	4190
L4	chrC	281216	281366	151	6
L2	chrC	336505	336505	1	1
L2	chrD	344988	357464	12477	23
L3	chrD	1666276	1675901	9626	7
L3	chrE	830403	830403	1	1
K3	chrE	944151	944151	1	1
L3	chrE	956070	956070	1	1
L6	chrG	21383	21691	309	2
K3	chrG	281226	281272	47	4
