# Supplementary material for: Sex without crossing over in the yeast Saccharomycodes ludwigii
Source: Genome Biol. 2021 Nov 3;22:303. doi: 10.1186/s13059-021-02521-w (PMC8567612; doi:10.1186/s13059-021-02521-w)
Supplement: Supplementary file 8 — Additional file 8: Table S7. List of plasmids. [file 13059_2021_2521_MOESM8_ESM.pdf]

Table S7 List of plasmids generated and used in this study

Plasmid ID[1]	Plasmid Name	Backbone	Features	Purpose	Source/Reference
**Vectors used for plasmid construction**					
Ec-312	pRS415	pRS415	*CEN* vector, *LEU2*, *amp*^{R}	backbone for plasmid construction	[106]
Ec-432	pFA6a-*kanMX4*		*kanMX4*	amplification of the *kanMX4* cassette	[107]
Ec-1579	pFA6a-*hphNT1* (pKS133-6)		*hphNT1*	amplification of the *hphNT1* cassette	[108]
Ec-3108	pYM12-1	pFA6a	*eGFP-kanMX6*	amplification of the *eGFP-kanMX6* cassette	[109]
**Plasmids for construction of the *Sd. ludwigii* isogenic diploid strain (YLFP18-1[2])**					
Ec-4664	pFP6-1	Ec-312	*URA3*	cloning of *Sd. ludwigii URA3*	this study
Ec-4528	pFP2-1	Ec-312	*Δura3::kanMX4*[3]	deletion of *Sd. ludwigii URA3*	this study
Ec-4526	pFP1-1	Ec-312	*Δura3::hphNT1*[4]	deletion of *Sd. ludwigii URA3*	this study
Ec-4648	pFP4-1	Ec-312	*MATα*	cloning of *Sd. ludwigii MATα*	this study
Ec-4727	pMaM64	Ec-312	*Δmatα::Δura3::kanMX4*	deletion of *Sd. ludwigii MATα*	this study
Ec-4682	pMaM55	Ec-312	*MATa*	cloning of *Sd. ludwigii MATa*	this study
**Plasmids for functional analyses of meiotic genes**					
Ec-4672	pFP10-1	Ec-312	*SPO11*	cloning of *Sd. ludwigii SPO11*	this study
Ec-4695	pFP15-1	Ec-312	*Δspo11::kanMX4*[3]	deletion of *Sd. ludwigii SPO11*	this study
Ec-5101	pFP41-1	Ec-312	*RAD51*	cloning of *Sd. ludwigii RAD51*	this study
Ec-5105	pFP42-1	Ec-312	*Δrad51::kanMX4*[3]	deletion of *Sd. ludwigii RAD51*	this study
Ec-5219	pFP44-1	Ec-312	*DMC1*	cloning of *Sd. ludwigii DMC1*	this study
Ec-5225	pFP46-1	Ec-312	*Δdmc1::kanMX4*[3]	deletion of *Sd. ludwigii DMC1*	this study
Ec-4709	pFP17-1	Ec-312	*SAE2*	cloning of *Sd. ludwigii SAE2*	this study
Ec-4725	pFP21-1	Ec-312	*Δsae2::kanMX4*[3]	deletion of *Sd. ludwigii SAE2*	this study
Ec-4963	pFP35-1	Ec-312	*RAP1*	cloning of *Sd. ludwigii RAP1*	this study
Ec-5017	pFP39-1	Ec-312	*RAP1-eGFP-kanMX6*[5]	tagging of *Sd. ludwigii RAP1* with *eGFP*	this study

[1] Database ID numbers of plasmids correspond to their permanent identifiers in the Knop lab collection of *Escherichia coli* host strain DH5α clones.

[2] See Supplementary Table 1.

[3] The *kanMX4* cassette was amplified from plasmid Ec-432.

[4] The *hphNT1* cassette was amplified from plasmid Ec-1579.

[5] The *eGFP-kanMX6* cassette was amplified from plasmid Ec-3108.
