# Supplementary material for: Sex without crossing over in the yeast Saccharomycodes ludwigii
Source: Genome Biol. 2021 Nov 3;22:303. doi: 10.1186/s13059-021-02521-w (PMC8567612; doi:10.1186/s13059-021-02521-w)
Supplement: Supplementary file 9 — Additional file 9: Table S8. List of PCR primers. [file 13059_2021_2521_MOESM9_ESM.pdf]

Table S8 List of DNA oligonucleotides used in this study

Oligo name	Sequence (5’ to 3’)	Template[1]	Purpose
**Construction of the *Sd. ludwigii* isogenic diploid strain (YLFP18-1[2])**			
Sdl.URA3-C	TAACCCTTCTGGTTGTGAAGTC	*Sd. ludwigii* genomic DNA	cloning of *URA3*
Sdl.URA3-D	ATAAGTAATCAAATTTTGGGGGTC		
S1-Sdl.URA3	GAACGAGAAACTCGATAAAACAATACAAATAAAGCAAATCCCAATTTCTATAATGCGTACGCTGCAGGTCGAC	plasmids Ec-432/Ec-1579	deletion of *URA3*
S2-Sdl.URA3	ATCAATACAAATTCATATGAAAATGTTCTGAATCTTTGATTCAAAAAGAAACTTAATCGATGAATTCGAGCTCG		
Sdl.MAT-A5	CAAAAGCTGGAGCTCCACCGCGGTGGCGGCCGCTCTAGAACTAGTGGATCCATGTTGCAGCATCCACCGTTC	*Sd. ludwigii* genomic DNA	cloning of *MATα*
Sdl.MAT-B5	CGTAATACGACTCACTATAGGGCGAATTGGGTACCGGGCCCCCCCTCGAGAGTACAGACAAGTACCGGTCC		
MATalpha-A4	GGCGAATTGGAGGTCAATAAAG	*Sd. ludwigii* genomic DNA	exchange of *MAT* idiomorphs
MATalpha-B6	CGCTACTGAAAGGAACAGACG		
**Functional analyses of meiotic genes**			
SPO11_LongFor	CAAAAGCTGGAGCTCCACCGCGGTGGCGGCCGCTCTAGAACTAGTGGATCCTGTATATTGGGTGATGTTTGGGC	*Sd. ludwigii* genomic DNA	cloning of *SPO11*
SPO11_LongRev	CGTAATACGACTCACTATAGGGCGAATTGGGTACCGGGCCCCCCCTCGAGAACTGTACTTATACTTCCAACTC		
SPO11-S1	GTAAAGTAAAATAAATAAACAAATAAAAAATAATAAAAAAAAATTAATTATGCGTACGCTGCAGGTCGAC	plasmid Ec-432	deletion of *SPO11*
SPO11-S2	TTCATTGGCTTCAAATCTACTAATATGTCAGTTTAATTAAACCCTTCATTCACTAATCGATGAATTCGAGCTCG		
RAD51-F1	CAGTGAATCTGGTGGCAATAC	*Sd. ludwigii* genomic DNA	cloning of *RAD51*
RAD51-R2	GTGTAAGAACCCTCACCATTATC		
S1-RAD51	GAAAAAAGAAAGAAAAAGAAAATAAAAGAGATTTTTCTAAGATATATCAAACTATGCGTACGCTGCAGGTCGAC	plasmid Ec-432	deletion of *RAD51*
S2-RAD51	ATTGTCGCTGGGTATTATATTACAATTATTTTATACGATACAATATATTCTTATTAATCGATGAATTCGAGCTCG		
DMC1-F2	CAGTACCAGTTGGATACCACC	*Sd. ludwigii* genomic DNA	cloning of *DMC1*
DMC1-R2	TGATTTCATTCCCGTGGGCAG		
S1-DMC1	GAAATGTCAGAAACAACCACAACAGGTATAATTACAATTGATGAACTACAAAATTCGTACGCTGCAGGTCGAC	plasmid Ec-432	deletion of *DMC1*
S2-DMC1	ATATATAATGGTAAATAAAAATAAGCTTCACGGATTTGGAATAACATAATAAATCAATCGATGAATTCGAGCTCG		
SAE2_LongFor	CAAAAGCTGGAGCTCCACCGCGGTGGCGGCCGCTCTAGAACTAGTGGATCCTGGATACGGATTGGGTTAATGG	*Sd. ludwigii* genomic DNA	cloning of *SAE2*
SAE2_LongRev	CGTAATACGACTCACTATAGGGCGAATTGGGTACCGGGCCCCCCCTCGAGGCGTGATTTGGTACGGAAGG		

S1-SAE2<br>S2-SAE2	GATCTAAAGCTTTTGCAACTTTTAGGAGTTTTTACACTAACCTAGAGAATACAAATGCGTACGCTGCAGGTCGAC<br>ATTTATTTATTTATTTATTTATTTATTTATTTATTTATTTATTTATTTATTTAATCGATGAATTCGAGCTCG	plasmid Ec-432	deletion of *SAE2*
RAP1_LongFor<br>RAP1_LongRev	CAAAAGCTGGAGCTCCACCGCGGTGGCGGCCGCTCTAGAACTAGTGGATCCAAGGTGTGTTAAGGGCGGAG<br>CGTAATACGACTCACTATAGGGCGAATTGGGTACCGGGCCCCCCCTCGAGTGGATTGGGAGGATCTAATGGTTC	*Sd. ludwigii* genomic DNA	cloning of *RAP1*
RAP1-S1<br>RAP1-S2	CATAACGCTTAAAAACAAAAAAAATATATATAAATACCAGAGAGCAAGGAGATGCGTACGCTGCAGGTCGAC<br>ATATGAGAGAGAAAGGAAGAAAATAAAAAGTGTTCAACTAAAAAACTTGGAACTAATCGATGAATTCGAGCTCG	plasmid Ec-3108	tagging of *RAP1*
**Validation of NCOs**			
For_1599931<br>Rev_1599931	TCATTATTTCCGCCAGGTGC<br>GTGTTTCAGCGTTTGTCGTTG	*Sd. ludwigii* genomic DNA<br>(tetrad 280-283)	validation of NCO<br>(in chrA)
For_113786<br>Rev_113786	TAATGACATGAGCGTGAGAAGG<br>CGGCCAATAACAAAGCATGGG	*Sd. ludwigii* genomic DNA<br>(tetrad 989-992)	validation of NCO<br>(in chrD)

[1] Plasmid (see Supplementary Table 7) or genomic DNA of yeast strain (see Supplementary Table 1).

[2] See Supplementary Table 1.
