# Supplementary material for: Sex without crossing over in the yeast Saccharomycodes ludwigii
Source: Genome Biol. 2021 Nov 3;22:303. doi: 10.1186/s13059-021-02521-w (PMC8567612; doi:10.1186/s13059-021-02521-w)
Supplement: Supplementary file 10 — Additional file 10. Supplementary detailed methods. [file 13059_2021_2521_MOESM10_ESM.pdf]

Supplementary detailed methods

Mating and sporulation of *Sd. ludwigii*

Mating of *Sd. ludwigii* strains was performed by mixing equal numbers of haploid cells of opposite mating types on YPD plates and incubating them at 30 °C for 16 h. The cell mixture was subsequently transferred directly to sporulation medium or was streaked to single colonies on double selective YPD plates that support growth of diploid cells only (in case of strains containing different markers). When wild-type strains without suitable markers were mated, diploid colonies were identified by visual inspection, based on their brittle and matte morphology. For sporulation in liquid cultures, diploid cells were pre-grown in YPD medium to early stationary phase and then transferred to liquid sporulation medium (1% potassium acetate, 1% glucose, 0.25% yeast extract) at an initial density of OD_{600}=0.6. The culture was incubated at 23 °C (with shaking at 230 rpm) for 3 days. For sporulation on plates, freshly grown cells from YPD plates were transferred to sporulation plates (2% w/v potassium acetate, 0.1% glucose, 2% agar) and incubated at 23 °C for up to 5 days.

Sd. ludwigii tetrad dissection

For tetrad dissection, a loopful of sporulated cells were transferred to 100 µl of sterile water and 1 µl of a zymolyase 100T solution (10 mg/ml; Amsbio) was added. The suspension was gently mixed, incubated at room temperature for 10 min, and afterwards stored on ice. Droplets (15 µl) of this suspension were spread in the middle of YPD plates, which were used for tetrad dissection with a dissection microscope (Singer Instruments). Compared to *S. cerevisiae*, tetrads of *Sd. ludwigii* exhibit strong interspore bridges that link pairs of spores (dyads). In order to dissect tetrads, these bridges have to be broken mechanically. This can be tedious, and meticulous manipulation is necessary to prevent spore damage. To facilitate breakage of interspore bridges, the method developed by Yamazaki [35,36] was adopted. This involved placing a hydrated sterile cellophane sheet on the surface of the agar as a solid yet flexible support surface for dissection. Individual tetrads were carefully transferred onto it and spores were manipulated by lateral pushing rather than applying any pressure directly on them, since this could damage them. In addition, we used self-made glass needles with a little dent at one side in order to facilitate separation of dyads. Only with this setup, precise positioning of the dyads and exact application of forces to the bridges did we manage to dissect tetrads without

damaging the spores. For analysis of mutants, we always dissected an equal number of control tetrads from wild-type cells.

Transformation of *Sd. ludwigii*

For preparation of competent cells, a 50-ml YPD culture was inoculated from a saturated starter culture at an initial OD_{600} of 0.1 and was incubated at 30 °C (shaking at 230 rpm) until OD_{600} was in the range 0.8-1.0. Cells were harvested by centrifugation (500 × g for 5 min) and washed once in 25 ml of sterile distilled water and once in 12 ml of LiSorb buffer (100 mM LiAc, 1 M sorbitol, 10 mM Tris-HCl pH 8.0, 1 mM EDTA-NaOH pH 8.0) at room temperature. Cells were resuspended in 360 µl of LiSorb buffer, followed by the addition of 40 µl of denatured carrier DNA solution (salmon sperm ssDNA, 10 mg/ml; Invitrogen). Aliquots of 50 µl of these competent cells were used immediately for transformation (for maximum efficiency) or stored for future use at -80 °C (no snap freezing). For each transformation, an aliquot of competent cells was thawed at room temperature and up to 20 µg of DNA (in max. 5 µl of water) were added. After mixing, 300 µl of buffer SdlLiPEG (50 mM LiAc, 45% w/v PEG-3350, 10 mM Tris-HCl pH 8.0, 1 mM EDTA-NaOH pH 8.0) were added, and the suspension was mixed well before being incubated at room temperature for 2 h. Following this incubation, the sample was subjected to heat shock at 38 °C for 40 min; alternatively, DMSO was added to a final concentration of 15% and heat shock was performed at 38 °C for 15 min. Subsequently, the cells were washed twice in YPD medium (4,000 rpm for 2 min), before resuspension in 2 ml of this medium and incubation at 30 °C for at least 6 h (with shaking at 230 rpm). Finally, cells were pelleted again, resuspended in 100 µl of YPD medium, plated on selective medium and incubated at 30 °C for 3-4 days. Three to six independent transformants were streaked to single colonies on fresh selective medium and the genomic alterations were validated by colony PCR.

To increase transformation efficiency in *Sd. ludwigii*, 180-300 bp-long homology flanks were used, by using *S. cerevisiae* strain ESM356-1 for *in vivo* recombinational cloning of the DNA constructs or the NEBuilder HiFi DNA Assembly Cloning kit (New England Biolabs) for their *in vitro* assembly. High-fidelity DNA polymerases were always used for amplification of PCR cassettes for strain construction, whereas *Taq* DNA polymerases were routinely used for validation of engineered strains using colony PCR.

Construction of isogenic *Sd. ludwigii* strains of opposite mating types

Strain *Sd. ludwigii* NBRC 1722 (*MATalpha*) was used as the background strain for construction of isogenic *Sd. ludwigii* strains of opposite mating types. To generate an isogenic *MATa* strain, a

two-step replacement of the *MATalpha* locus was performed. Briefly, the *URA3* gene of strain NBRC 1722 was replaced with the *kanMX4* cassette. Then, the *MATalpha* locus was replaced with *URA3*, followed by replacement of the *URA3* marker with a cloned copy of the *MATa* locus (from strain NBRC 1723), using selection on 5-FOA. This resulted in strain Sdl-339. All strains and their genotypes are listed in Additional file 1: Table S1.

Pulsed-field gel electrophoresis (PFGE)

Spheroplasts were prepared from *Sd. ludwigii* cells, embedded in agarose plugs, lysed and processed for electrophoresis of intact chromosomes as previously described [36], using the CHEF-DR II Pulsed Field Electrophoresis System (Bio-Rad).

Transmission electron microscopy (TEM)

The glutaraldehyde-$KMnO_4$ fixation method, followed by embedding in Agar 100, was used for electron microscopy of vegetative *Sd. ludwigii* cells as previously described [110].

Ploidy determination using flow cytometry

The cellular DNA content was analyzed by propidium iodide staining following ethanol fixation and flow cytometry on a FACSCanto II (BD Biosciences) instrument, according to standard protocols [111].

Immunostaining of meiotic spreads

For spheroplast formation, 10 ml of a liquid sporulation culture were centrifuged at 700 × g for 5 min and the cell pellet was resuspended in 1 ml resuspension buffer (2% potassium acetate, 0.8 M sorbitol). Following the addition of 20 µl of a 0.5 M DTT solution and 25 µl of a zymolyase 100T (10 mg/ml) solution, the sample was placed on a rotating wheel at 37 °C for digestion of cells; the process was monitored microscopically every 5 min. Cell shape changes during digestion; at first, cells become small and round, then their size increases by 2 to 3-fold and, finally, the cell wall disappears but the cell content is still held together by the plasma membrane. When at least 70% of the cells were at this stage, digestion was arrested by transferring the cells to a 15-ml tube containing 10 ml of ice-cold stop solution (0.1 M MES, 1 M sorbitol, 1 mM EDTA, 0.5 mM $MgCl_2$, pH 6.4). The suspension was then centrifuged at 900 × g for 7 min (at 4 °C), the supernatant was discarded and the cell pellet was gently resuspended in 30 µl of ice-cold stop solution without sorbitol (absence of sorbitol causes hypotonic burst of the cells). This was followed by the addition of 60 µl of fixative (4% formaldehyde, 1.5% sucrose, pH

7.3) and 30 µl of the suspension were pipetted onto a microscope slide previously coated with 0.1% w/v poly-L-lysine. The suspension was spread by tilting the slide, which was followed by the addition of 80 µl of 1% Lipsol and mixing by tilting the slide again. Another 80 µl of fixative were added and the slide was tilted again for mixing. The slide was then dried overnight under a fume hood, and it was subsequently stored in a Coplin jar at -20 °C or processed directly for immunostaining.

Before immunostaining the slide was washed by immersion in PBS buffer for 10 min, which was followed by the addition of 100 µl of blocking buffer (0.5% BSA, 0.2% gelatin) and incubation for 10 min. The slide was covered with a coverslip during the incubation; to remove it before the next step of the procedure, the slide was immersed in PBS and gently shaken. For immunostaining, 40 µl of a primary antibody solution in blocking buffer were added, the slide was covered with a coverslip again and was incubated for 2 h in a humid chamber. The coverslip was then removed in PBS buffer and the slide was washed by immersion in PBS buffer for 5 min. Following that, 40 µl of a secondary antibody solution in blocking buffer were added on the slide, a coverslip was placed and the sample was incubated in a humid chamber for 1 h. For DNA staining, the coverslip was removed in PBS buffer and the slide was washed by immersion in fresh PBS buffer for 10 min (twice). The sample was then mounted with 20 µl of a Hoechst 33258 solution (0.5 µg/ml) in 60% glycerol, a coverslip was added and sealed with nail polish. Single-plane epifluorescence images were acquired using an upright fluorescence microscope and a 100x Plan-Apochromat oil immersion objective. Image processing and merging of channels was performed using ImageJ [112].

Total DNA extraction from *Sd. ludwigii* using phenol-chloroform extraction

Cells were harvested from a liquid culture in YPD medium (OD_{600} = 1), washed in 1 ml of resuspension buffer (0.9 M sorbitol, 0.1 M EDTA pH 7.5), pelleted again by centrifugation and finally resuspended in 0.4 ml of resuspension buffer amended with 1.4 mM β-mercaptoethanol. Subsequently, 20 µl of a zymolyase 100T solution (10 mg/ml) were added, and the suspension was incubated at 37 °C for 30 min. Digested cells were layered over a sorbitol cushion (1.8 M) in a 50-ml tube, which was then centrifuged at 2,000 rpm for 15 min (at 4 °C). Sorbitol was discarded and the pellet was resuspended in 0.4 ml TE buffer (pH 8.0) after the addition of 90 µl of a solution containing 0.5 M EDTA, 2 M Tris and 10% SDS (pH 8.0). The sample was incubated at 65 °C for 30 min before the addition of 80 µl of a 5 M potassium acetate solution and incubation at 4 °C for at least 1 h (usually overnight). The sample was then centrifuged at 13,000 rpm for 15 min and the supernatant was mixed with 1 ml of ethanol. The precipitate was

recovered by brief centrifugation (5 s) at maximum speed, and the pellet was then rinsed with 70% ethanol, air-dried and gently resuspended in 0.5 mL TE buffer (pH 8.0). The sample was centrifuged again (13,000 rpm for 15 min) to remove any insoluble material, and 5 μl of an RNase A solution (5 mg/ml) were added to the supernatant, which was followed by incubation at 37 °C for 30 min. Two consecutive phenol-chloroform-isoamyl alcohol (25:24:1) extraction steps followed (the 5Prime Phase Lock Gel system was used for maximum recovery), and 0.6-0.8 volumes of isopropanol were added for DNA precipitation by centrifugation (13,000 rpm for 10 min, at 4 °C). Total DNA was washed with 70% ethanol, dried and resuspended in 50 μl molecular grade water. For removal of polysaccharides, the DNA sample was mixed with 0.1 volume of a hexaamminecobalt(III) chloride solution (100 mM) and centrifuged at 13,000 rpm for 5 min. The pellet was washed with 200 μl of water and dissolved in 50-100 μl of exchange buffer (100 mM EDTA pH 8.0, 2 M guanidinium thiocyanate) at 37 °C for 1 h. Once the pellet was completely dissolved, total DNA was precipitated, washed, dried and finally resuspended in 50 μl TE buffer (pH 8.0).

Gene prediction and annotation

Firstly, we used the Yeast Genome Annotation Pipeline (YGAP [113]), which is based on homology and gene synteny information from previously analyzed yeast species, available in the Yeast Gene Order Browser (YGOB) database [114]. Secondly, AUGUSTUS [115] (v3.2.1) was trained on the latest (as of September 2018) available protein datasets of several annotated yeast species (*Saccharomyces cerevisiae*, *Naumovozyma dairenensis*, *Tetrapisispora blattae*, *Vanderwaltozyma polyspora*, *Kluyveromyces lactis*, *Eremothecium gossypii*, *Hanseniaspora uvarum*) and all resulting training datasets were independently used for *ab initio* gene prediction in *Sd. ludwigii*. Thirdly, the MAKER pipeline [116] (v2.30), with predictors Augustus [115] (v2.5.5), Genemark-ES [117] (v2.3) and SNAP [118], was used for gene prediction using the protein datasets of all yeast species available in the YGOB database [114] as evidence. Custom scripts were then used for comparing the aforementioned prediction datasets and integrating all non-overlapping predicted gene models into a union dataset. The most probable gene model was retained for each gene, based on manual curation (using the genome browser IGV [119]; v2.6.2) and BLAST+ [120] (v2.8.0) comparisons. Finally, all genomic regions without predicted genes were extracted from the genomic sequence (using BEDtools [121]; v2.27.0), and custom scripts were used for retrieving all open reading frames in these regions that were predicted to code for proteins with a minimum size of 30 aa. These were analysed using BLAST+ [120] (v2.8.0) and were retained in the final gene prediction

dataset only if they were homologous to known proteins of other yeast species or similar to conserved hypothetical proteins. Furthermore, we used tRNAscan-SE [122] (v2.0) for the identification of tRNA genes, BLAST+ [120] (v2.8.0) and RNAmmer [123] (v1.2) for the annotation of rRNA genes, and the program cmscan (v1.1) of Rfam [124] (v12.1) for prediction of other RNA genes. For the assessment of gene prediction completeness, BUSCO [125] (v3) was used, against the "Saccharomycetales" and "Fungi" databases of conserved single-copy orthologs. Functional annotation and Gene Ontology (GO) assignment of the final gene dataset was performed using the software suite Blast2GO PRO [126] (v5), integrating evidence from BLAST+ [120] (v2.8.0), InterProScan [127] (v5) and eggnog [128] (v4.5) searches, and manual curation for resolving conflicting results. The consensus features of point centromeres of Saccharomycetaceae representatives, that were compared to the *Sd. ludwigii* predictions, were retrieved from [129].

Analysis of meiotic gene content

The meiotic gene complement of *Sd. ludwigii* was compared to that of the 20 Saccharomycetaceae species that are included in the YGOB (v7) database, as well as to that of 9 Pichiaceae species that are included in the Methylotroph Gene Order Browser (MGOB) database [130] (http://mgob.ucd.ie). In order to gain a better understanding of how the missing meiotic genes from *Sd. ludwigii* evolved along the yeast phylogeny, we also studied the distribution of their homologs in the families Saccharomycodaceae and Phaffomycetaceae. For this, we used the *S. cerevisiae* protein sequences as queries in tBLASTn [120] searches (with the Expect threshold set at 50) against all available genome assemblies from these two families in the NCBI Genome database on the 18th August 2019 (i.e., 28 genome assemblies from 18 *Hanseniaspora*/*Kloeckera* species or unclassified strains from the Saccharomycodaceae, and 42 assemblies from 33 species/strains of the Phaffomycetaceae, excluding the *Komagataella* species that have been classified in the Pichiaceae clade [131]). Representative positive hits were further examined using reciprocal BLASTp searches against the annotated *S. cerevisiae* proteome (SGD [132]). In those cases of putative homologs with marginally detectable similarity (often limited to short protein regions), synteny with *S. cerevisiae* S288C (YGOB database; v7) was also investigated and used as additional evidence for inferring homology. To confirm the absence of detectable homologs of *MER1* from the *Hanseniaspora* species (family Saccharomycodaceae), we used the synteny information from *S. cerevisiae* to identify the expected positions in the genomes of 10 representative species (i.e., *H. vineae*, *H. osmophila*, *H. occidentalis*, *H. lachancei*, *H. pseudoguilliermondii*, *H. guilliermondii*, *H. opuntiae*, *H.*

thailandica, *H. jakobsenii* and *H. singularis*) and we analysed all predicted ORFs in these regions for weak similarity to the *S. cerevisiae* Mer1 sequence. The online search engine of the Pfam database [133] (v32.0; https://pfam.xfam.org) was used for the identification of protein domains. Prediction of intrinsically unstructured regions in proteins was performed with the online tool IUPred2A [134] (https://iupred2a.elte.hu).

Genome alignment

For genomic comparisons between *Sd. ludwigii* strains we used the software MUMmer [135] (v4.0.0). Alignments of genomic sequences were performed using the program nucmer (with the --maxmatch option). The output files were passed on to the script mummerplot for visualization. Comparisons between genomes were plotted as chord diagrams using the R package circlize [136] (v0.4.6). The dnadiff (v1.3) wrapper of the genome alignment system MUMmer [135] was used for the calculation of average nucleotide identity between whole genomes. Structural variations were called using Sniffles [137] (v1.0.10) from the sorted output of the BWA-MEM aligner [138] (v0.7.17).

Other genomic analyses

We used RepeatMasker (v4.0.7; http://www.repeatmasker.org) with combined Dfam [139] (v2.0) and Repbase [140] (Genetic Information Research Institute - GIRI) databases, NCBI RMBLAST [120] (v.2.6.0+) as the search engine and Tandem Repeats Finder [141] (v4.0.9), for identification of genomic repeats, low-complexity regions and transposable elements. Other genomic data analyses were performed using R [142] and the Bioconductor framework [143].
