# Supplementary material for: Sex without crossing over in the yeast Saccharomycodes ludwigii
Source: Genome Biol. 2021 Nov 3;22:303. doi: 10.1186/s13059-021-02521-w (PMC8567612; doi:10.1186/s13059-021-02521-w)
Supplement: Supplementary file 11 — Additional file 11. review history [file 13059_2021_2521_MOESM11_ESM.docx]

Review History

**First round of review**

**Reviewer 1**

**Are you able to assess all statistics in the manuscript, including the appropriateness of statistical tests used?**

No, I do not feel adequately qualified to assess the statistics.

**Comments to author:**

Overview: This is an interesting, well prepared and well written study into the sexual life cycle of the yeast Saccharomycodes ludwigii. The text is clear and easy to follow. On the main part, the experiments are well controlled, and the findings convincing - namely that there is no evidence of crossing over in meiosis despite retention of pro-DSB factors (and demonstrated cytological markers of DSBs, such as Spo11-dependent Rad51 foci). The LD analyses corroborate these conclusions, although I am not an expert in these statistical interpretations. The findings will likely be of interest to meiosis researchers. I have a few comments that I would urge the authors to consider/address in order to increase the clarity of the presented work.

Minor: Line 86. Crossover rate is even greater in S. cerevisiae with the "long" chromosomes like Chr4, having as many as 15 COs despite being a fraction of the length of a pombe chromosome. (If required please refer to data from Martini, Llorente, Fung, Neale, labs.)

Major: Line 226, and Reference 39. I think the statement that Spo22/Zip4 only has a minor role in S. cerevisiae is a rather misleading. It is my understanding that Zip4 (which I think is the more-commonly used name for this gene by the meiosis community) is a major component of the class I CO pathway, which involves other CO-specific factors such as Zip1,2,3, Mlh1,3, Msh5-6 and Exo1 (plus others). Zip4 is also required for Class I CO formation in other species (e.g. https://pubmed.ncbi.nlm.nih.gov/17530928/). As such, it seems possible that, were ludwigii entirely dependent on class I CO formation (i.e. no 'backup' class II pathway), this would be sufficient to abrogate all CO detection. It would be helpful if the authors could respond to this point and/or update the main text/discussion as appropriate.

Minor: Line 279. It is incorrect to refer to Spo11 as an endonuclease - it is a topoisomerase-like transesterase. This sentence is particularly confusing due to describing Sae2 as an endonuclease on line 277, which is also not correct. Sae2 promotes the nuclease activity of Mre11 (https://pubmed.ncbi.nlm.nih.gov/25231868/). Please correct these points to avoid reader confusion.

Minor: Line 291. It is rather unusual to refer to the major role of Dmc1 as an inhibitor of Rad51's strand exchange activity. Whilst there is genetic evidence for this, the primary role, of Dmc1, surely, is to directly promote interhomologue recombination. Are there any other known observations of extended Rad51 foci in dmc1D (in other organisms) that might corroborate this interpretation?

Major: Line 324, SNP density is often reported as both mean (and median) distance between adjacent SNPs, and as % sequence divergence between the strains. It would be helpful to the reader if this information were added here. By my quick calculations these appear to be mean distances of only <34 bp between SNPs, and a % divergence of >1.5% on the indicated high diversity regions. Relative to similar studies in S. cerevisiae hybrids (e.g. https://journals.plos.org/plosgenetics/article?id=10.1371/journal.pgen.1002305; ~1 SNP per 200 bp, and an overall divergence of 0.5%), the reported level of sequence divergence is very high, and it is reasonable to expect it will impede meiotic recombination reactions—perhaps, especially, class I COs, which appear more sensitive to polymorphisms (https://www.biorxiv.org/content/10.1101/480418v2). Of course, from the same arguments, recombination between parts of chromosomes A, B, and all of chromosome D, would not be expected to be affected.

Line 331. Please clarify what is the "second parent's lineage".

Minor: Fig 5C. What do the white vertical gaps indicate? Are these regions where there are no SNPs? Perhaps low and high density SNP areas could be indicated on these figures?

Minor: Line 349, Fig 5f. This analysis requires some form of quantification. How many cells were observed? What fraction of these showed colocalization? None?

**Reviewer 2**

**Are you able to assess all statistics in the manuscript, including the appropriateness of statistical tests used?**

Yes, and I have assessed the statistics in my report.

**Comments to author:**

In this study, the authors generated high quality genome sequence for Sd. ludwigii, and additional sequences of tetrads and mutation accumulation lines, attempted to address questions regarding suppressed recombination during meiosis in this species. The manuscript addresses an interesting question in the field.

The methods are appropriate to the aims of the study. They are well described.

The main claim is that the high rate of intratetrad mating is coupled with extreme suppression of meiotic crossing over throughout its genome. I am not convinced that this is adequately supported by their data. The core of my concern is on the data supporting the suppression of meiotic crossing. They analyzed 7 tetrads, and the tetrads can be informative. To demonstrate suppression of crossing (or anything in low frequency), a small number of samples will not (almost never) be convincing. The authors should consider sequencing more spores from a sufficient number of tetrads to make the study convincing. Since this is the core of the study, informative quantitative comparison is essential.

There is a value of regeneration of genome sequencing using PacBio. Their genome has a lower GC content than published ones in previous studies. The authors may want to mention the differences in sequencing methods among these genomes.

The LD decay needs to be interpreted carefully. The authors did not compare the heterozegosity level and population diversity among different species. Also, different species may have ver different frequency of sexual reproduction in their life cycle. The different LD decay pattern could have been influenced by several factors.

The mutation accumulation analysis does not seem to be directly relevant in the study. The results are mostly routine analysis for mutation accumulation studies without much comparison. To make it more relevant, the authors may want to compare their results against results in other species. With their hybrid strains, it would be interesting to examine the conversion patterns of the heterozygous sites.

The manuscript needs first to show convincing data for suppression of meiotic crossing after mating. Once established, it does have advance over previous studies. Before that, it is hard to see the significant advance of their study.

**Authors Response**

**Point-by-point responses to the reviewers’ comments:**

Reviewer #1

Overview: This is an interesting, well prepared and well written study into the sexual life cycle of the yeast Saccharomycodes ludwigii. The text is clear and easy to follow. On the main part, the experiments are well controlled, and the findings convincing - namely that there is no evidence of crossing over in meiosis despite retention of pro-DSB factors (and demonstrated cytological markers of DSBs, such as Spo11-dependent Rad51 foci). The LD analyses corroborate these conclusions, although I am not an expert in these statistical interpretations. The findings will likely be of interest to meiosis researchers. I have a few comments that I would urge the authors to consider/address in order to increase the clarity of the presented work.

Minor: Line 86. Crossover rate is even greater in S. cerevisiae with the "long" chromosomes like Chr4, having as many as 15 COs despite being a fraction of the length of a pombe chromosome. (If required please refer to data from Martini, Llorente, Fung, Neale, labs.)

*-	Added (lines 84-87): “These range from one obligatory CO per chromosome in Caenorhabditis elegans [14] to 10 or more in Schizosaccharomyces pombe [15], and up to 15 in the longest chromosomes of S. cerevisiae [16].” (new text and Refs. in blue)*

Major: Line 226, and Reference 39. I think the statement that Spo22/Zip4 only has a minor role in S. cerevisiae is a rather misleading. It is my understanding that Zip4 (which I think is the more-commonly used name for this gene by the meiosis community) is a major component of the class I CO pathway, which involves other CO-specific factors such as Zip1,2,3, Mlh1,3, Msh5-6 and Exo1 (plus others). Zip4 is also required for Class I CO formation in other species (e.g. https://pubmed.ncbi.nlm.nih.gov/17530928/). As such, it seems possible that, were ludwigii entirely dependent on class I CO formation (i.e. no 'backup' class II pathway), this would be sufficient to abrogate all CO detection. It would be helpful if the authors could respond to this point and/or update the main text/discussion as appropriate.

*-	We are particularly thankful to the reviewer for bringing to our attention this point, which supports our hypothesis that loss of Mer1 could have mediated the extreme suppression of crossing over in Sd. ludwigii (i.e., through dysregulation of Spo22/Zip4, whose gene retains a predicted intron despite the loss of its splicing activator Mer1).*

*Note to the reviewer: we have done preliminary analyses, which support this idea further. Some of this preliminary data (not for publication) are included below:*

*i.	Zip1 immunostaining in meiotic spreads of Sd. ludwigii revealed synaptonemal complexes, but with markedly less elongated structures than in S. cerevisiae (panel a, below); this could indicate defects in Zip1 polymerization, which in S. cerevisiae depends on Spo22/Zip4, and*

*ii.	deletion of msh4 (class I COs) severely compromised Sd. ludwigii meiosis, whereas absence of Mms4 (class II COs) had no effect on spore viability (panel b, below); this would be consistent with the hypothesis that the class II pathway is not important in meiosis of this species.*

*We plan to continue this work and include this and more data in a future publication.*

*-	Accordingly with the reviewer’s suggestion, the main text has been revised, and a relevant paragraph has been added to the Discussion:*

*Line 221: “…SPO22 (ZIP4)…”.*

*Lines 224-226: “However, the Mer1 regulon of Sd. ludwigii appears largely rescued, as no introns were detected in 3 of its target genes (AMA1, HFM1 and REC107), while SPO22 has only a minor role in S. cerevisiae meiosis retains a predicted intron [39].”.*

*Lines 553-568:*

*“Further support for our hypothesis that loss of MER1 could have mediated the extreme suppression of meiotic crossing over may be provided by the finding that one of its splicing targets, i.e., SPO22 (ZIP4), appears to retain an intron in Sd. ludwigii. This gene codes for a synapsis initiation complex component that is required for proper SC formation, chromosome synapsis and class I (interfering) COs in S. cerevisiae [83]. Although Zip4 homologs of other organisms (A. thaliana, rice and mouse) have only minor roles in the completion of synapsis, the important Zip4 function in interfering crossing over appears conserved [84–86]. Therefore, potential dysregulation of Zip4 due to the loss of Mer1 from Sd. ludwigii could lower its crossing over levels, or even cause their absence in case the class II CO pathway is not functional in this species. In accordance with this hypothesis, our preliminary investigations of Sd. ludwigii meiotic proteins revealed: i. markedly less elongated SC structures than in S. cerevisiae, possibly indicating defects in Zip1 polymerization, which in S. cerevisiae depends on Spo22/Zip4 [83]; and ii. while deletion of msh4 (class I CO formation [87]) led to remarkably defective meiosis, absence of Mms4 (class II crossing over [88]) had no effect on spore viability, which would be consistent with the idea that the class II pathway may be unimportant in Sd. ludwigii meiosis.”.*

Minor: Line 279. It is incorrect to refer to Spo11 as an endonuclease - it is a topoisomerase-like transesterase. This sentence is particularly confusing due to describing Sae2 as an endonuclease on line 277, which is also not correct. Sae2 promotes the nuclease activity of Mre11 (https://pubmed.ncbi.nlm.nih.gov/25231868/). Please correct these points to avoid reader confusion.

*-	Corrected/clarified:*

*Line 72: “Following pairing of homologous chromosomes, meiotic recombination is initiated in prophase I with the formation of DNA double-strand breaks (DSBs) by the topoisomerase-like protein transesterase Spo11 [10].”*

*Lines 273-278: “Since S. cerevisiae Δspo11 mutants also exhibit defects in chromosome synapsis [10], we also deleted SAE2, an endonuclease a regulator promoting the dsDNA endonuclease activity of Mre11 in the Mre11-Rad50-Xrs2 complex that operates downstream of Spo11 in DSB processing [53]. and cleavage of DNA-Spo11 intermediates [53]. This led to a similar sporulation defect to that of the Δspo11 strain (Additional file 2: Fig. S3), suggesting a role for the endonuclease function of Spo11 in catalyzing DSB formation in Sd. ludwigii meiosis.”.*

Minor: Line 291. It is rather unusual to refer to the major role of Dmc1 as an inhibitor of Rad51's strand exchange activity. Whilst there is genetic evidence for this, the primary role, of Dmc1, surely, is to directly promote interhomologue recombination. Are there any other known observations of extended Rad51 foci in dmc1D (in other organisms) that might corroborate this interpretation?

*-	The interpretation of Dmc1 as an inhibitor of Rad51’s strand exchange activity was removed, and the text was revised/clarified (lines 287-292): “We also performed Rad51 immunostaining in meiotic spreads of Sd. ludwigii cells deleted for DMC1, a ubiquitous meiosis-specific recombinase involved in DSB repair as well as in promoting interhomolog interactions. In this case, we observed elongated, filamentous foci (Fig. 4c), which is consistent with the anticipated function of Dmc1 as an inhibitor of the Rad51 strand exchange activity [54] could be consistent with the facts that in S. cerevisiae the filaments of both proteins often co-occupy the same ends of meiotic DSBs [54], and absence of Dmc1 leads to persistent Rad51 foci, which accumulate and become less punctate with time [55].”.*

Major: Line 324, SNP density is often reported as both mean (and median) distance between adjacent SNPs, and as % sequence divergence between the strains. It would be helpful to the reader if this information were added here. By my quick calculations these appear to be mean distances of only <34 bp between SNPs, and a % divergence of >1.5% on the indicated high diversity regions. Relative to similar studies in S. cerevisiae hybrids (e.g. https://journals.plos.org/plosgenetics/article?id=10.1371/journal.pgen.1002305; ~1 SNP per 200 bp, and an overall divergence of 0.5%), the reported level of sequence divergence is very high, and it is reasonable to expect it will impede meiotic recombination reactions—perhaps, especially, class I COs, which appear more sensitive to polymorphisms (https://www.biorxiv.org/content/10.1101/480418v2). Of course, from the same arguments, recombination between parts of chromosomes A, B, and all of chromosome D, would not be expected to be affected.

*-	We are grateful to the reviewer for calling our attention to this important point, which had not received sufficient emphasis in the original submission. In our revised manuscript, we added all necessary details and statistics in the main text and the updated Supplementary Figure 4b. In addition, the relation between sequence divergence and recombination, and its implications for our analysis, are also mentioned:*

*Lines 323-332: “Variant calling between the two strains resulted in a total of 199,392 199,356 high-quality SNPs, with an overall sequence divergence of 1.59%, while the mean and median distances between adjacent SNPs were 62 and 16 bp, respectively. Notably, heterogeneity was observed in SNP density across the genome, with the higher-heterozygosity genomic compartments (i.e., chrC, chrF, chrG, and parts of chrA and chrB; 44.6% of the genome) exhibiting on average 3.3% divergence (mean/median distance between SNPs: 38/16 bp), whereas the remaining, less divergent parts of the genome (i.e., chrD, chrE, and the remaining parts of chrA and chrB; 55.4% of the genome) displayed on average 0.2% sequence divergence, and 470/201 bp between SNPs (mean/median distance) with an average density of 34 SNPs per kb of genomic sequence on chrC, chrF, chrG and parts of chrA and chrB, whereas the remaining parts of the genome exhibited lower heterozygosity levels, of approx. 3 SNPs per kb on average (details per chromosome are provided in Additional file 2: Fig. S4b).”.*

*Lines 349-357: “High sequence divergence between homologous chromosomes is known to lower the probability of recombination, perhaps especially of class I interfering COs, due to the mismatch repair system that rejects recombination intermediates and redirects them toward NCOs or sister-chromatid recombination [57,58]. This could probably impede recombination in the high-diversity genomic regions, but the low-heterozygosity parts, which constitute more than half of the hybrid genome, should not be affected, since their divergence levels are substantially lower than those used in previous yeast studies [40,43,59,60]. Despite the particularly high resolution of our analysis in the higher-diversity regions, we detected only 2 NCOs in total, which only leaves open the possibility of sister-chromatid repair.”.*

Line 331. Please clarify what is the "second parent's lineage".

*-	Done (lines 334-337): “The same was observed when we extended our analysis to 2 tetrads from a cross between 2 spores of the second parents’ lineage another diploid strain (spores 100 and 102 originating from the wild-type isolate CBS 5929; Additional file 1: Table S1; Additional file 2: Fig. S4a; Fig. S4b)”.*

Minor: Fig 5C. What do the white vertical gaps indicate? Are these regions where there are no SNPs? Perhaps low and high density SNP areas could be indicated on these figures?

*-	Yes, white gaps indicate genomic regions that are devoid of SNP markers. Low- and high-density SNP areas are now indicated for clarity (updated Fig. 5c and legend, lines 377-378: “Genomic regions with high and low levels of sequence divergence are indicated with solid and dashed lines (underneath), respectively.”).*

Minor: Line 349, Fig 5f. This analysis requires some form of quantification. How many cells were observed? What fraction of these showed colocalization? None?

*-	Added (lines 363-368):*

*“To investigate the possibility of crossing over in telomeric or telomere-proximal regions, we used meiotic chromosome spreads to compare the localization of GFP-tagged Rap1, a cytological marker of telomeres [61], and that of Rad51 foci as an indicator of meiotic DSBs [8]. If subtelomeric DSBs mediated chromosome interactions for meiosis I, we would expect to have at least one Rad51 dot per chromosome at or near a telomere. Since we only detect approx. 10 Rad51 foci per nucleus, we would expect to see more than half of the Rad51 dots colocalizing with Rap1. This was never the case (Fig. 5f) in 17 investigated nuclei. Rare colocalization of a single Rad51 dot with Rap1 could be expected to happen by chance, since Rap1 does not only localize to telomeres, but also to other chromosomal regions [62]. Therefore, initiation of recombination is not biased towards telomeres or adjacent regions in Sd. ludwigii, and the extreme suppression of crossing over appears to affect the entire genome.”*

Reviewer #2

In this study, the authors generated high quality genome sequence for Sd. ludwigii, and additional sequences of tetrads and mutation accumulation lines, attempted to address questions regarding suppressed recombination during meiosis in this species. The manuscript addresses an interesting question in the field.

The methods are appropriate to the aims of the study. They are well described.

The main claim is that the high rate of intratetrad mating is coupled with extreme suppression of meiotic crossing over throughout its genome. I am not convinced that this is adequately supported by their data. The core of my concern is on the data supporting the suppression of meiotic crossing. They analyzed 7 tetrads, and the tetrads can be informative. To demonstrate suppression of crossing (or anything in low frequency), a small number of samples will not (almost never) be convincing. The authors should consider sequencing more spores from a sufficient number of tetrads to make the study convincing. Since this is the core of the study, informative quantitative comparison is essential.

*-	We do not agree with the reviewer on this, but we do recognize that we did not explain/discuss it in sufficient depth in our original submission. Our analysis of 7 Sd. ludwigii tetrads yielded 0 crossovers (COs). The same analysis of 7 tetrads of other, previously studied, yeast species would detect: ~630 COs in S. cerevisiae (Mancera et al., 2008), ~315 COs in Schizosaccharomyces pombe (Munz et al., 1989), ~175 COs in Komagataella phaffii (Braun-Galleani et al., 2018), and ~125 COs in Lachancea kluyveri (Brion et al., 2017). The latter, notably, is the yeast with the lowest so far known number of meiotic COs. Furthermore, in none of these species would a single meiotic event finish with all chromosomes exhibiting achiasmate segregation. We believe that these considerations suffice to demonstrate, beyond any scientific doubt, that our dataset is adequately large to provide statistically robust comparisons and to fully support our interpretation: that rare crossing over cannot be excluded, but it is definitely extremely suppressed.*

*-	This conclusion is in full agreement with the older genetic evidence from marker segregation studies (Yamazaki et al., 1976; Yamazaki & Oshima, 1996), which suggested for the first time that crossing over in this species must be extremely rare (in their datasets, they identified signatures of COs in only 0.9% of their analyzed tetrads). Although the low resolution of their experiments could have underestimated the real frequency, these data clearly suggest that even if a much larger number of tetrads were analyzed, no COs would most probably be detected.*

*-	Finally, we would like to explain that experimentation with the non-model yeast Sd. ludwigii (especially tetrad dissection and DNA isolation for NGS) remains a particularly time-consuming and demanding endeavor that requires substantial resources. Therefore, these practical limitations unavoidably dictate that experiments can be pursued only if there are solid scientific reasons to justify the necessity.*

*-	We added to our revised manuscript a brief summary of the main points of this discussion here, to clarify the robustness of our conclusions:*

*Lines 599-606: “Evidence from the older marker segregation studies [35,36] suggested, indeed, that meiotic crossing over could still occur in Sd. ludwigii, but at extremely low frequencies (i.e., in 0.9% of their analyzed tetrads). Even if one assumes that the low resolution of their experiments led to a significant underestimation of that frequency, our higher-resolution data and the comparison with other yeasts clearly demonstrate that COs are extremely suppressed in Sd. ludwigii meiosis: analysis of its 7 tetrads yielded no COs, in comparison to ~630 expected COs in S. cerevisiae [59], ~315 in S. pombe [91], ~175 in K. phaffii [40] and ~125 in L. kluyveri [43].”.*

There is a value of regeneration of genome sequencing using PacBio. Their genome has a lower GC content than published ones in previous studies. The authors may want to mention the differences in sequencing methods among these genomes.

*-	To the best of our knowledge, there is no previously published genome with a higher GC content than that of our assembly. We are aware of only one strain previously sequenced and assembled (UTAD17), and its two assemblies (Tavares et al., 2018 in Microbiol. Resour. Announc.; and Tavares et al., 2021 in BMC Genomics; https://www.ncbi.nlm.nih.gov/genome/browse/#!/eukaryotes/11016/Saccharomycodes%20ludwigii) have GC content values of 31.2% and 30.7%, which are essentially the same as the one observed in our strain (i.e. 30.9%). The estimation for another sequenced strain (for which, however, there is no reported genome assembly) is also quite similar (i.e., 33.6%) (Nguyen et al., 2020 in Mol. Biol. Evol.). We would be grateful if the reviewer could point us to any additional genomes that we might not be aware of.*

The LD decay needs to be interpreted carefully. The authors did not compare the heterozegosity level and population diversity among different species. Also, different species may have ver different frequency of sexual reproduction in their life cycle. The different LD decay pattern could have been influenced by several factors.

*-	We totally agree with the reviewer that the LD decay analysis has limitations and should be interpreted with caution, since its patterns are influenced by many population genetic factors. Unfortunately, most of these parameters are unknown for the majority of yeast species, particularly for those that have attracted little research attention so far, such as Sd. ludwigii. For this species, there is extremely limited previous genome sequence information, and the available sample size of wild-type isolates in the course of our study was very limited. Therefore, we believe that an attempt to estimate population genetic parameters with so limited data would not be meaningful, if not unreliable and misleading. For these reasons, we have been very careful with the interpretation of our LD decay results, which show an extreme behavior of Sd. ludwigii when compared to other species. We do not suggest or imply whatsoever that this result is conclusive on its own regarding the effect of recombination. Nevertheless, we included it in our paper, since an extreme behavior was observed, which simply adds another piece of evidence to our overall conclusion of extremely suppressed meiotic recombination in this species. Our only statement/interpretation of this finding in the manuscript clearly reflects this idea: “These results are consistent with the hypothesis of absent or very rare meiotic recombination in Sd. ludwigii over evolutionary time, which would decrease the overall LD levels and decelerate its decay.” (lines 425-427).*

The mutation accumulation analysis does not seem to be directly relevant in the study. The results are mostly routine analysis for mutation accumulation studies without much comparison. To make it more relevant, the authors may want to compare their results against results in other species. With their hybrid strains, it would be interesting to examine the conversion patterns of the heterozygous sites.

*-	We do not agree with the reviewer that the mutation accumulation analysis is not relevant to our work. The goal of this study was not only to validate the previous hypothesis of absent meiotic recombination in this species, but also to investigate potential molecular and evolutionary causes for the suppression of meiotic recombination. Firstly, an attractive hypothesis was that a possibly elevated mutation rate could function as a compensatory mechanism for the generation of genetic diversity in the absence of meiotic recombination in this species. Or, even more interestingly serve as a driver of achiasmate meiosis, according to implications of previous research (Keller & Knop, 2009). Secondly, we reasoned that a significant AT bias of spontaneous mutations could be the reason for the unusually low GC content of the Sd. ludwigii genome. Therefore, we undertook the mutation accumulation analysis to directly investigate these two hypotheses by determining experimentally the genome-wide mutation rate and AT bias. We successfully achieved both goals, as presented and discussed in the manuscript.*

*-	In our opinion, a detailed comparison of these results with other species, especially since meiotic recombination frequencies are known for only very few of them, would be largely irrelevant to the goals and scope of our study. In addition, there is a publication (cited in our manuscript: Nguyen et al., 2020 in Mol. Biol. Evol.) where a similar analysis of another Sd. ludwigii strain has already been compared to other species.*

*-	We did analyze the conversion patterns of heterozygous sites in our hybrid strain, which resulted in the detection of a number of LOH tracts. This information is in the manuscript, presented in the last paragraph of the Results (lines 488-505), and in Table S6. There is also a relevant paragraph in our Discussion (lines 618-625).*

The manuscript needs first to show convincing data for suppression of meiotic crossing after mating. Once established, it does have advance over previous studies. Before that, it is hard to see the significant advance of their study.

*-	We disagree with the reviewer. In this work, we re-investigate a hypothesis from the ‘30s and ‘70s, but using functional experimentation and analyses of far higher resolution than before, in the light of the status quo knowledge about yeast meiosis. Our study provides molecular insights and evidence for formulating evolutionary hypotheses that were clearly impossible in the earlier studies.*

**Second round of review**

**Reviewer 1**

I thank the reviewers for their considered responses and the modifications/additions they have made to the manuscript. This is an interesting study that I am sure will motivate further investigation in the future.

**Reviewer 2**

My main concern was the limited number of tetras, which has been addressed. I have no further comments
